# An engineered insulin analog with dual insulin and IGF-1 receptor agonism and distinct signaling

**DOI:** 10.1126/sciadv.aeb7558

**Published:** 2026-05-15

**Authors:** Irena Selicharová, Nicholas S. Kirk, Anna Kertisová, Marta Lubos, Katarína Mitrová, Terezie Ticháčková, Lenka Žáková, Martina Chrudinová, Jana Březinová, Karel Harant, Jan Voldřich, Miroslav Hájek, Derek M. Huffman, Jiří Jiráček

**Affiliations:** ^1^ Institute of Organic Chemistry and Biochemistry, Czech Academy of Sciences, Flemingovo n. 2, 16000 Praha 6, Czech Republic.; ^2^ WEHI, 1G Royal Parade, Parkville, Victoria 3052, Australia.; ^3^ Department of Medical Biology, Faculty of Medicine, Dentistry, and Health Sciences, University of Melbourne, Parkville, Victoria, 3010, Australia.; ^4^ Department of Genetics and Microbiology, Faculty of Science, Charles University, 12840 Prague 2, Czech Republic.; ^5^ Department of Molecular Pharmacology, Albert Einstein College of Medicine, Bronx, NY 10461, USA.; ^6^ Department of Medicine, Albert Einstein College of Medicine, Bronx, NY 10461, USA.; ^7^ Institute for Aging Research, Albert Einstein College of Medicine, Bronx, NY 10461, USA.

## Abstract

Insulin and insulin-like growth factors (IGF-1 and IGF-2) regulate metabolism, growth, and development via related receptors. In contexts such as brain function or fetal development, coordinated signaling by all three hormones is essential. We report the engineering of [GluB10, D-HisB24, GlyB31, TyrB32]-insulin (**1**_Ins_), an analog with high affinity for IR-A, IR-B, and especially IGF-1R. **1**_Ins_ binds IGF-1R ~1000-fold more strongly than native insulin, approaching IGF-1 levels. Cryo–electron microscopy structures reveal how minimal substitutions in **1**_Ins_ enable effective binding to both IR-A and IGF-1R. In neuronal cells, **1**_Ins_ robustly activates both IR and IGF-1R pathways, promotes survival, and exceeds native ligands in neuroprotective assays. In vivo, **1**_Ins_ regulates glucose effectively in mice and rats. Phosphoproteomic profiling confirms dual pathway activation and identifies targets specific to **1**_Ins_. These findings demonstrate that rational design of dual-receptor agonists can yield potent, versatile ligands with therapeutic promise in metabolic control, neuroprotection, and regeneration.

## Teaser

A designer insulin analog co-activates insulin and IGF-1 receptors, offering unexplored prospects for therapy.

## INTRODUCTION

Insulin and insulin-like growth factors (IGF-1 and IGF-2) are key regulators of growth and metabolism in animals (*1*, *2*). Insulin is secreted by pancreatic islets in response to glucose, while IGF-1 and IGF-2 are primarily produced in the liver. Both IGFs are also expressed in various tissues and act via endocrine and paracrine mechanisms (*3*). These hormones exert their effects through receptor tyrosine kinases (TKs): the insulin receptor isoforms A (IR-A) and B (IR-B), and the IGF-1 receptor (IGF-1R) (*4*, *5*). Insulin binds with high affinity to IR-A and IR-B and IGF-1 to IGF-1R. In contrast, insulin binding to IGF-1R and IGF-1 binding to IRs is markedly weaker. IGF-2 is more promiscuous, interacting strongly with both IR-A and IGF-1R, although with lower affinity than insulin and IGF-1 (*5*). Because of their structural and sequence similarity, IR-A/B and IGF-1R can form hybrid receptors comprising one IR and one IGF-1R protomer (*6*). These hybrids are prevalent in tissues coexpressing both receptors (*7*) and are activated predominantly by IGF-1 (*8*). In addition, IGF-2 can bind with high affinity to the cation-independent mannose-6-phosphate receptor (CI-M6PR/IGF-2R), which also mediates lysosomal trafficking of mannose-6–phosphorylated proteins (*9*).

Binding of insulin and IGFs to IR-A, IR-B, or IGF-1R activates the receptors via TK phosphorylation, triggering intracellular signaling cascades through sequential phosphorylation events. The signaling pathways triggered by insulin-like hormones differ in some respects but also show considerable overlap (*10*, *11*). Insulin primarily regulates energy homeostasis via IR-A and IR-B, whereas IGF-1 and IGF-2 act through IGF-1R and IR-A to stimulate mitogenic processes related to growth and repair. During development, the coordinated action of all three hormones and their receptors is essential for fetal growth and organogenesis (*2*, *12*). The brain is now recognized as an important target of the action of both insulin and IGFs. These hormones synergistically regulate neuronal proliferation, survival, and differentiation, and modulate hypothalamic control of systemic metabolism, feeding, and energy balance. They also influence synaptic plasticity, neurotransmission, and the formation of neuronal circuits, which underpin learning, memory, and cognition throughout life (*13*–*18*).

In this study, we aimed to determine whether analogs of insulin, IGF-1, or IGF-2 with high potency at multiple receptors could more effectively promote cell growth and protection—particularly in neuronal cells—than their native counterparts. In our previous and ongoing research, we developed hormone analogs with modified affinities for IR-A, IR-B, and IGF-1R, as summarized in Table 1. Here, we investigated their signaling properties and mitogenic activity across several cell models. We designed an insulin analog, **1**_Ins_ ([GluB10, D-HisB24, GlyB31, TyrB32]-insulin), that combines strong binding to all three receptors, including IGF-1R at levels approaching that of native IGF-1. This dual-receptor engagement translated into enhanced biological activity across cellular and animal models, supporting the therapeutic potential of the **1**_Ins_ analog in contexts requiring coordinated IR and IGF-1R signaling.

**Table 1.** Relative binding affinities of insulin and IGF-1 analogs for receptors. Relative binding affinities of insulin and IGF analogs for human IR-A (IM-9 cells), IR-B, IGF-1R (transfected fibroblasts), IGF-2R domain 11 (D11), and IGFBP-3. nb, no binding at 10^−6^ M. ^a^Relative affinities (%): IR-A/IR-B to insulin, IGF-1R to IGF-1, IGF-2R and IGFBP-3 to IGF-2, calculated as (*K*_d_ native hormone/*K*_d_ analog) × 100. *K*_d_ values are in table S1. ^b to e^ Data from (*20*–*22*, *24*), respectively.

		Binding affinities (%)^a^ for				
Code	Analog	IR-A	IR-B	IGF-1R	IGF-2R D11	IGFBP-3
	Insulin	100	100	0.08	nb	nb
	IGF-1	1	0.2	100	nb^e^	64^e^
	IGF-2	9	1	11	100^e^	100^e^
**1**_Ins_	[Glu^B10^, D-His^B24^ Gly^B31^, Tyr^B32^]-insulin	219	950	79	nb	nb
**2**_Ins_	[Ala^B29^, Glu^B31^, amide^B31^]-insulin	44^b^	140^b^	nb	nb	nb
**3**_Ins_	[Cyclo(Nva(δN_3_)^B26^-Prg^B29^)]-insulin	226^c^	515^c^	0.02^c^	nb	nb
**4**_IGF-1_	[His^49^]-IGF-1	5^d^	0.7^d^	68^d^	nb	6.7
**5**_IGF-2_	[His^48^]-IGF-2	59^d^	12^d^	18^d^	50	69

## RESULTS

### Production of the analogs

The analog **1**_Ins_ ([GluB10, D-HisB24, GlyB31, TyrB32]-insulin) was prepared by total chemical synthesis, as described and documented in the Supplementary Methods and fig. S1. It was generated by combining the previously published [D-HisB24, GlyB31, TyrB32]-insulin (*19*) with the HisB10Glu mutation. The analogs **2**_Ins_ ([AlaB29, GluB31, amideB31]-insulin) and **3**_Ins_ ([cyclo(Nva(δN_3_)B26–PrgB29)]-insulin) were prepared as described in Panikova *et al.* (*20*) and Vikova *et al.* (*21*), respectively. The IGF analogs **4**_IGF-1_ ([His^49^]-IGF-1) and **5**_IGF-2_ ([His^48^]-IGF-2) were prepared as described by Machackova *et al.* (*22*).

### Promiscuous analogs strongly bind IR-A, IR-B, and IGF-1R

The relative binding affinities of the analogs for IR-A, IR-B, IGF-1R, domain 11 (D11) of IGF-2R (D11 is the main IGF-2 binding site), and IGF-binding protein-3 (IGFBP-3) are summarized in Table 1. Binding constants and representative binding curves for the insulin analog **1**_Ins_ are shown in table S1 and fig. S2. Compared to native insulin, **1**_Ins_ shows a 2-fold higher affinity for IR-A, 9-fold for IR-B, and nearly 1000-fold for IGF-1R. Despite being an insulin analog, **1**_Ins_ binds IGF-1R more strongly than IGF-2 and approaches the potency of IGF-1, suggesting its dual insulin and IGF-1R agonism.

Binding affinities of analogs **2**_Ins_ to **5**_IGF-2_ were derived from previous studies (table S1). The insulin analog **2**_Ins_ (*20*) shows increased specificity for IR-B, reduced affinity for IR-A, and no IGF-1R binding. **3**_Ins_ (*21*) binds more strongly to both IR-A and IR-B, but less to IGF-1R. These analogs were included to distinguish insulin- and IGF-1–specific signaling in our experiments.

IGF analogs **4**_IGF-1_ and **5**_IGF-2_ (*22*) show ~5-fold higher binding affinity for IR-A and 3- to 12-fold higher affinity for IR-B compared to native IGFs, with minimal change in IGF-1R binding (Table 1). Like **1**_Ins_, they were included to test whether preserved IGF-1R binding combined with enhanced IR-A/IR-B binding could produce distinct effects and reveal processes requiring activity across all three receptors.

We assessed analog binding to IGF-2R D11 (*23*) and IGFBP-3 (*24*), which are key regulators of IGF-2 and of both IGF-1 and IGF-2 activities, respectively (Table 1, table S1, and figs. S3 and S4). As expected, insulin analogs showed no binding to IGF-2R or IGFBP-3. **4**_IGF-1_ bound IGFBP-3 but not IGF-2R. In contrast, **5**_IGF-2_ showed ~twofold reduced IGF-2R binding versus IGF-2, with near-identical IGFBP-3 affinity.

### Cryo-EM structures of **1**_Ins_ bound to IR-A and IGF-1R reveal the structural basis for dual receptor affinity

**Fig. 1.**
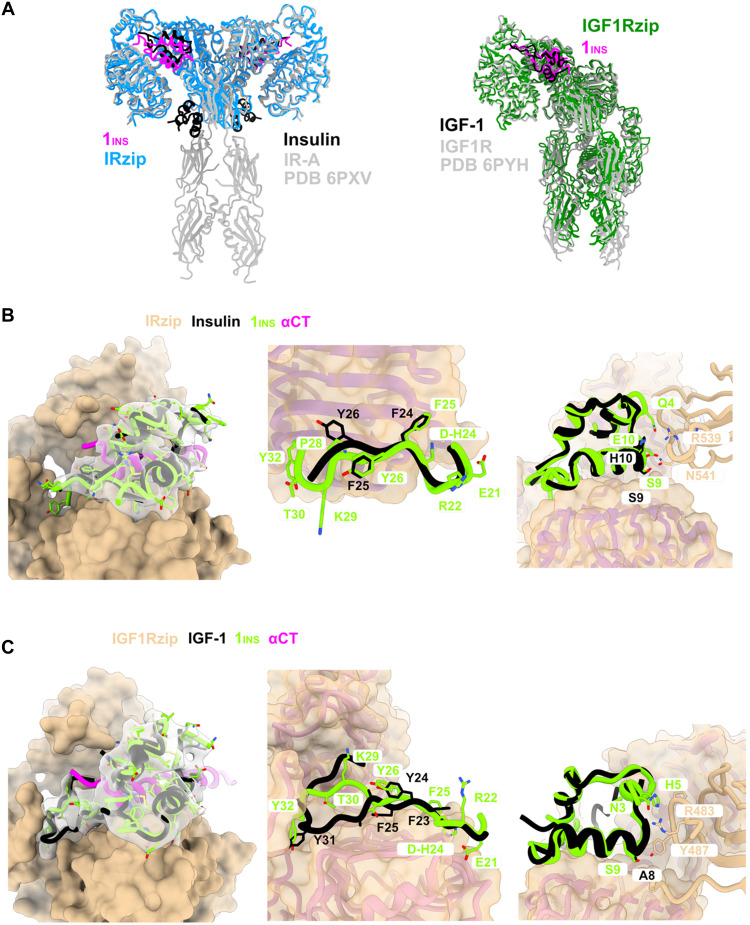
Cryo-EM structures of 1_Ins_ bound to IR-A and IGF-1R. Comparison with insulin (PDB: 6PXV) and IGF-1 (PDB: 6PYH) complexes. (**A**) Overall cryo-EM models: **1**_Ins_-IRzip (magenta/blue) aligned to insulin-IR (black/gray); **1**_Ins_–IGF-1R (magenta/green) aligned to IGF-1–IGF-1R (black/gray). [(**B**) left] **1**_Ins_ (green) with cryo-EM density aligned to native insulin (black); IR C-terminal peptide of α-subunit (αCT) in magenta. [(B) center/right] Down-shift and GlyB31-TyrB32 motifs and FnIII-1′ interactions of **1**_Ins_ aligned to insulin. [(**C**) left] **1**_Ins_ (green) aligned to IGF-1 (black); IGF-1R αCT in magenta. [(C) center/right] Down-shift and GlyB31-TyrB32 motifs and FnIII-1′ interactions of **1**_Ins_ aligned to IGF-1. Residues are labeled in single-letter code, with **1**_Ins_ numbering in green and insulin/IGF-1 in black. Receptor surface is shown in coral, ribbons in pink.

To uncover how **1**_Ins_ engages both insulin and IGF-1Rs, we analyzed cryo–electron microscopy (cryo-EM) structures of its complexes with IR-A and IGF-1R. **1**_Ins_-saturated complexes of GCN4 leucine zipper fusion constructs IRzip and IGF1Rzip were generated immediately before vitrification (4:1, **1**_Ins_ to receptor monomer). High-resolution cryo-EM datasets were collected on a Titan Krios G4. Single-particle analysis resulted in structures of the complete ectodomain for IGF-1R with one copy of **1**_Ins_ (3.3 Å, C1 symmetry) and the “head” of IR with two copies of **1**_Ins_ (2.8 Å, C2 symmetry, leucine-rich repeat domain 1 (L1), cysteine-rich domain (CR), leucine-rich repeat domain 2 (L2)] and fibronectin type III domain 1 (FnIII-1) from both monomers (figs. S5 and S6 and table S2). Some orientation bias was observed in both datasets, resulting in anisotropic but high-resolution maps sufficient for atomic modeling from previous structures for each. Overall, both receptors adopted active conformations essentially identical to the active receptors with their cognate ligands [e.g., Protein Data Bank (PDB):6PXV, PDB:6PYH, and PDB:6VWG/6VWH] (Fig. 1A) (*25*–*27*).

**1**_Ins_ was observed in a similar conformation in both structures, demonstrating an insulin-like fold, with no change in secondary structure. The B-chain C-terminal tails were observed in partially ordered density in both structures and differed in conformation between the two structures (Fig. 1B).

In the IR-A structure, the GlyB31-TyrB32 motif was observed on the solvent-exposed lateral surface of L1, forming H bonds between **1**_Ins_ TyrB32 and IR-GluB22 side chains, and between IR-A Arg^19^ and the **1**_Ins_ GlyB31 backbone carbonyl (Fig. 1B). The “down-shift”–inducing residue D-HisB24 [reported previously by Zakova *et al.* (*28*) and Chrudinova *et al.* (*19*)] was observed in a pocket between L1 Asn^15^ and Lys^40^. **1**_Ins_ PheB25 replaced insulin PheB24, TyrB26 replaced PheB25 and the TyrB26 pocket was left empty. The modification of B10 from His to Glu permits an additional hydrogen bond to Asn541 (Fig. 1B).

In the case of IGF-1R, **1**_Ins_ residues GlyB31-TyrB32 were observed in the equivalent position to Gly30-Tyr31 in IGF-1, taking a more direct path across L1 to reach the pocket (Fig. 1C). The down-shift–inducing residue D-HisB24 was observed to flip away the Asn^11^ side chain, positioned in the place of Gly^22^ from IGF-1, and did not appear to form any meaningful interactions with IGF-1R. **1**_Ins_ TyrB26 is down-shifted to the position of IGF-1’s Tyr^24^. **1**_Ins_ AsnB3 and GluB10 form an H bond network with Arg^483^ to stabilize the ligand at site 1b FnIII-1′. Analog’s SerB9 forms an additional H bond with IGF-1RTyr^487^ (Fig. 1C). The comparison of primary sequences and main structural features of receptor-bound insulin, **1**_Ins_ and IGF-1 is shown in fig. S7.

### Model neuronal cells predominantly express IGF-1R and form hybrids with IR-A

Next, we examined the potential benefits of synergistic insulin and IGF-1R activation, focusing on neuroprotection. We used several model systems (see Materials and Methods): mouse fibroblasts from IGF-1R–knockout mice stably expressing human IR-A, IR-B, or IGF-1R; human SH-SY5Y neuroblastoma and U87MG glioblastoma cell lines; and primary rat neurons—either neonatal primary cultures (mixed cortical/hippocampal) or embryonic cortical cultures.

Because multiple cell models were used, we quantified receptor expression to assess IR-A, IR-B, and IGF-1R levels (tables S3 and S4). mRNA levels were measured by quantitative reverse transcription polymerase chain reaction (qRT-PCR) and correlated with protein levels via Western blot (fig. S8). Band intensities reflected the corresponding mRNA expression.

Mouse fibroblasts expressed low levels of endogenous IR-A, but expression of transfected human receptors was much higher, excluding any contribution from mouse IR-A. IGF-1R was the predominant receptor in both SH-SY5Y and U87MG cell lines. However, total receptor levels were much lower in U87MG cells, which accordingly showed a weaker response to hormone treatment.

Because of limited material, mRNA analysis was not performed in primary neuronal culture. However, Western blots (fig. S8) confirmed expression of both IR-A and IGF-1R. In adult mouse frontal cortex, IGF-1R was also the predominant receptor (table S3).

In cells expressing both receptors, IR-A and IGF-1R subunits are expected to form hybrid receptors. On the basis of Siddle’s random assembly model (*29*), we roughly estimated receptor distributions in the tested cell lines (table S3). On the basis of our estimations; transfected fibroblasts predominantly contain receptor homodimers. U87MG cells express few IGF-1Rs and negligible IGF-1R/IR-A or IR-B hybrids. SH-SY5Y cells show typical receptor levels, with negligible IR-A homodimers and an estimated IGF-1R to IGF-1R/IR-A hybrid ratio of 4:1. Rat primary neuronal culture express comparable amounts of IGF-1R and IR-A but include diverse cell types (*30*) that cannot be distinguished. In mouse forebrain, IR homodimers represent ~10% of receptors, and the IGF-1R to IGF-1R/IR hybrid ratio is estimated at ~1:1. Again, receptor distributions may vary across cell types.

We performed additional experiments using two other cell models and the specific IR inhibitor S661 (*31*, *32*), and the results further support the assumption that hybrid receptors may influence hormone signaling. Detailed results and their discussion are provided in the Supplementary Materials (contribution of individual receptors to the dual receptor activity of **1**_Ins_; table S5 and figs. S9, S10, and S11).

### Promiscuous analogs induce stronger insulin and IGF-1 signaling than native hormones

We assessed concentration-dependent receptor autophosphorylation in stably transfected fibroblasts. Activation curves for the analogs are shown in fig. S12 and largely mirror their binding affinities.

**Fig. 2.**
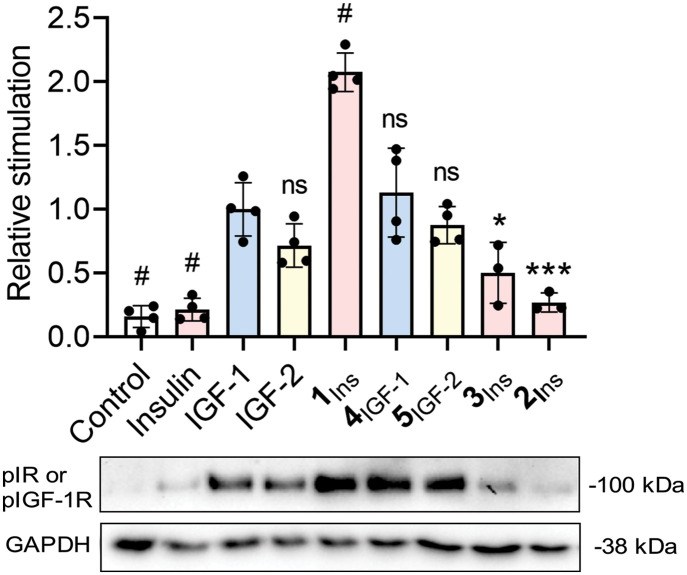
Relative abilities of hormones and analogs to stimulate receptor autophosphorylation in rat primary neuronal culture. Cells were stimulated with 10 nM ligands for 20 min; phosphorylation of IR/IGF-1R was assessed by Western blot. Data were normalized to GAPDH (glyceraldehyde-3-phosphate dehydrogenase) loading control and are shown relative to IGF-1–induced signal. The GAPDH loading control is the same as in Fig. 3 (D and H) because the respective samples were analyzed on the same gel. We show a representative blot, and all blots are in the Supplementary Materials. Bars: control (white), insulin analogs (light red), IGF-1 analogs (light blue), and IGF-2 analogs (light yellow). Asterisks indicate significant differences from IGF-1 (*P < 0.05; ***P < 0.001; # < 0.0001; ns, not significant according to ANOVA).

Receptor activation at 10 nM ligand was compared in transfected fibroblasts (fig. S13) and primary rat neuronal culture (Fig. 2). In all fibroblast lines overexpressing IR-A, IR-B, or IGF-1R, the promiscuous analogs **1**_Ins_, **4**_IGF-1_, and **5**_IGF-2_ were as potent or more effective than their native counterparts. Notably, **1**_Ins_ activated IGF-1R as efficiently as native IGF-1 (fig. S13C), and in primary neuronal cultures, **1**_Ins_ induced significantly stronger activation than insulin, IGF-1, or IGF-2 (Fig. 2). In our standard experimental setting, autophosphorylation signals from SH-SY5Y and U87MG cells were too weak for quantification due to low receptor content. The blots showing the signals of total proteins (Akt, Erk, IR-A, and IGF-1R), including both phosphorylated and nonphosphorylated forms, are shown in fig. S14.

**Fig. 3.**
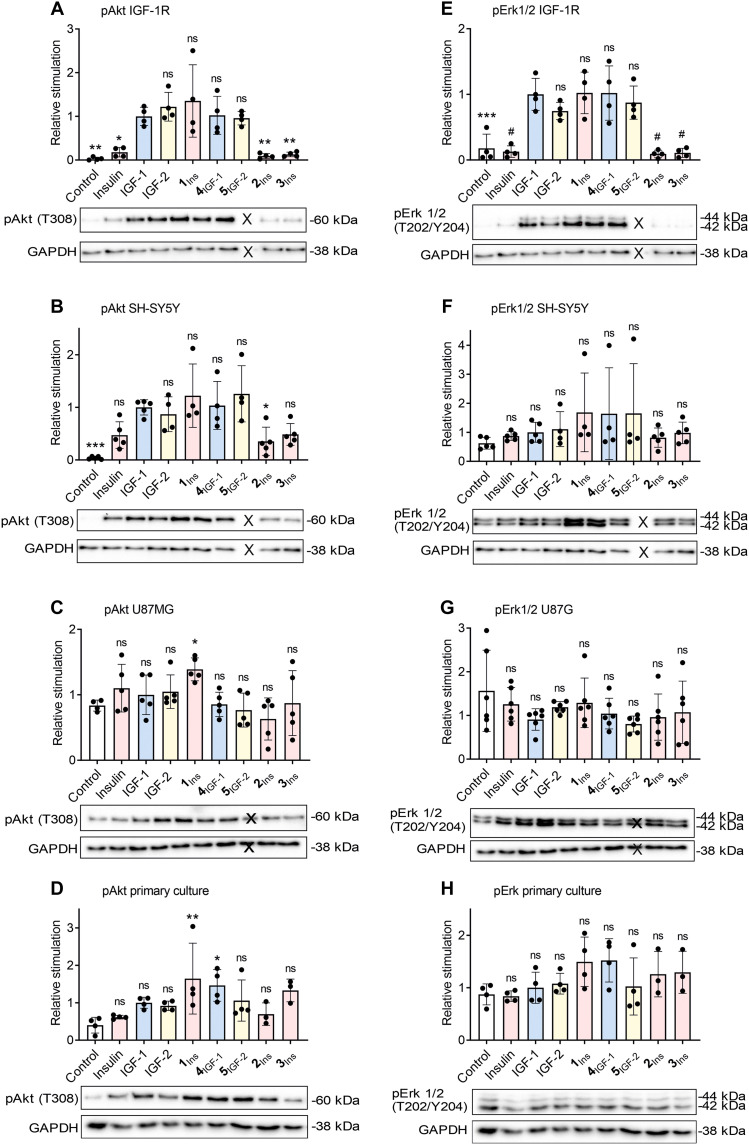
Relative abilities of hormones and analogs to stimulate Akt and Erk1/2 signaling. Cells expressing IGF-1R (**A** and **E**), SH-SY5Y (**B** and **F**), U87MG (**C** and **G**), and rat primary neuronal culture (**D** and **H**) were stimulated with 10 nM ligands for 20 min. Phosphorylation of Akt (A to D) and Erk1/2 (E to H) was assessed by Western blot. Data were normalized to GAPDH loading control and are shown relative to IGF-1–induced signal. Identical GAPDH loading controls are shown for the figure pairs (A) and (E), (B) and (F), and (D) and (H) because the samples in each pair were run on the same gel. Representative blots are shown and all blots are shown in the Supplementary Materials. Color coding as in Fig. 2. Asterisks indicate significance versus IGF-1 [(A), (B), and (E)] or versus control [(C), (D), (F), (G), and (H)]. (*P < 0.05; **P < 0.01; ***P < 0.001; #*P* < 0.0001; ns, not significant according to ANOVA). X indicates that this sample was not included in the study.

Figure 3 shows Akt and Erk activation by analogs and native hormones. Neuronal cell models were compared to IGF-1R–transfected fibroblasts, where IGF-1R was the dominant receptor. U87MG cells responded only moderately, likely because of low receptor expression, and Akt phosphorylation was not extinguished by serum starvation. As expected, IGF-1 stimulated signaling more strongly than insulin in the other cell models. In IGF-1R–transfected cells, downstream kinases were robustly activated by IGF-1, IGF-2, and the promiscuous analogs **1**_Ins_, **4**_IGF-1_, and **5**_IGF-2_. In contrast, nontransfected lines showed no significant pErk1/2 increase, while pAkt was clearly modulated. In SH-SY5Y and primary neuronal culture, the promiscuous analogs showed strong pAkt stimulation with a trend to surpass IGF-1. In the primary culture, **1**_Ins_ and **4**_IGF-1_ induced a significantly stronger signal than IGF-1 (Fig. 3).

We further examined the time course of IGF-1R, Akt, and Erk activation in SH-SY5Y cells after stimulation with insulin, IGF-1, and **1**_Ins_. The results are shown in fig. S15. There was no marked difference in the activation profile among the ligands; however, **1**_Ins_ slightly outperformed IGF-1 at all time points. The total level of IGF-1R remained unchanged.

### Promiscuous analogs promote cell viability and resistance to MG in neuronal cells

**Fig. 4.**
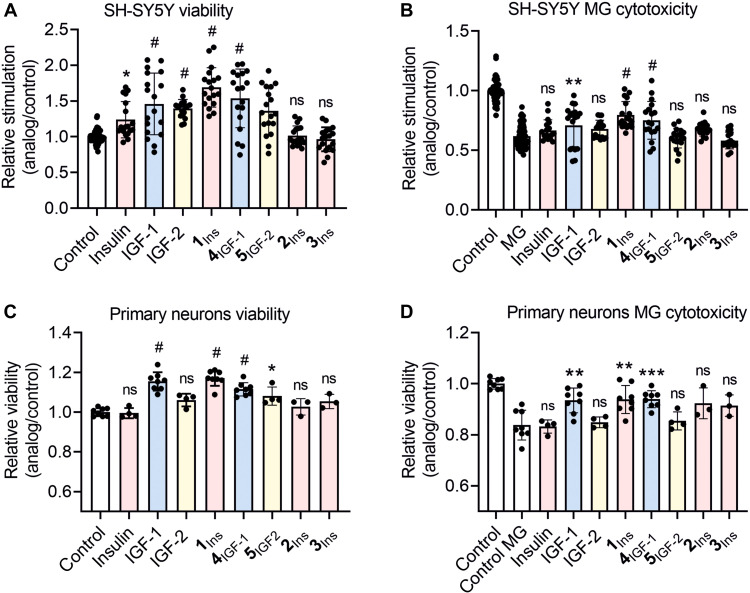
Effects of hormones and analogs on neuronal cell viability and MG-induced toxicity. SH-SY5Y cells (**A** and **B**) and rat primary cortical neurons (**C** and **D**) were treated with 10 nM insulin, IGFs, or analogs. Proliferation was measured after 20 hours (SH-SY5Y) or 48 hours (primary neurons); rescue was assessed after 16 hours of cotreatment with 1.5 mM (SH-SY5Y) or 0.6 mM (neurons) MG. MTT assay results were normalized to untreated (viability) or MG-treated (rescue) controls. Color scheme as in Fig. 2. Asterisks denote significance (*P < 0.05; **P < 0.01; ***P < 0.001; #*P* < 0.0001; ns, not significant according to ANOVA).

We assessed the cell viability of SH-SY5Y and rat cortical neurons treated with the analogs using the MTT [3-(4,5-dimethylthiazol-2-yl)-2,5-diphenyltetrazolium bromide] assay. Cell viability after 10 nM hormone treatment is shown in Fig. 4; effects of 1 and 100 nM in SH-SY5Y cells are in fig. S16. SH-SY5Y cells were treated for 20 hours and primary neurons for 2 days.

Methylglyoxal (MG) is a toxic byproduct of glycolysis that contributes to oxidative damage in diabetes, including nerve cell degeneration (*33*). Hence, we also evaluated the ability of 10 nM (Fig. 4) and also 1 and 100 nM (fig. S16) hormones to rescue SH-SY5Y cells viability after 16 hours of exposure to MG.

The combined effect of insulin and IGF-1 was also assessed (fig. S16, C and D). Costimulation with insulin and IGF-1 (10 nM each) produced a similar effect to treatment with 10 nM **1**_Ins_. However, at 1 nM, **1**_Ins_ was significantly more potent.

Promiscuous analogs **1**_Ins_ and **4**_IGF-1_, along with native IGF-1, promoted survival in both cell types. Notably, **1**_Ins_ outperformed IGF-1 although the effect was not significant with the exception of 1 nM treatment (fig. S16). IGF-2 and **5**_IGF-2_ were less effective, while insulin and its analogs **2**_Ins_ and **3**_Ins_ showed minimal effects.

To discern involvement of proliferative characteristics of the hormones on cell viability, we performed proliferation assay on SH-SY5Y cells measuring 5-ethynyl-2′-deoxyuridine (EdU) incorporated into the newly synthesized DNA. The treatment with the hormones had strong proliferative effect (fig. S17), suggesting that the observed increased cell viability was related to cell proliferation.

### Promiscuous analogs **1**_Ins_ and **4**_IGF-1_ slow down MG-induced autophagy flux in SH-SY5Y cells

MG is a precursor of advanced glycation end products that induce intracellular reactive oxygen species (ROS), leading to apoptosis. Autophagy is a cellular stress–response mechanism that alleviates cell death under various stimuli, including ROS. MG has been shown to increase autophagy as a protective mechanism against cell death (*34*). In contrast, insulin and IGF-1 strongly suppress autophagy in astrocytes (*35*). We examined whether hormone-mediated protection against MG toxicity involved autophagy. SH-SY5Y cells were treated with 0.5 mM MG for 2 hours; higher concentrations or longer exposure caused detachment, so we focused on early events. We analyzed SQSTM and LC3 A/B autophagy markers (fig. S18). Although SQSTM signal was weak and not quantifiable, MG treatment reduced its band intensity. MG also induced conversion of LC3-I to the autophagosome-associated LC3-II showing that autophagy was induced by the MG treatment. However, all hormones and analogs prevented the conversion of LC3-I to LC3-II in both untreated and MG-treated cells. Promiscuous analogs **1**_Ins_ and **4**_IGF-1_ were more effective than other variants in suppressing MG-induced autophagy. We suggest that the principal mechanism underlying the hormones’ effects on cell viability is the activation of the prosurvival insulin/IGF-1 signaling pathway.

### **1**_Ins_ effectively modulates glucose metabolism in mice (ITT) and (hyperinsulinemic-euglycemic clamp)

**Fig. 5.**
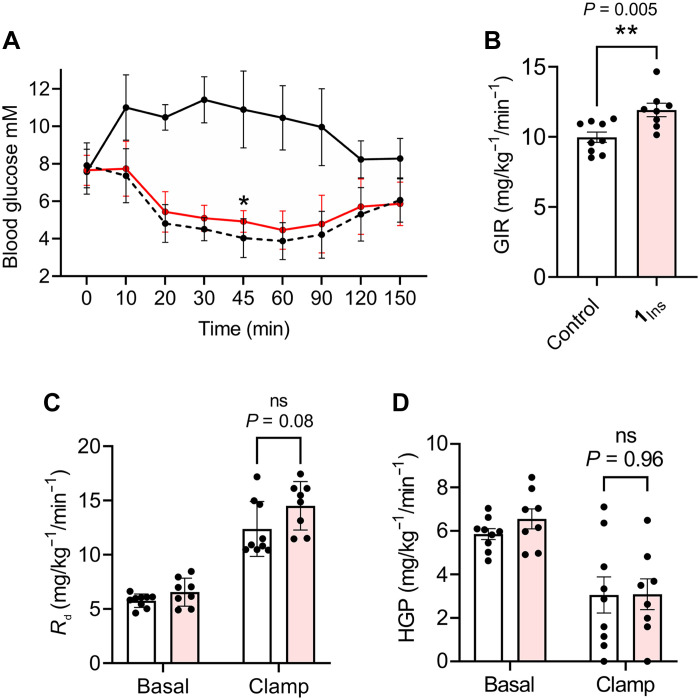
Metabolic activity of 1_Ins_ in vivo. (**A**) ITT in mice (*n* = 10). Effect of saline (control) is shown as a solid line in black, effect of human insulin (0.75 U/kg) as a dashed line in black, and effect of the analog **1**_Ins_ (0.75 U/kg) as a solid line in red. The asterisk indicates significant difference between the effect of the analog and human insulin (*P < 0.05, *t* test). (**B**) GIR in euglycemic clamp (*n* = 8, **P < 0.005, *t* test). (**C**) Glucose disposal (*R*_d_, *n* = 8, *t* test) and (**D**) HPG (*n* = 8, *t* test) under basal and clamp conditions after administration of native insulin (control, in white) or **1**_Ins_ (in light red) in rats. Experimental details are provided in Materials and Methods.

To test whether the promiscuous analog **1**_Ins_ retains insulin-like metabolic activity despite its high IGF-1R potency, we compared its ability to lower blood glucose with that of native insulin using the insulin tolerance test (ITT) in mice. At 0.75 U/kg, **1**_Ins_ was as effective as insulin (Fig. 5A).

Next, we evaluated the comparative effect of normal insulin under hyperinsulinemic conditions versus an equal dose of infused **1**_Ins_ on overall insulin responsiveness. When assessing effects on overall insulin sensitivity, **1**_Ins_ resulted in a significantly greater glucose infusion rate (GIR) than normal insulin during a 3-mU clamp (Fig. 5B, *P* = 0.005). To surmise the potential contribution of glucose fluxes to this response, we further evaluated effects on glucose disposal (*R*_d_) and hepatic glucose production (HGP). *R*_d_ was increased during the clamp phase, as compared to basal in both groups, but differences in regular insulin versus **1**_Ins_ did not reach significance (Fig. 5C, *P* = 0.08). HGP was similarly suppressed by both regular insulin and **1**_Ins_ (Fig. 5D, *P* = 0.96).

### Phosphoproteomics reveals that **1**_Ins_ mimics both insulin and IGF-1 signaling

We performed global phosphoproteomic analysis in SH-SY5Y cells to compare downstream signaling induced by insulin, IGF-1, and **1**_Ins_. The SH-SY5Y cells were chosen because they represent a widely used model of nondifferentiated neuronal cells (*36*). We aimed to investigate the signaling of our new analog with dual receptor activation in the context of potential neuroregeneration, presumably driven by neuronal stem cells and progenitors.

A total of 9168 phosphosites were quantified. Among regulated sites (false discovery rate, FDR < 0.15), 295 were altered by **1**_Ins_, 175 by IGF-1, and 83 by insulin. Many brain-specific phosphoproteins were detected. Phosphoproteomic data on Akt and Erk1/2 activation were consistent with Western blot results (Fig. 3 and table S6). For example, Thr-phosphorylated Akt3 (Q9Y234_T305) increased 19-fold with **1**_Ins_, 18-fold with IGF-1, and 7-fold with insulin (Perseus analysis). Western blotting showed corresponding increases of 30-, 25-, and 11-fold. For Erk1/2, phosphopeptides showed 2 to 6× induction by **1**_Ins_, 2 to 4× by IGF-1, and 2 to 3× by insulin, aligning with Western blot data (2.5×, 1.5×, and 1.2×, respectively). Notably, Western blot signals showed high variability.

**Fig. 6.**
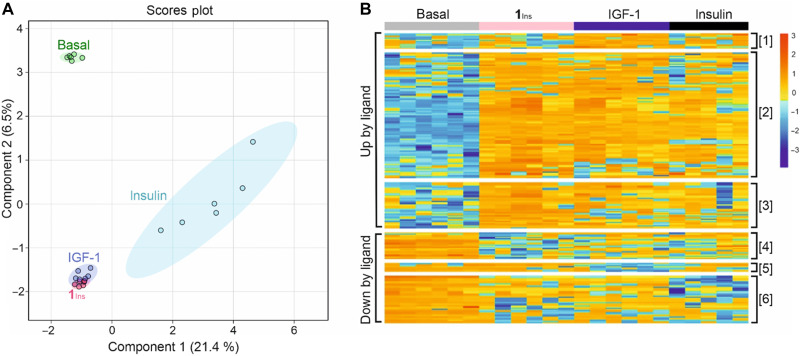
Phosphoproteomic profiles of SH-SY5Y cells stimulated with insulin, IGF-1, or 1_Ins_. (**A**) Sparse PLS-DA showing separation of phosphoproteomes: basal (green), insulin (blue), IGF-1 (violet), and **1**_Ins_ (orange). (**B**) Hierarchical clustering heatmap of phosphosites significantly regulated fourfold with FDR < 0.15 by at least one ligand. Data are shown as *z* scores of log_2_-transformed intensities. Clusters [1] to [3]: up-regulated phosphosites (patterns: [1] IGF-1 > **1**_Ins_ ≈ insulin; [2] IGF-1 ≈ **1**_Ins_ > insulin; [3] **1**_Ins_ > IGF-1 ≈ insulin). Clusters [4] to [6]: down-regulated phosphosites ([4] **1**_Ins_ > IGF-1 ≈ insulin; [5] IGF-1 > **1**_Ins_ ≈ insulin; [6] **1**_Ins_ ≈ insulin > IGF-1).

Sparse partial least squares discriminant analysis (sPLS-DA) showed clear separation between basal and ligand-induced phosphoproteomes, with distinct clustering for each ligand (Fig. 6A). IGF-1– and **1**_Ins_-induced profiles largely overlapped and were clearly distinct from those of insulin or unstimulated cells. This aligns with the predominance of IGF-1R in SH-SY5Y cells and low IR-A expression, likely present mainly as hybrid receptors with limited insulin sensitivity.

Phosphosites significantly altered with ≥ fourfold change (FDR < 0.15) by at least one ligand were analyzed by hierarchical clustering. The resulting heatmap (Fig. 6B) revealed six clusters: clusters [1] to [3] comprised up-regulated, and clusters [4] to [6] down-regulated sites. The largest, cluster [2], contained phosphosites up-regulated by all three ligands—core components of insulin/IGF-1 signaling in SH-SY5Y cells—with varying response strength. Many were coregulated by **1**_Ins_ and IGF-1, but not insulin. In addition, IGF-1-specific (cluster [1]) and, notably, **1**_Ins_-specific (cluster [3]) phosphosites were identified.

We analyzed Gene Ontology (GO) terms for each cluster using the UniProt database (fig. S19). Clusters [2] and [3] shared GO terms related to insulin/IGF-1 signaling, cell proliferation, and apoptosis regulation. Cluster [1], preferentially regulated by IGF-1, was enriched in chromatin remodeling, transcriptional control, and stress response.

Clusters [4] to [6], which contained down-regulated phosphosites, showed distinct profiles. As with up-regulated clusters, specific sites were preferentially down-regulated by individual ligands. Overall, GO terms for these clusters involved transcription, actin remodeling, and cellular stress response.

**Fig. 7.**
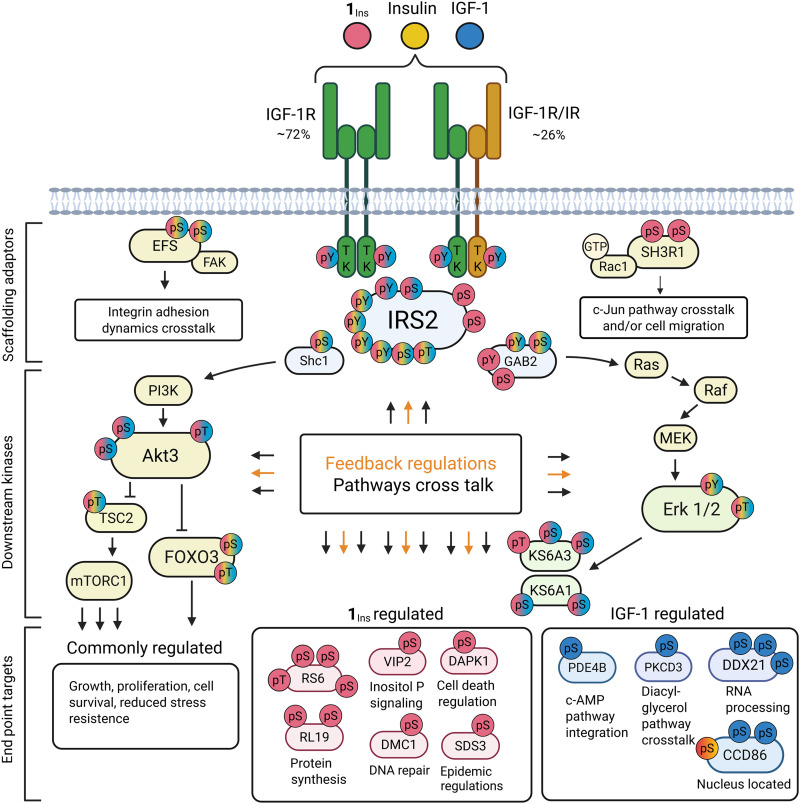
Key signaling features in SH-SY5Y cells stimulated by 1_Ins_, IGF-1, or insulin. Schematic overview of IR-A/IGF-1R expression in SH-SY5Y cells and downstream Akt/MAPK pathways. Black lines indicate activation; orange lines denote feedback regulation. Colored circles represent significantly regulated phosphosites at 15 min: red (**1**_Ins_), blue (IGF-1), yellow (insulin); overlaps show coregulation. The specific numbering of phosphosites for individual proteins and qualitative information on their activation are provided in table S6. Abbreviations are as follows; for scaffolding adaptor proteins: SHC-transforming protein 1 (Shc1), insulin receptor substrate 2 (IRS2), GRB2-associated–binding protein 2 (GAB2), embryonal fyn–associated substrate (EFS), and E3 ubiquitin–protein ligase SH3RF1 (SH3R1); for downstream kinases: RAC-gamma S/T-protein kinase (Akt 3), mitogen-activated protein kinase 3 and 1 (Erk1 and 2), ribosomal protein S6 kinase alpha-3 and alpha-1 (KS6A3 and KS6A1), and factors tuberin (TSC2) and FOXO3. End point targets regulated by **1**_Ins_ are as follows: small ribosomal subunit protein eS6 (RS6), large ribosomal subunit protein eL19 (RL19), death-associated protein kinase (DAPK1), inositol hexakisphosphate and diphosphoinositol-pentakisphosphate kinase 2 (VIP2), sin3 histone deacetylase corepressor complex component SDS3 (SDS3), and mediator of DNA damage checkpoint protein 1 (MDC1). End point targets regulated by IGF-1 are PKCD3, 3′,5′-cyclic-AMP phosphodiesterase 4B (PDE4B), nucleolar RNA helicase 2 (DDX21), and coiled-coil domain–containing protein 86 (CCD86). Created in BioRender. Jiráček, J. (2026) https://BioRender.com/b5t02ee.

A simplified signaling map summarizing key features of **1**_Ins_, IGF-1, and insulin responses in SH-SY5Y cells is shown in Fig. 7. Volcano plots comparing stimulated and control cells are in fig. S20. While all ligands activate insulin/IGF-1 signaling, their effects differ quantitatively and qualitatively. For example, forkhead box protein O3 (FOXO3), tuberin (TSC2), and partially Erk1 were similarly regulated by all ligands, despite insulin’s low affinity for IGF-1R, which was the predominant receptor in SH-SY5Y. In contrast, phosphorylation of the IGF-1R/IR activation loop and Akt Thr^305^ was markedly stronger with **1**_Ins_ and IGF-1. Several phosphosites on adaptor proteins insulin receptor substrate 2 (IRS2) and GRB2-associated–binding protein 2 (GAB2) were differentially regulated. Notably, E3 ubiquitin-protein ligase SH3RF1 (SH3R1) phosphorylation was specific to **1**_Ins_. Terminal signaling targets also varied: e.g., small ribosomal subunit protein eS6 (RS6) or inositol hexakisphosphate and diphosphoinositol-pentakisphosphate kinase 2 (VIP2) were up-regulated by **1**_Ins_, while nucleolar RNA helicase 2 (DDX21) and 3′,5′-cyclic-AMP phosphodiesterase 4B (PDE4B) were IGF-1 specific. Full phosphoproteomics results and references are provided in the Supplementary Materials.

## DISCUSSION

In this study, we characterized insulin, IGF-1, and IGF-2 analogs with modified affinities for IR-A, IR-B, and IGF-1R. We aimed to determine whether promiscuous analogs with high potency at multiple receptors could promote cell growth and protection—especially in neuronal cells—more efficiently than the native hormones themselves.

Among the tested compounds, the insulin analog **1**_Ins_ ([GluB10, D-HisB24, GlyB31, TyrB32]-insulin) stood out for its ability to activate strongly both insulin receptors and IGF-1R. Introducing Glu at B10 into the previously reported [D-HisB24, GlyB31, TyrB32]-insulin (*19*) led to a dramatic increase in IGF-1R affinity, reaching 79% of IGF-1 potency. To date, **1**_Ins_ shows higher IGF-1R–binding affinity than other insulin analogs known for their strong IGF-1R binding, such as [AspB10]-insulin (X10 or Aspart B10; 0.47%), insulin glargine ([GlyA21, ArgB31, ArgB32]-insulin; 0.51%), [ArgB31, ArgB32]-insulin (1.6%) (*37*), and the parent [D-HisB24, GlyB31, TyrB32]-insulin (12%) (*19*).

Historically, insulin analogs with increased IGF-1R affinity such as [AspB10]-insulin (X10) and insulin glargine have been thoroughly investigated regarding their potential mitogenic or tumorigenic properties. AspB10 was shown to display sustained IR/IGF-1R signaling and enhanced tumor growth in vivo (*38*), whereas glargine and its metabolites behaved similarly to native insulin and did not show carcinogenic effects in chronic studies (*39*). Given its markedly higher IGF-1R affinity, **1**_Ins_ would be expected to induce strong mitogenic signaling as well, which we indeed observed in our cellular signaling, viability, and phosphoproteomic assays. Functionally, **1**_Ins_ thereby exhibits IGF-1–like mitogenic characteristics while retaining insulin-like metabolic activity, representing a unique dual receptor agonism. Such dual activity may be particularly relevant in tissues or pathophysiological contexts where both insulin and IGF-1 signaling are compromised (see below).

**1**_Ins_ integrates several structural features that act additively to enhance receptor binding. Cryo-EM complexes with IR-A and IGF-1R (Fig. 1) revealed that D-HisB24 induces the previously described down-shift of the B-chain C-terminal residues (*19*, *28*). This repositioning allows TyrB26 to occupy the B25 pocket and increases C-terminal flexibility, enabling GlyB31-TyrB32 to form new contacts, including TyrB32 interaction with the IGF-1R CR domain, which is critical for high IGF-1 specificity (*26*). Previous studies showed that GluB10-insulin stimulates lipogenesis more strongly than AspB10-insulin (*40*). In **1**_Ins_, the combination of D-HisB24, GlyB31-TyrB32, and GluB10 produces a strong synergistic effect, boosting IGF-1R affinity nearly 1000-fold over native insulin. GluB10 contacts IR-A–Asn^541^ and IGF-1R–Arg^483^, consistent with Glu’s role in IGF-1R (*26*) and IR (*41*) binding.

**1**_Ins_ shows pronounced IR-B/IR-A specificity (~4.3-fold versus native insulin), making it one of the most IR-B–specific insulin analogs reported (*42*). This preference stems from its exceptionally high affinity for IR-B (950% of native insulin; Table 1). A recent study by An *et al.* (*43*) revealed that, in *apo* IR-B, the extended αCT segment (due to exon 11) interacts with the FnIII-2 domain, stabilizing the inactive state and reducing αCT threading by ligands. This may hinder insulin binding but allow faster association of **1**_Ins_. While we did not solve the structure of IR-B bound to **1**_Ins_, it is reasonable to assume that it resembles our IR-A complex structurally, with enhanced IR-B affinity arising from favorable association kinetics involving the αCT–FnIII-2 interface.

IR and IGF-1R can form hybrid receptors, detected in many cell types, although their physiological roles remain debated (*44*). Although hybrid receptors were identified and studied in earlier studies, they have remained a somewhat overlooked aspect of insulin and IGF-1 biology, likely because their experimental investigation is technically more demanding and less straightforward than the study of the individual IR and IGF-1R. In this respect, our data on the inhibition of cell-line signaling by the peptidomimetic S661, a potent IR inhibitor (*31*) (Supplementary Materials, figs. S10 and S11), strongly suggest that this inhibitor is capable of blocking both the IR homodimer and the IR/IGF-1R hybrid receptors. This is an important finding that may facilitate future research on hybrid receptors.

As far as is currently known, hybrid receptors are preferentially—and perhaps even exclusively—activated by IGFs (*8*, *29*). However, hybrid receptors may trigger distinct signaling compared to homodimers (*10*). The structure of IGF-1 bound to a hybrid receptor was recently solved (*45*). IGF-1 engages the L1-CR-L2 module of the IGF-1R protomer and the αCT and FnIII-1 domains of the IR protomer, anchoring via Tyr^31^ into a CR pocket. Reduced insulin binding is likely due to weak interaction with IGF-1R L1-CR-L2 and the absence of a complete site 2. These structural constraints may be bypassed by **1**_Ins_, which can surpass insulin in hybrid receptor binding thanks to its unique binding characteristics.

The cellular models used in this study served two purposes: (i) to directly compare activation of IR and IGF-1R by hormone analogs in transfected fibroblasts, and (ii) to test whether the analogs stimulate growth and survival in neuronal and glial models—specifically SH-SY5Y, U87MG, and primary rodent neurons. These models may also offer insights into the neuroprotective potential of nonspecific insulin-like analogs in the central nervous system, such as via intranasal delivery, as shown for native hormones (*46*–*48*).

To interpret cellular responses, we first assessed receptor expression. qRT-PCR showed that SH-SY5Y and U87MG predominantly express IGF-1R, with lower IR-A levels, suggesting ~20% hybrid receptors. PCR could not be performed on primary cells because of limited material, but adult brain tissue showed higher IR-A expression, suggesting that hybrid receptors may comprise up to 40% of total receptors.

As expected, stably transfected fibroblasts showed receptor autophosphorylation consistent with the expressed receptor and applied ligand, with promiscuous analogs (**1**_Ins_, **4**_IGF-1_, and **5**_IGF-2_) outperforming native hormones (fig. S8). In primary neuronal culture (Fig. 2), **1**_Ins_ induced the strongest receptor activation, suggesting coexpression and simultaneous activation of IR and IGF-1R, as the anti-TK antibody detects both. Activation via IGF-1R/IR-A hybrid receptors—responsive preferably to IGFs—is also likely.

Dual agonist **1**_Ins_ consistently showed the strongest Akt activation across all neuronal models: SH-SY5Y, U87MG, and primary neuronal culture (Fig. 3). These results, along with weaker responses of the cells to native insulin, suggest that IGF-1R and IGF-1R/IR-A hybrids predominate.

Viability and rescue assay results (Fig. 4) aligned with insulin/IGF signaling activation patterns (Fig. 3). The relatively weaker effects of IGF-2 and **5**_IGF-2_ in rescue experiments may reflect scavenging by IGF-2R, abundantly expressed in all tested cell types, especially during the longer incubation (20 hours versus 20 min in signaling assays).

MG poisoning leads to the accumulation of advanced glycation end products and induces oxidative damage and apoptosis. Autophagy is a protective mechanism activated by cells to remove damaged proteins under stress. However, insulin and IGF-1 are strong suppressors of autophagy (*35*). Therefore, we examined the activity of our analogs with respect to these opposing processes. Hormone treatment, particularly with the promiscuous analogs **1**_Ins_ and **4**_IGF-1_, attenuated autophagic flux, suggesting that the proliferative signaling driven by these hormones outweighs other cellular mechanisms.

Analog **1**_Ins_ was as effective as native insulin in the ITT in mice, and its efficacy was confirmed in rats under hyperinsulinemic-euglycemic clamp conditions, where it produced a significantly higher GIR, increased glucose uptake (*R*_d_), and similar HGP. These data suggest that the GIR difference is mainly due to enhanced *R*_d_ rather than HGP suppression. The stronger *R*_d_ effect may reflect higher levels of hybrid receptors in skeletal muscle, where insulin signaling is less effective. Given **1**_Ins_’s high affinity for IGF-1R and hybrids, a weaker effect might be expected because of ligand scavenging, yet this is likely offset by its exceptionally strong IR binding, especially to IR-B. IGF-1, which signals mainly via IGF-1R, is markedly less effective in ITT (*49*), supporting this interpretation.

A key finding, consistent with binding and structural data, was that **1**_Ins_ activates the same phosphorylation pathways in SH-SY5Y cells as insulin and IGF-1 (summarized in Fig. 7). Notably, **1**_Ins_ also triggered phosphorylation of specific sites not affected by either native hormone. This supports its role as a true dual agonist, capable of coactivating both receptors and additionally modulating unique downstream targets. Such biased signaling—where noncognate ligands elicit distinct responses—is well documented (*50*, *51*). Proposed mechanisms include differences in affinity, binding kinetics, and receptor internalization (*52*). Our data suggest that ligand-specific receptor engagement can lead to differential phosphorylation of adaptor proteins, driving distinct downstream signaling. Our phosphoproteomics captured a single time point (15 min); dynamic differences between ligands may emerge over time. Insulin’s signaling profile may reflect its low affinity for IGF-1R and hybrid receptors (Table 1 and table S3), whereas **1**_Ins_ and IGF-1 share similar affinities but may differ in association/dissociation rates (*19*, *53*) or hybrid receptor engagement. A more detailed discussion of the phosphoproteomics data is provided in the Supplementary Materials.

Dual or even triple receptor agonism in drug design is a successful concept that has proven its usefulness, for example, in the treatment of obesity and type 2 diabetes using glucagon-like peptide-1 (GLP-1) receptor agonists (*54*, *55*). An excellent example of such an approach is the development of GLP-1–MK-80, a bimodal molecule that combines *N*-methyl D-aspartate receptor antagonism with GLP-1 receptor agonism to effectively reverse obesity, hyperglycemia, and dyslipidemia in rodent models (*56*). While GLP-1–MK-80 is a covalent conjugate of two ligands targeting two different receptors, **1**_Ins_ is a single hormone analog based on the insulin scaffold, carrying just four mutations that render it equipotent for IGF-1R and also enhance its affinity for both IR-A and IR-B. Our results clearly demonstrate the potential of protein engineering in the design of peptide hormones and show that it is possible to rationally construct small proteins that combine the functions of two distinct hormones with different physiological roles into a single molecule. The **1**_Ins_ analog may be particularly useful in scenarios where simultaneous activation of both insulin and IGF-1 pathways is desired (*13*–*18*), offering a more cost-effective and efficient therapeutic alternative to the combined use of both native hormones. Such an application could be especially relevant for central administration to the brain (*57*, *58*), aiming to improve cognitive function, alleviate symptoms of neurodegenerative diseases, or modulate peripheral metabolic balance (*59*, *60*). In addition, **1**_Ins_ could prove valuable in contexts such as the regulation of muscle stem cells for cultured meat production, where a concerted action of insulin and IGF-1 is required (*61*).

## MATERIALS AND METHODS

### Production of insulin and IGF analogs

Human insulin was a gift from F. Hubálek (Novo Nordisk A/S), human IGF-1 was provided by Tercica Inc., and human IGF-2 was purchased from GroPep Bioreagents. The insulin analog **1**_Ins_ ([GluB10, D-HisB24, GlyB31, TyrB32]-insulin) was prepared by total chemical synthesis, as described in detail in the Supplementary Materials. The previously published insulin analogs **2**_Ins_ ([AlaB29, GluB31, amideB31]-insulin) and **3**_Ins_ [cyclo(Nva(δN_3_)B26–PrgB29)]-insulin were prepared by enzymatic semisynthesis according to (*20*) and (*21*), respectively. We cloned, expressed, and purified the previously published IGF analogs **4**_IGF-1_ ([His49]-IGF-1) and **5**_IGF-2_ ([His48]-IGF-2) as described by Macháčková *et al.* (*22*).

### Cell cultures

Human IM-9 lymphocytes [American Type Culture Collection (ATCC), CCL-159], mouse embryonic fibroblasts derived from IGF-1R knockout mice (R^−^ cells) (*62*) stably transfected with human IR-A, IR-B, or IGF-1R (*63*) (provided by A. Belfiore, Catanzaro, Italy), were grown as described previously (*20*, *24*, *64*, *65*). The SH-SY5Y (ATCC, CRL-2266TM) neuroblastoma cell line was grown in Dulbecco’s modified Eagle’s medium supplemented with 10% fetal bovine serum (FBS), 1% nonessential amino acids, 1% streptomycin-penicillin, and 2 mM l-glutamine. The U87MG (ATCC, HTB-14) glioblastoma cell line was grown in minimum essential medium supplemented with 10% FBS, 1% streptomycin-penicillin, and 2 mM l-glutamine. All cell lines were cultivated at 37°C in a 5% CO_2_ humidified incubator.

### Primary rat neurons and adult brain tissues

We purchased Primary rat cortex neurons (Thermo Fisher Scientific, A10840-02). Cells were seeded at 96-well plate (30,000 cells per well) and grown according to the manufacturer’s protocol in Neurobasal Plus medium (Thermo Fisher Scientific, A3582901) supplemented with 2 mM l-glutamine and 2% B-27 serum–free supplement (Thermo Fisher Scientific, 17504044) on poly-d-lysine (0.1 mg/ml) and laminin (15 μg/ml) precoated plates. The cells were used for cell viability assay.

Postnatal cultures of neuronal cells were prepared according to the procedure described in (*30*). The cultures were prepared as a mixture of cortical and hippocampal cells from neonatal rat brains and contained about 60 to 70% neurons, and the rest were astrocytes. Animal procedures followed the European Communities Council Directive 86/609/EEC and were approved by the Institutional Ethical Committee on Animal Experimentation, Czech Academy of Sciences, Prague (protocols no.121-2023 and no.103-2024). Pregnant Wistar rats were obtained from Charles River. Day-1 newborn rats of both sexes were decapitated, and cortices with hippocampi were dissected. The cells were suspended in Neurobasal-A medium supplemented with 2% B27 Serum–free supplement and 0.5 mM GlutaMax-I and seeded to 0.07% polyethyleneimine and laminin (5 μg/ml) precoated plates in a density 3.5 × 10^5^ cells/ml. The cells were let to recover for 5 days with one medium exchange. The cells were used for signaling assay.

The mouse forebrains were harvested from 7- to 8-week-old male C57BL/6N male mice (Charles River Laboratories, Germany). Lysates were used for RNA isolation.

### Analyses of IR and IGF-1R expression

The amount of IR-A, IR-B, and IGF-1R mRNA expressions were assessed using standard RT-qPCR procedures (*66*). Used primers are listed in table S4.

Briefly, for RNA isolation, cells were lysed in TRI Reagent (Sigma-Aldrich). Minimum three different passages of cells were used. Tissues were homogenized in the same reagent using Tissue Lyser II (Qiagen). Samples were incubated for 5 min at room temperature (RT), centrifuged (12,000*g*, 10 min, 4°C), and the supernatant was transferred to a new tube. Chloroform in ratio 1:5 was added, and the samples were vortexed for 15 s, incubated for 5 min at RT, and centrifuged (12,000*g*, 15 min, 4°C). The aqueous phase was transferred to new tubes, 100% ethanol in ratio 1:1 was added, and subsequently, the Direct-zol RNA Miniprep Kit (Zymo Research) was used according to the manufacturer’s instructions. For subsequent cDNA synthesis, LunaScript RT SuperMix Kit (New England Biolabs) was used according to the manufacturer’s instructions. The qPCR was performed using LightCycler 480 SYBR Green I Master (Roche) according to the manufacturer’s instructions on LightCycler 480 II (Roche).

The receptor protein levels were studied using Western blots. Cells (5 × 10^5^) were lysed in 50 μl of lysis buffer [7 M urea, 2 M thiourea, 4% (w/v) CHAPS, 40 mM tris, 65 mM dithiothreitol (DTT), and 2% (v/v) ampholytes (pH 9 to 11)]. The protein content was determined using the Bradford assay. Proteins (10 μg) were applied on gels, and the blots were developed with anti–insulin receptor β (4B8) (Cell Signaling Technology, catalog no. 3025) or anti–IGF-1R β (111A9) antibody (Cell Signaling Technology, catalog no. 3018). Anti-actin (20-33) antibody (Sigma-Aldrich, catalog no. A5060) was used as a loading control.

### Cell viability assay

Cell viability was assessed using MTT test as described in (*67*). The SH-SY5Y cells were grown in 96-well plates (4 × 10^4^ cells per well) and starved overnight in serum-free medium. The wild-type ligands and analogs were added to the serum-free media at 1, 10, and 100 nM concentrations and cultivated for 20 hours. Combined action of insulin/IGF-1 was tested at 1, 5, and 10 nM each. Then, the cells were treated with MTT reagent (0.5 mg/ml) for 2 hours at 37°C. Living cells converted soluble MTT to insoluble formazan, which was subsequently dissolved in dimethyl sulfoxide. Measured absorbance at 570 nm was proportional to the number of living cells. For determination of the MG cytotoxic effect, 1.5 mM MG was added for 16 hours to the cells that were already treated for 4 hours with the analogs. The assay was repeated at least three times with six replicates of each treatment.

Similar assay was performed with Primary rat cortex neurons (Thermo Fisher Scientific, A10840-02). Cells were seeded at 96-well plate and grown according to the manufacturer’s protocol. After 4 days in culture medium, the supplement was replaced with B27 supplement minus insulin (Thermo Fisher Scientific, A1895601), and wild-type hormones and analogs were added at 10 nM concentration. The cells were grown for another 48 hours, and medium with hormones was replaced after 24 hours. For determination of the MG cytotoxic effect, 0.6 mM MG was added for 16 hours. The assay was repeated two times with four replicates of each treatment.

The data were related to the value measured in control cells. Means ± SD (*n* ≥ 4) values were calculated. The significance of the changes in cell viability was calculated by GraphPad Prizm 8 software, using one-way analysis of variance (ANOVA) with Dunnett’s test comparing all analogs versus control (untreated cells or MG-treated cells in the cytotoxicity assay).

### Cell proliferation assay

Cell proliferation was assessed using a modified Click-iT Plus EdU Alexa Fluor 488 Flow Cytometry Assay Kit (Invitrogen, catalog no. C10633). SH-SY5Y cells were plated at an initial density of 50,000 cells per well in a 24-well plate and synchronized by 24-hour serum starvation. Subsequently, the cells were treated for 24 hours with test compounds (10 nM **1**_Ins_, insulin, or IGF-1) or controls (untreated and full-media control). Proliferating cells were labeled during the final 4 hours of the treatment by adding 5 μM EdU. Cells were then harvested by trypsinization, fixed with 4% formaldehyde for 15 min at RT, and permeabilized using a buffer containing 0.2% Triton X-100 (replacing the standard saponin). Incorporated EdU was detected via the Click-iT reaction using the Alexa Fluor 488 picolyl azide dye. After the reaction, the cells were stained with 4′,6-diamidino-2-phenylindole (DAPI, 1 μg/ml final concentration) for 10 min to label total DNA content. Last, the cells were analyzed on a Beckman Coulter CytoFLEX LX flow cytometer. Alexa Fluor 488 fluorescence (EdU signal) was detected in the B525 (fluorescein isothiocyanate) channel, and DAPI fluorescence (DNA content) was detected in the UV450 channel. Data acquisition was performed using CytExpert software (version 2.6.0.105). No-EdU controls were included to set background fluorescence thresholds for gating proliferating cells. The experiment was repeated three times with different cell passages.

### Receptor-binding studies

The binding affinity for IR-A of the human IR (IR-A) was determined using the method of Morcavallo *et al.* (*64*) using IM-9 lymphocytes, which are rich in IR-A expression. For the assay, 2.0 × 10^6^ cells per ml were incubated with increasing concentrations of insulin or an analog and human [^125^I]monoiodotyrosyl-A14-insulin [2200 Ci.mmol^−1^, 20,000 cpm, 0.01 nM, prepared as described in (*68*)] for 2.5 hours at 15°C in Hepes binding buffer [100 mM Hepes, 100 mM NaCl, 5 mM KCl, 1.3 mM MgSO_4_, 1 mM EDTA, 10 mM glucose, 15 mM NaOAc, and 1% bovine serum albumin (w/v) (pH 7.6)] (500 μl)]. After incubation, 2 × 200 μl was centrifuged at 13,000*g* for 10 min. Radioactive pellets were counted using a Wizard 1470 Automatic γ Counter (PerkinElmer Life Sciences). Each point was determined in duplicates. Binding data were analyzed with GraphPad Prism 8 using a nonlinear regression and one-site fitting program, which takes the potential ligand depletion into account. The dissociation constant (*K*_d_) was determined from at least three independent measurements. The *K*_d_ of human [^125^I]monoiodotyrosyl-A14-insulin was set to 0.3 nM.

Receptor binding affinity for IR-B was determined using mouse embryonic fibroblasts derived from IGF-1R knock-out mice and transfected with the human IR-B isoform according to Kosinova *et al.* (*69*). For the assay, the cells (about 14,000 per well) were washed twice with the binding buffer [100 mM Hepes (pH 7.6), 100 mM NaCl, 5 mM KCl, 1.3 mM MgSO_4_, 1 mM EDTA, 10 mM glucose, 15 mM sodium acetate, and 1% bovine (w/v) serum albumin]. The cells were incubated and stirred with increasing concentrations of insulin/analog and human [^125^I]monoiodotyrosyl-A14-insulin (2200 Ci.mmol^−1^, 43,000 cpm, 0.043 nM) for 16 hours at 5°C in the binding buffer (total volume, 250 μl). After incubation, the cells were washed twice with the cold binding buffer and solubilized with 0.1 M NaOH. The solutions of solubilized cells were counted for cell-associated radioactivity. Each point was determined in duplicates. The *K*_d_ of human [^125^I]monoiodotyrosyl-A14-insulin was set to 0.3 nM. Analysis of the binding data was performed as described for IR-A.

Receptor binding affinity for IGF-1R was determined by the same methodology as for receptor binding affinity for IR-B according to Kosinova *et al.* (*69*) but using mouse embryonic fibroblasts derived from IGF-1R knock-out mice and transfected with the human IGF-1R. The cells were grown to about 14,000 per well. As a radiotracer, human [^125^I]-IGF-1 was used [2497 Ci.mmol^−1^, 44,000 cpm, 0.039 nM, prepared as described in (*70*)]. The *K*_d_ of human [125I]-IGF-1 was set up to 0.2 nM. Analysis of the binding data was performed as described for IR-A.

Binding to IGF-2R D11 was measured in plate-based assays according to (*23*), and to IGFBP-3 according to (*24*). For each analog, binding curves were determined in duplicate, and the final *K*_d_ was calculated from at least three independent binding curves. In cases where no binding was observed, only a single binding curve was recorded.

### Receptor activation and signaling assays

Concentration-dependent profiles of receptor phosphorylation in IR-A, IR-B and IGF-1R cells were determined using an In-Cell Western Assay adapted for chemiluminiscence as described previously (*71*, *72*). Nonlinear regression curve fitting was carried out with GraphPad Prism 8 software.

Autophosphorylation of the receptor, Akt and Erk ½ phosphorylation were measured using standard Western blot procedure after 4 hours starving in media without serum and stimulation with 10 nM ligands for 20 min (*65*). All the transfected cells lines (IR-A, IR-B, and IGF-1R) and the permanent SH-SY5Y and U87MG cell lines were analyzed. SH-SY5Y cells were treated with insulin, IGF-1, or **1**_Ins_ for 5 to 240 min to determine the time course of receptor, Akt, and Erk1/2 activation. The membranes were probed with phospho-IGF-1Rβ (Tyr^1135^/1136)/IRβ (Tyr^1150/1151^) (19H7) (catalog no. 2024), phospho-Akt (Thr^308^) (C31E5E) (catalog no. 2965), and phospho-p44/42 MAPK (Erk1/2) (Thr^202^/Tyr^204^) (catalog no. 9101) antibodies. GAPDH (glyceraldehyde-3-phosphate dehydrogenase, D4C6R) (catalog no. 97166) antibody was used for normalization and as a loading control. Total proteins were detected with IGF-1R β (111A9) (catalog no. 3018), insulin receptor β (4B8) (catalog no. 3025), Akt (pan) (C67E7) (catalog no. 4691), and p44/42 MAPK (Erk1/2) (3A7) (catalog no. 9107). All antibodies were purchased from Cell Signaling Technology. Anti-actin (20-33) (catalog no. A5060) was from Sigma-Aldrich.

For detection of autophagy and apoptosis, SH-SY5Y cells were starved for 4 hours, pretreated for 2 hours with analogs, and 0.5 mM MG was added for another 2 hours. The antibodies used were as follows: LC3A/B (D3U4C) XP rabbit mAb, SQSTM1/p62 (D1Q5S) rabbit mAb from Cell Signaling Technology autophagy sampler kit, catalog no. 48768, and apoptosis marker Caspase-9 (C9) mouse mAb catalog no. 9508.

In the case of primary rat neonatal neuronal culture, the medium was supplemented with 2% B27 supplement minus insulin at day 5, and after 4 hours, the cells were stimulated with 10 nM native ligands and analogs for 20 min. Samples were prepared in duplicates. The cells were lysed in electrophoresis SDS sample buffer [100 μl per well of 62.5 mM tris/HCl (pH 6.8), 2% SDS (w/v), 10% glycerol (v/v), 0.01% bromophenol blue (w/v), 0.1 M DTT (w/v), 50 mM NaF, 1 mM Na_3_VO_4_, and 0.5% protease inhibitor cocktail (Sigma-Aldrich)]. The samples were analyzed using standard Western blotting as described for the permanent cell lines.

The data were normalized to GAPDH or actin and related to the value measured in IGF-1 treated sample in the same experiment. Mean ± SD (*n* ≥ 3) values were calculated. The significance of the changes in stimulation of phosphorylation in relation to IGF-1 or to control untreated cells was calculated by GraphPad Prism 8 software, using one-way ANOVA with Dunnett’s test comparing all analogs versus control, i.e., IGF-1 or untreated cells.

### Cryo-EM data collection and processing

**1**_Ins_-saturated complexes of GCN4 leucine zipper fusion constructs IRzip (*73*) and IGF1Rzip (*27*) were generated immediately before vitrification (4:1, **1**_Ins_ to receptor monomer). UltrAuFoil (R1.2/1.3) grids were glow discharged using a GloQube, operated at 30 mA for 3 min. Four microliters of the sample was applied to the grids. Cryo-EM imaging of the IGF1R sample was performed on a Titan Krios G4, equipped with a TFS Falcon 4i camera operated in NanoProbe mode. Cryo-EM imaging of the IR sample was performed on a Titan Krios G4, equipped with a Gatan K3 Biocontinuum camera and a Quantum-GIF energy filter. EPU 3 software was used to automate data collection. For the IGF1Rzip dataset, 166-k particles were reference motion corrected, locally refined and three-dimensional flexible refined to give the final map (3.4 Å). For the IRzip dataset, 352-k particles were NU-refined in C2, reference motion corrected and local refined in C2 to give the final reconstruction at 2.86 Å. Detailed methodology is described in the Supplementary Materials.

### Model building

Models were initiated from pdb: 8TAN (IGF-1R) and pdb: 6PXV (insulin receptor). Native ligands were replaced with **1**_Ins_ generated by mutating MFRV-VILP from pdb: 8TAN using COOT v0.9.8.93. ChimeraX v1.7 was used to rigid body fit the models and remove disordered loops with poor associated map density. The models were then morphed to fit the density with adaptive restraints to the initial models using ISOLDE v1.7. Restraints were released and the coordinates refined within ISOLDE v1.7 and 1.8. Final real space refinement was performed using PHENIX v1.21.1 with reference and Ramachandran restraints activated. Cryo-EM data collection and refinement statistics are provided in table S2.

### Insulin tolerance test

Animal procedures followed the European Communities Council Directive 86/609/EEC and were approved by the Institutional Ethical Committee on Animal Experimentation, Czech Academy of Sciences, Prague (protocol no.121-2023). The experiment was done as described in Páníková *et al.* (*20*). Briefly, male 12-week-old male C57BL/6J mice (weighing 21 to 25 g) purchased from Charles River were randomly divided into three groups of 10 mice each. Before the test, the mice were fasted for 6 hours. Groups of mice were injected with saline, human insulin (0.75 U/kg) or with the analog **1**_Ins_ (0.75 U/kg). One unit (U) is defined as 6 nmol of insulin or analog. Blood glucose was measured with a glucometer (Arkray, Kyoto, Japan) in a drop of blood obtained from the tail vein at times 0, 10, 20, 30, 45, 60, 120, and 150 min after injection of compounds or saline. The data were analyzed in GraphPad Prism 8.0 (San Diego, USA). The significance of the changes induced by treatment was calculated using two-tailed *t* test for independent samples.

### Hyperinsulinemic-euglycemic clamps

Whole-body insulin sensitivity was determined via performing hyperinsulinemic-euglycemic clamp studies in 3- to 4-month-old Sprague-Dawley male rats (Charles River Inc.) similar as previously described (*59*, *60*, *74*, *75*). Initially, catheters were surgically implanted into the carotid artery and jugular vein, respectively, and the animals were allowed to recover for approximately 1 week. Recovery was confirmed by a postoperative body weight within 3% of preoperative weights before studies. All clamps were 240 min in duration and consisted of a 120-min basal period and a 120-min hyperinsulinemic clamp period. At *t* = 0 min, which is the beginning of the basal period, a primed-continuous infusion of [3-^3^H]-glucose (20 μCi bolus, 0.2 μCi/min maintenance; NEN Life Science Products, Boston, MA) was given into the jugular vein and maintained throughout the remainder of the study. The hyperinsulinemic-euglycemic clamp was then initiated at *t* = 120 min by peripheral administration of a primed-continuous infusion of either regular porcine insulin (3 mU kg^−1^ min^−1^) (Sigma-Aldrich) or **1**_Ins_ (normal insulin, *n* = 9; **1**_Ins_, *n* = 8) at an equimolar concentration, and somatostatin (1.5 μg kg^−1^ min^−1^) was also provided to suppress endogenous insulin secretion. A 25% glucose solution was given and periodically adjusted to clamp the plasma glucose concentration at ~140 to 145 mg/dl. Serum samples for determination of [3-^3^H]-glucose and [3-^3^H]-glucose water specific activities were obtained at 10-min intervals during the basal and clamp periods. At the completion of the study, the rats were euthanized by exsanguination under isoflurane anesthesia and tissues were then rapidly excised, weighed, and flash frozen in liquid nitrogen, before storage at −80°C. All animal studies were reviewed and approved by the Einstein IACUC (protocol # 00001852).

### Phosphoproteomics sample preparation and analysis

The preparation of SH-SY5Y cell samples for phosphoproteomic analysis, including cell lysis, phosphopeptide enrichment, liquid chromatography–tandem mass spectrometry (LC-MS/MS) acquisition, and data processing followed published protocols by Nagao *et al.* (*10*) and it is described in detail in the Supplementary Materials. Briefly, SH-SY5Y cells were grown on six-well plates, starved in media without FBS for 4 hours, and stimulated with 10 nM hormones for 15 min. The cells were lysed in freshly prepared, preheated (95°C) 4% deoxycholate, 100 mM tris/Cl buffer (pH 8.5). The protein lysates were reduced using 10 mM tris(2-carboxyethyl)phosphine and alkylated with 40 mM chloroacetamide and digested overnight with LysC/trypsin in 1:50 enzyme:protein ratio. Phosphopeptide enrichment was performed using TiO_2_ beads (Titansphere Phos-TiO bulk, 10 μm) following established protocol without automation (*76*). LC separation was performed on Dionex Ultimate 3000 nano HPLC system online connected with MS instrument. The samples were loaded onto the trap column (C18 PepMap100, 5-μm particle size, 300 μm by 5 mm, Thermo Fisher Scientific). Nano reversed phase column (Aurora Ultimate TS, 25 cm–by–75 μm inside diameter, 1.7-μm particle size, Ion Opticks) was used for LC/MS analysis. Peptide mixture was analyzed on Thermo Fisher Scientific Orbitrap Fusion Lumos by data-independent approach. MS1 scans of peptide precursors were analyzed in Orbitrap in range 350 to 300 mass/charge ratio (*m*/*z*) at 60-K resolution. DIA scans were performed in Orbitrap at 30 K resolution. Precursor mass range 400 to 1000 *m*/*z* was covered by 30 windows 20-Da wide. Activation type was set to higher-energy collisional dissociation with 33% collision energy.

### Phosphoproteome analysis and data processing

The acquired dataset was analyzed using Spectronaut version 19.8.250311.62635 (*77*). The analysis workflow used was directDIA+(Deep) with normalization, incorporating posttranslational modifications (PTM) analysis with the multiplicity option enabled. The analysis was performed using a human proteome database (one protein per gene), which was downloaded in November 2024 and included common contaminants. Search parameters were as follows: trypsin enzyme specificity, with carbamidomethyl (C) as a fixed modification, and acetyl (protein N-term), oxidation (M), and phosphorylation (STY) as variable modifications. The thresholds for peptide spectrum match and protein FDR were set to 0.01, while the minimum PTM localization threshold was set to 0.75.

The resulting data were further processed using Perseus 1.6.15 (*78*). Phosphorylation sites quantified in all six replicates in at least one group were considered valid. Missing values were replaced from normal distribution (width 0.3, down shift 1.8). To identify differentially expressed phosphorylation sites two-sample *t* test was performed with s0 = 0.75. To account for multiple-hypothesis testing, a permutation-based FDR of 0.15 was applied to the dataset in the exploratory mode used for hierarchical clustering and GO analysis, and a stricter FDR of 0.05 was applied later to further distinguish the most probable hits. Kinase-substrate relations and regulatory sites were extracted from PhosphoSitePlus (*79*).

Significant phosphorylation sites (with FDR < 0.15 and fold change greater than 4 or less than −4 compared to control) were selected for further analysis. These sites were centered by subtracting the mean and visualized using the pheatmap R package (version 1.0.12) along with hierarchical clustering [R Core Team (2024). R: A language and environment for statistical computing. R Foundation for Statistical Computing, Vienna, Austria. URL: https://R-project.org/]. GO terms from public repositories (*80*) were assigned to differentially regulated phosphoproteins. The top 10 most prominent biological processes ranked by gene count related to identified phosphorylated proteins are visualized as bar plots for each cluster separately.

sPLS-DA was performed using MetaboAnalyst 6.0 (*81*). Before the analysis, data generated from Spectronaut were preprocessed in Perseus software. Phosphosites were retained in the dataset only if they exhibited 100% valid values in at least one experimental condition. The normalized intensities were transformed using a binary logarithm (log_2_) and subsequently uploaded to MetaboAnalyst. Missing values were imputed by MetaboAnalyst’s default procedures, followed by sPLS-DA modeling to identify discriminative features among the groups. The MS proteomics data have been deposited to the ProteomeXchange Consortium via the PRIDE (*82*) partner repository with the dataset identifier PXD067251.

## Supplementary Materials

### The PDF file includes:

Supplementary Text Figs. S1 to S20 Tables S1 to S6 Legend for data S1 Uncropped blots References
