# Supplementary material for: An engineered insulin analog with dual insulin and IGF-1 receptor agonism and distinct signaling
Source: Sci Adv. 2026 May 15;12(20):eaeb7558. doi: 10.1126/sciadv.aeb7558 (PMC13178572; doi:10.1126/sciadv.aeb7558)
Supplement: Supplementary file 1 — Supplementary Text Figs. S1 to S20 Tables S1 to S6 Legend for data S1 Uncropped blots References [file sciadv.aeb7558_sm.pdf]

Science Advances
AAAS

Supplementary Materials for

An engineered insulin analog with dual insulin and IGF-1 receptor agonism and distinct signaling

Irena Selicharová *et al.*

Corresponding author: Jiří Jiráček, jiracek@uochb.cas.cz

Sci. Adv. **12**, eaeb7558 (2026)
DOI: 10.1126/sciadv.aeb7558

The PDF file includes:

Supplementary Text
Figs. S1 to S20
Tables S1 to S6
Legend for data S1
Uncropped blots
References

Other Supplementary Material for this manuscript includes the following:

Data S1

Supplementary methods: **Synthesis of analog $\mathbf{1}_{Ins}$ ([GluB10, D-HisB24 GlyB31, TyrB32]-insulin)**

The analog $\mathbf{1}_{Ins}$ was prepared by total chemical synthesis using orthogonal protection of cysteine thiol groups following the protocol Liu et al. (*83*). The insulin chains were synthesized by solid-phase synthesis (SPSS) on a Spyder Mark IV Multiple Peptide Synthesizer (European Patent application EP17206537.7) developed at the Development Center of the IOCB, using Rink Amide AM resin for chain A and Fmoc-Tyr(tBu)-Wang resin for chain B.

Chain A. Synthesis began with the attachment of the C-terminal amino acid, Fmoc-Asp(OtBu)-OH, to Rink Amide AM resin via the β-carboxyl group of its side chain. Amino acids corresponding to insulin positions A6–A11 and A20 were coupled manually: Fmoc-Cys(tBu)-OH at A6, Fmoc-Cys(Acm)-OH at A7, the isoacyl dipeptide Boc-Ser[Fmoc-Thr(tBu)]-OH at A8 and A9, Fmoc-Ile-OH at A10, and Fmoc-Cys(Mmt)-OH at A11, as well as Fmoc-Cys(Trt)-OH at position A20. Use of the isoacyl dipeptide improved the solubility of the protected peptide chain. Each coupling step employed 3 equivalents of the protected amino acid, HBTU, and HOBt, along with 6 equivalents of DIPEA, in DMF. Reactions were carried out for 2 hours and monitored using the Kaiser test. Fmoc groups were removed using 20% piperidine in DMF (5 and 20 minutes). The remaining amino acids were coupled to the resin using an automated synthesizer, without Kaiser test monitoring. Stock solutions were prepared as follows: 0.4 M Fmoc-protected amino acids dissolved in 0.45 M HOBt in DMF; 20% piperidine in DMF; 0.4 M HBTU in DMF; 1 M DIC in DMF; and 1 M DIPEA in DMF.

After peptide synthesis, the resin was treated with 25% β-mercaptoethanol in DMF for 1.5 hours at room temperature (RT). The treatment was then repeated for an additional 1.5 hours. A small resin sample was analyzed to confirm the removal of the StBu protecting group from the cysteine residue at position A6. The resin was subsequently washed with DMF and DCM, then treated with 10 equivalents of DTNP (2,2′-dithiobis(5-nitropyridine)) in DCM for 1 hour at RT. After the reaction, the resin was again washed with DMF and DCM, followed by treatment with 1% TFA and 5% TIS in DCM (5 × 2 minutes) at RT. Finally, the resin was washed again with DMF and DCM, and stirred in DCM for 1 hour at RT.

The peptide was cleaved from the resin using TFA/TIS/H_2O (95:2.5:2.5, v/v/v) for 1.5 hours, precipitated with cold diethyl ether, and purified by HPLC on a Nucleosil 100-7 C8 column (250 × 10 mm, 7 μm, Macherey-Nagel) using a Waters HPLC system (Waters 600 with 2487 Dual λ Absorbance Detector) at a flow rate of 4 ml/min. Separation was achieved using a gradient of acetonitrile in water with 0.1% TFA (solvent A: 0.1% TFA (v/v) in H_2O; solvent B: 0.1% TFA (v/v) in 80% CH_3CN): t = 0 min, 10% B; t = 30 min, 100% B. Compounds were detected at 218 nm. Peptide purity was assessed on a Nucleosil 120-5 C8 column (250 × 4.6 mm, 5 μm, Macherey-Nagel) at a flow rate of 1 ml/min using the same gradient and solvents on a Watrex HPLC system (Watrex DeltaChrom™ P200 binary pump with Wufeng LC-100 UV detector). Compound identity was confirmed by mass spectrometry.

Chain B. Chain B was synthesized on the same automated peptide synthesizer using the same reagents as for chain A. Cleavage was performed with TFA/TIS/H_2O (95:2.5:2.5, v/v/v) in the presence of 15 equivalents of DTNP for 2 hours. The workup was carried out as described above, and the peptide was subsequently purified and analyzed as previously described. Fmoc-Cys(Acm)-OH was used at position B7 and Fmoc-Cys(Trt)-OH at position B19.

Ligation of A and B chains. The A and B chains were dissolved in 6 M urea and 0.2 M NH_4HCO_3 buffer (pH 8), and the solution was stirred until fully dissolved. After an additional 5 minutes at room temperature (RT), 25 equivalents (based on chain A) of freshly prepared iodine in AcOH were added. The resulting solution was stirred gently for 10 minutes at RT, after which a 1 M solution of ascorbic acid was added dropwise until the color of iodine disappeared. The solution was then diluted with water and purified by RP-HPLC as described above.

The purity (>95%) of the final $\mathbf{1}_{\mathrm{Ins}}$ preparation was assessed as described above by RP-HPLC and monitored at 218 nm (Fig. S1A). The identity of the product was confirmed by high-resolution mass spectrometry (HRMS) (Fig. S1B).

Supplementary Methods: **CryoEM data collection and processing**

UltrAuFoil (R1.2/1.3 300) grids were glow discharged using a GloQube (Quorum), operated at 30 mA for 3 minutes. 4 uL of sample was applied to the grids, which were then blotted using a VitroBot Mk IV (TFS) at 4 °C/100% humidity with blot force 1 for 5 s and plunge frozen in liquid ethane.
CryoEM imaging of the IGF1R sample was performed on a Titan Krios G4, equipped with a TFS Falcon 4i camera operated in NanoProbe mode. EPU 3 software was used to automate data collection. A nominal magnification of 75,000× was used, giving a calibrated specimen level pixel size of 1.036 Å. A C2 condenser aperture of 50 μm and objective aperture of 70 μm were used with a dose rate of 8 e^{-} $pixel^{-1}$ s^{-1}. Movies were collected using a 6.0 s exposure time fractionated into 50 sub-frames resulting in a total accumulated dose of 50 e^{-} $Å^{-2}$ per movie. 10027 movies were collected in faster acquisition mode (AFIS) at a defocus range of -0.5 μm to -2.0 μm from one grid over two sessions.

CryoEM imaging of the IR sample was collected with a Gatan K3 Biocontinuum camera equipped with a Quantum-GIF energy filter. EPU 3 software was used to automate data collection. Imaging was performed in NanoProbe energy filtered zero loss mode using a 10 eV slit width. A nominal magnification of 105,000× was used, giving a calibrated specimen level pixel size of 0.833 Å. A C2 condenser aperture of 50 μm and objective aperture of 70 μm were used and the K3 camera was operated in correlated double sampling mode at a dose rate of 12.5 e^{-} $pixel^{-1}$ s^{-1}. Movies were collected using a 3.00 s exposure time fractionated into 50 sub-frames resulting in a total accumulated dose of 53.8 e^{-} $Å^{-2}$ per movie. 5384 movies were collected in faster acquisition mode (AFIS) at a defocus range of -0.5 μm to -2.0 μm from one grid.

All processing was performed within cryoSPARC v4.5. For the IGF1Rzip dataset, movies were patch motion corrected and patch CTF estimation was performed. Movies with poor motion statistics, CTF fit > 4 Å or contaminating or thick ice were removed, leaving 6473 micrographs. Template picking was performed, using templates generated from a 3D map generated from a screening dataset. Particles were extracted, binned 4x (box size 64 px) and 2D classified (6.9 M particles, 3.6 M retained). Ab intio reconstruction and iterative heterogeneous refinement were performed against 4 classes. One class was retained with 809k particles that were then re-extracted at full resolution (box size 288 px). Further iterative heterogeneous refinement against 3 classes refined the particles to 509k particles. 3D classification without alignment was performed against 10 classes, and four classes with poor models were removed, leaving 304k particles. Local refinement was performed, before CTF refinement, correcting for tilt, trefoil and anisotropic magnification and a second local refinement. Heterogeneous refinement against two identical models was performed to find the very best particles, leaving 166k particles that were reference motion corrected, locally refined and 3D flexible refined to give the final map (3.4 Å).

For the IRzip dataset, movies were patch motion corrected and patch CTF estimation was performed. Movies with poor motion statistics, CTF fit > 4 Å or contaminating or thick ice were removed, leaving 4694 micrographs. Template picking was performed, using templates generated from a 3D map generated from a screening dataset. Particles were extracted, binned 6x (box size 60 px) and 2D classified (4 M particles). *Ab initio* reconstruction and iterative heterogeneous refinement were performed against 3 classes. One class was retained with 1.6 M particles that were then re-extracted binned 2x (box size 180 px). Particles were then iteratively heterogeneous refined, extracted at full resolution (box size 360 px) and non-uniform refined with imposed C2 symmetry (1.1 M particles). Re-balance orientations was performed, leaving 671k particles. 3D classification without alignment was

performed against 8 classes, and two classes with poor models were removed, leaving 548k particles. Local refinement with C2 symmetry was performed, before CTF refinement, correcting for tilt, trefoil and anisotropic magnification and a second local refinement. Heterogeneous refinement against two identical models was performed to find the very best particles, leaving 352k particles that were NU-refined in C2, reference motion corrected and local refined in C2 to give the final reconstruction at 2.86 Å.

CryoEM data collection and refinement statistics are provided in Supplementary Table S2.

Supplementary methods: **Contribution of individual receptors to the dual receptor activity of $\mathbf{1_{Ins}}$**

To support the theory that hybrid receptors contribute to hormone signaling, we used two breast cancer cell lines to examine the dual-receptor activity of $\mathbf{1}_{Ins}$ in additional cellular models. These breast cancer cell lines were originally used to formulate the Theory of Random Assembly (*29*) describing the formation of hybrid receptors. We also carried out experiments using a specific IR inhibitor to distinguish the contributions of individual receptor species.

Cell lines

Breast cancer cell lines MCF-7 (ATCC, HTB-22™) and MDA-MB-231 (ATCC, HTB-26™) were cultured in DMEM supplemented with 10% fetal bovine serum, 1% penicillin/streptomycin, and 2 mM L-glutamine at 37 °C in a humidified incubator with 5% CO_2.

The expression levels of IR-A, IR-B, and IGF-1R mRNA were determined using standard RT-qPCR procedures as described for the other cell models. The primers used are listed in Table S4.

Stimulation of cells for Western blot analysis

Cells were cultured in 24-well plates and stimulated with 10 nM ligands for 20 min, as described in the main text. The SH-SY5Y cell line was included, with the cell number doubled compared to our original protocol. Cells were also co-stimulated with insulin and IGF-1 (5 + 5 nM or 10 + 10 nM). Prior to stimulation, cells were preincubated with 100 nM S661 peptide (*31*) for 5 min, and subsequently treated with the ligands. The samples were run in parallel on two gels; total protein levels were detected on the second gel. Membranes were probed with antibodies against phospho-IGF-1Rβ (Tyr1135/1136)/IRβ (Tyr1150/1151) (19H7), phospho-Akt (Thr308) (C31E5E), and phospho-p44/42 MAPK (Erk1/2) (Thr202/Tyr204). Total proteins were detected using antibodies against IGF-1 Receptor β (111A9), Akt (pan) (C67E7), and p44/42 MAPK (Erk1/2) (3A7). Anti-actin (20-33) or GAPDH (D4C6R) antibodies were used for normalization and as loading controls.

The data were normalized to actin and expressed relative to the signal obtained in IGF-1–treated samples within the same experiment. Values represent mean ± SD ($n \geq 3$).

Supplementary results and discussion: **Contribution of individual receptor to the dual receptor activity of $\mathbf{1_{Ins}}$**

Receptor expression

We determined the mRNA expression levels of the receptors in the breast cancer cell lines MCF-7 and MDA-MB-231 to enable direct comparison with our data obtained from the SH-SY5Y cell line. The theoretical receptor isoform distribution was evaluated as described for the other cell models (Table S5; see also Table S3). In addition, we compared the amounts of receptors (IR and IGF-1R) as well as total Akt, Erk1/2, actin, and GAPDH by Western blotting (Fig. S9). The Western blot data were consistent with the mRNA expression profiles. It should be noted that the relative abundance of IR and IGF-1R in our cell lines differed from that reported by Pandini *et al. (29)*, who measured 22 % hybrid receptors in MCF-7 and 54 % in MDA-MB-231 cells. Based on mRNA analysis, we observed nearly exclusive expression of IGF-1R in MCF-7 cells, with approximately 3 % hybrids. The theoretical proportion of

hybrids was 14 % in MDA-MB-231 and 26 % in SH-SY5Y cells. According to the theory of random assembly, a detectable amount of IR homodimers could be expected only in SH-SY5Y cells (≈2 %), while in the other cell lines it would be below 1 %. We speculate that the observed differences in receptor expression may result from the use of nutrient-rich growth medium (DMEM with high glucose), which could favor a proliferative phenotype driven by IGF-1R signaling.

Treatment with S661 peptide, an IR inhibitor

The S661 peptide has a sequence entirely unrelated to insulin (*31*). Nevertheless, it binds with high affinity to IR-A and inhibits receptor activation. We have recently determined the structure of the S661–IR-A complex (*32*). The inhibitor engages both the Site 1 and Site 2 regions of the insulin receptor. Consistent with this, it does not bind to IGF-1R (Fig. S10A) nor does it affect IGF-1R signaling (Fig. S10B).

Next, we treated three different cell lines with insulin, IGF-1, their combination (5 or 10 nM each), and with $\mathbf{1}_{Ins}$ in the absence or presence of the S661 inhibitor (100 nM). The experiment was performed in three independent replicates. Band densities were normalized to actin and expressed relative to the IGF-1 signal (Fig. S11).

The inhibition (Fig. S11) did not affect signaling in the MCF-7 cell line, which expresses predominantly IGF-1R and only a minor population of hybrid receptors (2–3%). In contrast, S661 reduced signaling induced by all ligands in the SH-SY5Y and MDA-MB-231 cell lines, which contain substantial fractions of hybrid receptors (26% and 14%, respectively).

The decrease in the IGF-1 signal in these two cell lines strongly suggests that the S661 inhibitor can target hybrid receptors, even though it is not capable of inhibiting IGF-1R itself.

Based on previous studies of synthetic peptides containing the same Site 1 or Site 2 key binding motifs (e.g. FYDWFERQ and SLEEEWAQ, respectively) as S661, the Site 1 motif of S661 binds to the L1–αCT region of both the insulin receptor and IGF-1R with low-nanomolar affinity (*84*). The Site 2 motif has not been tested against IGF-1R, but it binds to the insulin receptor with high-nanomolar to low-micromolar affinity (*85)*. Activation of the insulin receptor requires engagement of both Site 1 and Site 2. Thus, we can speculate that S661 can inactivate hybrid receptors owing to its ability to cross-link the IGF-1R Site 1 region with the IR Site 2 region.

Conversely, the inability of S661 to activate IGF-1R likely reflects unfavorable interactions between the S661 Site 2 motif and the IGF-1R Site 2 region. Several key residues that mediate Site 2 peptide binding in IR are absent in IGF-1R. IR-Tyr477 and IR-Arg479 form a hydrogen-bonding network with Glu5 of Site 2 peptides, whereas the corresponding IGF-1R residues (Ser467 and Thr469) cannot support equivalent interactions. Likewise, IR-Arg488, which can form a salt bridge with Glu12, is replaced by IGF-1R-Thr478, which cannot reproduce this interaction. Based on the results of Kirk et al. (*86*), disruption of either of these interactions significantly decreases affinity and receptor phosphorylation.

We cannot conclusively determine whether co-stimulation with insulin and IGF-1 produced the same effect as stimulation with $\mathbf{1}_{Ins}$, nor can we rule out a difference between the reduced S661 signal upon $\mathbf{1}_{Ins}$ versus IGF-1 stimulation. The observed changes in signal intensities did not reach statistical significance. However, $\mathbf{1}_{Ins}$ appeared to outperform IGF-1 in all tested cell lines, including MCF-7. This may be attributable to the faster onset of autophosphorylation induced by $\mathbf{1}_{Ins}$, as observed in the time-course experiment in SH-SY5Y cells (Fig. S15).

The results presented in Fig. S11 further suggest that the cellular effects of $\mathbf{1}_{Ins}$ are at least partially mediated through binding to and activation of hybrid receptors. This is supported by the observation that $\mathbf{1}_{Ins}$ retains approximately 80% of the binding affinity of IGF-1—virtually equivalent—and displays no appreciable difference in receptor activation at a concentration of 10 nM (Fig. S12 and Fig. S13C).

To explain the observed behavior of $\mathbf{1}_{Ins}$, we speculate that $\mathbf{1}_{Ins}$ may stimulate hybrid receptors more effectively than IGF-1, which could also account for the distinct phosphorylation profiles detected by phosphoproteomics. The mode of interaction of $\mathbf{1}_{Ins}$ with hybrid receptors remains elusive. We speculate that $\mathbf{1}_{Ins}$ may engage both the Site 1a and Site 1b elements contributed by the insulin receptor and IGF-1 receptor within the hybrid. In contrast, IGF-1 does not interact favorably with the insulin receptor Site 1b and especially Site 2 (*43*), which could make $\mathbf{1}_{Ins}$ more effective in activating hybrid receptors.

Receptor residence time and internalization dynamics should also be taken into account. As shown in the time-course profile, no marked differences were observed among insulin, IGF-1, and $\mathbf{1}_{Ins}$ with respect to Akt and ERK1/2 activation (Fig. S15). However, phosphorylation of IGF-1R initially appeared to be more strongly stimulated by $\mathbf{1}_{Ins}$ than by IGF-1, before stabilizing at a similar level at later time points (Fig. S15A). This transient difference may help explain the divergence in downstream cellular signaling.

It is also important to note that $\mathbf{1}_{Ins}$ is not affected by binding to IGF-binding proteins (IGFBPs). SH-SY5Y cells can express IGFBPs, which may influence long-term stimulation outcomes—such as those detected in MTT assays (Fig. 4 and Fig. S16)—because IGF-1 can be sequestered by IGFBPs secreted by the cells. However, IGFBPs are not expected to affect short-term signaling experiments (20 min), as the medium is replaced with fresh ligand-containing medium immediately before stimulation.

Supplementary Methods: **Methodology for processing of samples for phosphoproteomics - cell lysis and LC-MS/MS**

Preparation and processing of samples for phosphoproteomics followed published protocols (*10*). SH-SY5Y cells were grown on six-well plates to 80-90% confluence. They were starved in media without FBS for 4 hours and stimulated with 10 nM hormones for 15 min. We prepared six separate wells for each treatment, i.e. control non-stimulated cells and cells stimulated with insulin, IGF-1 and with $\mathbf{1}_{Ins}$. The samples were evenly distributed on four six-well plates. The cells were lysed in freshly prepared, preheated (95 °C) 4% deoxycholate, 100 mM Tris/Cl buffer pH 8.5. The samples were ultrasonicated using a probe (Bandelin Sonoplus) for 10 s at 70% intensity and heated at 95 °C for 5 min twice. The protein lysates were reduced using 10 mM tris(2-carboxyethyl)phosphine (TCEP) and alkylated with 40 mM chloroacetamide and digested overnight with LysC/trypsin in 1:50 enzyme:protein ratio.

Phosphopeptide enrichment of 350 ug protein per condition was performed using TiO_2 beads (Titansphere Phos-TiO bulk, 10 μm) following established protocol without automation (*76*). Phosphopeptides were separated and analyzed by LC-MS/MS. LC separation was performed on Dionex Ultimate 3000 nano HPLC system online connected with MS instrument. Samples were loaded onto the trap column (C18 PepMap100, 5 μm particle size, 300 μm x 5 mm, Thermo Scientific) for 2 min at 25 μl/min. Loading buffer was composed of water, 2% acetonitrile and 0.1% trifluoroacetic acid. Peptides were eluted with Mobile phase B gradient from 5% to 30% B for 72 min. Mobile phase buffer A was composed of water and 0.1% formic acid. Mobile phase B was composed of acetonitrile and 0.1% formic acid. Nano reversed phase column (Aurora Ultimate TS, 25 cm x 75 μm ID, 1.7 μm particle size, Ion Opticks) was used for LC/MS analysis.

Peptide mixture was analyzed on Thermo Scientific Orbitrap Fusion Lumos by data independent approach. Eluting peptide cations were converted to gas-phase ions by electrospray ionization in Positive mode. Spray voltage was set to 1600 V and ion transfer tube temperature to 275 °C. MS1 scans of peptide precursors were analyzed in Orbitrap in range 350-1300 m/z at 60K resolution and with following settings: RF Lens 30%, maximum injection time 118 ms, AGC target 100. DIA scans were performed in Orbitrap at 30K resolution. AGC target was set to 1000 and maximum injection time mode to Auto. Precursor mass range 400-1000 m/z was covered by 30 windows 20 Da wide. Activation type was set to HCD with 33% collision energy.

***Supplementary Results and Discussion*: Phosphoproteomics analysis**

We performed a global phosphoproteomics analysis on SH-SY5Y cells to uncover potential differences in signaling networks following stimulation with insulin, IGF-1, and $\mathbf{1}_{\mathrm{Ins}}$.

Each condition was analyzed in hexaplicates, and the samples were evenly distributed across four 6-well plates. The plates were stimulated sequentially. Only one insulin-stimulated sample was excluded due to spoilage. No formal outlier analysis was performed. It is likely that excluding potential outliers would yield more statistically significant results. We applied fold-change higher than ± 2 with FDR of 0.15 on the data set for the initial search. For subsequent analyses we restricted the dataset by fold-change higher than ± 4-fold. Later on, FDR of 0.05 was applied to further validate our conclusions (Table S6). The volcano plots (Fig. S20) clearly demonstrate good consistency among treatments, supporting the robustness of our interpretations.

The regulated phosphosites were subjected to hierarchical clustering analysis. Based on the resulting heatmap (main text, Fig. 6B), we classified the phosphosites into six distinct clusters (1–6). We then performed gene ontology analysis for the proteins corresponding to each cluster using the UniProt database (https://www.uniprot.org/) (main text and Fig. S10).

The predominant receptor populations detected in SH-SY5Y cells (Supplementary Table S2) were IGF-1R homodimers and IGF-1R/IR-A hybrid receptors, for which insulin exhibits the lowest binding affinity. IGF-1 was therefore expected to be the principal activator of these receptors. Notably, $\mathbf{1}_{\mathrm{Ins}}$ acquired high affinity for IGF-1R homodimers and was also anticipated to bind the hybrid receptors with increased affinity. Sparse PLS-DA (Partial Least Squares Discriminant Analysis with sparsity constraints) revealed a clear separation between basal and ligand-induced phosphoproteomes. The phosphoproteomic profile of IGF-1 overlapped with that of $\mathbf{1}_{\mathrm{Ins}}$, and both were distinctly separated from insulin (main text, Fig. 6A), confirming that $\mathbf{1}_{\mathrm{Ins}}$, although based on insulin, had acquired IGF-1-like signaling characteristics. Consistently, peptides corresponding to the activation loops of IR and IGF-1R tyrosine kinases were strongly phosphorylated in cells treated with $\mathbf{1}_{\mathrm{Ins}}$ and IGF-1 (30-fold and 20-fold increases, respectively, compared to control), but only weakly in insulin-treated cells (2-fold) (Table S6 and Fig. S13, marked in blue). However, no other phosphosites belonging to the insulin or IGF-1 receptors were detected. This may reflect the fact that we analyzed a single time point—15 minutes post-stimulation. Phosphorylation dynamics likely differ between ligands. We observed a significant number of regulated phosphosites when comparing ligand-treated cells to untreated controls. Moreover, many phosphosites were significantly changed when comparing $\mathbf{1}_{\mathrm{Ins}}$ and IGF-1 to insulin. In contrast, direct comparison between $\mathbf{1}_{\mathrm{Ins}}$ and IGF-1 revealed no statistically significant differences, with the exception of phosphorylation on RL19 (Table S6), although some phosphosites exhibited large fold-changes.

When comparing the phosphoproteomes induced by individual ligands, we observed several noteworthy features. For clarity, we organized the discussion of regulated phosphosites into three hierarchical levels. First, we address changes in the phosphorylation of scaffolding adaptor proteins, which function as key mediators of signal transduction. Second, we focus on phosphosites found on downstream kinases. Finally, we examine phosphosites located on proteins that can be considered terminal or functional effectors of the signaling pathways. All features discussed in the following sections are summarized in Table S6 and visualized in Fig. S20.

Concerning the scaffolding adaptor proteins, we identified altered phosphorylation of Shc1 and insulin receptor substrate 2 (IRS2) (*87*), and also a less-known GRB2-associated-binding protein 2 (GAB2) in the SH-SY5Y cell line (*11*). It was reported that SH-SY5Y cells do not express IRS1 (Ref. (*88*)), and accordingly, we have not detected any IRS1-derived phosphopeptides. We found one phosphosite on Shc1 that was moderately activated by all the ligands (2–4-fold, marked green in Fig.

S13). However, we identified a large number of phosphorylated peptides derived from IRS2 (marked pink in Fig. S13), which seems to be the main mediator of signal transmission in SH-SY5Y cells. Several of the phosphosites were significantly upregulated by ligand treatment, and most importantly, we identified phosphosites differentially regulated by treatment with individual ligands. Complicated phosphorylation patterns were reported for the IRSs (*87, 89*). Tyr phosphorylation provided by the receptor tyrosine kinase enables binding of SH2 domain proteins, while multiple insulin-regulated Ser/Thr kinases phosphorylating IRSs mediate positive or negative feedback regulation of insulin/IGF signaling. In our case, Y919 and S915 were most heavily stimulated by both $\mathbf{1}_{\mathrm{Ins}}$ and IGF-1 (30-fold and 25-fold, respectively, compared to control) and about 2-fold less by insulin (16-fold), while S306 and S309 were stimulated by $\mathbf{1}_{\mathrm{Ins}}$ (30-fold) and about 3-fold less by IGF-1 and insulin (6–11-fold) (pink arrows in Fig. S13). Another 8 activated phosphosites (T520, T527, S523, S577, Y598, Y653, Y675, and Y823) were regulated by all the ligands similarly (between 2- to 15-fold). Here, we cannot submerse into detailed exploration of such IRS phosphorylation patterns. This would deserve future research. Interestingly, the Tyr residues (except Y919) that might be primarily targeted by the receptor tyrosine kinase were phosphorylated almost equally by all the ligands regardless of their affinity (Table S6). The fact that S306 and S309 sites were differentially phosphorylated by $\mathbf{1}_{\mathrm{Ins}}$ treatment might indicate faster or differential launching of feedback mechanisms rather than differential activation by the receptor tyrosine kinase. It would be interesting to check the IRS2 phosphorylation patterns at various time points. It is anticipated that the phosphorylation patterns that we observe on adaptor proteins after 15 min treatment result mostly from feedback regulation (*88*) as the signal was already delivered to the cells and activation is expected to be suppressed.

Another docking protein, GAB2 (marked brown in Fig. S13), that was reported to mediate tyrosine kinase signaling to Akt and Erk (*11, 90*), was almost exclusively stimulated by $\mathbf{1}_{\mathrm{Ins}}$ on Y614 and S623 (18-fold), compared to 2–3-fold stimulation by IGF-1 and insulin treatment (brown arrows in Fig. S13), while it was phosphorylated on Y643 and S264 equally by all the ligands (3–6-fold). In contrast to IRS2, this adaptor protein might be differentially phosphorylated by the receptor kinase on Y614 as a consequence of ligand binding. However, what lies behind this exclusive action of $\mathbf{1}_{\mathrm{Ins}}$ can only be speculated. It might be a differential mode of activation of hybrid receptors, as $\mathbf{1}_{\mathrm{Ins}}$ has unique binding characteristics that perhaps could lead to the induction of a different conformation of the hybrid receptor, and consequently, differential signaling.

Other docking proteins were found to be substantially phosphorylated upon the treatments. Embryonal Fyn-associated substrate (EFS) (*91*) was equally phosphorylated by all the ligands including insulin (marked green in Fig. S13), while phosphorylation of E3 ubiquitin-protein ligase SH3RF1 (SH3R1) (*92*) was significantly induced in the $\mathbf{1}_{\mathrm{Ins}}$-treated cells (marked red in Fig. S13). EFS is a member of the Crk-associated protein family, reported to associate with focal adhesion kinase (FAK) and Src family kinases to activate downstream effectors regulating the actin cytoskeleton (*91*). SH3R1 was reported (besides its own E3 ubiquitin-protein ligase activity) to organize components of the c-Jun N-terminal kinase (JNK) pathway, linking activated Rac-1 and downstream modules of the pathway (*91*). The phosphosites S735 and S739 of SH3R1 were almost 50-fold upregulated in the $\mathbf{1}_{\mathrm{Ins}}$-stimulated cells, compared to about 5-fold stimulation in the IGF-1- and insulin-treated cells. However, it is not clear what regulatory events are related to this phosphorylation pattern.

Concerning downstream kinases, the central roles of kinases Akt and Erk1/2 in the two major signaling pathways by which IR/IGF-1R regulate metabolism and gene expression are well established (*11*). We identified Akt3 in the SH-SY5Y cells, which seems to be an isoform typical for the brain (*93*). The phosphorylation of Akt3 appeared as the most prominent change in the SH-SY5Y cell phosphoproteome (marked blue in Fig. S13B). It was phosphorylated on both Thr305, which is a target of PI3K, and Ser472 and Ser476, which are phosphorylated by mTOR (*94*). It was equally phosphorylated in the treatments with $\mathbf{1}_{\mathrm{Ins}}$ and IGF-1, and less so by insulin, which correlated with the

pattern found in receptor tyrosine kinase autophosphorylation. Both Erk1 and Erk2 (Ref. (*11*)) (marked green in Fig. S13B) were similarly phosphorylated in treatments with all the ligands, with a slight decrease in the order $\mathbf{1}_{\mathbf{Ins}}$ > IGF-1 > insulin.

We detected significantly regulated phosphosites on many other kinases and regulatory proteins known to be involved in insulin/IGF signaling. Some of them were equally stimulated by all three ligands (marked green in Fig. S13B). Among them is TSC2, which forms a complex that inhibits mTOR and is a target of Akt. Its phosphorylation at Thr1462 relieves the mTOR inhibition and promotes proliferation (*95*). TSC2 was equally phosphorylated (at least at the 15 min time point) by all the ligands, regardless of differential activation of Akt. Similarly, we detected equal phosphorylation of FOXO3 (a transcription factor known to be regulated by insulin/IGF signaling (*96*, *97*)) at Thr32 and Ser43 by all the ligands. On the other hand, downstream kinases such as ribosomal protein S6 kinase alpha-3 (KS6A3), a member of the 90 kDa ribosomal S6 kinase (RSK) family, or serine/threonine-protein kinase D3 (PKCD3) appeared to be differentially phosphorylated by the ligands (marked light violet in Fig. S13B).

RSKs are downstream effectors of the MAPK pathway (*98*). We observed similar trends in the phosphorylation of two of them (KS6A3 and KS6A1) as for Erk, i.e., $\mathbf{1}_{\mathbf{Ins}}$ > IGF-1 > insulin. However, this trend was intensified in the case of KS6A3 ($\mathbf{1}_{\mathbf{Ins}}$ ~ 70-fold > IGF-1 ~ 30-fold > insulin ~ 5-fold, Table S6 and Fig. S20B). RSKs were originally discovered as kinases responsible for ribosomal protein S6 phosphorylation, which was correlated with the initiation of protein synthesis (*99*). We found RS6 phosphorylation to be substantially increased in the $\mathbf{1}_{\mathbf{Ins}}$-treated cells (Table S6). However, RS6 phosphorylation can be induced by various other mechanisms (*100*), including synergistic crosstalk between the mTORC1 and Erk signaling pathways, as well as regulatory input from the cAMP signaling network. Death-associated protein kinase 1 (DAPK1), whose phosphorylation was also increased in $\mathbf{1}_{\mathbf{Ins}}$-treated cells, was also reported to phosphorylate RS6. Although the role of RS6 phosphorylation(*100*) in protein synthesis is not fully understood, we could speculate that a suggested path (GAB2 → Erk → KS6A3 → RS6 → protein synthesis) might underlie the increased proliferative activity of $\mathbf{1}_{\mathbf{Ins}}$. Besides RS6, we also found phosphorylation of another ribosomal protein, RL19 (Table S6), to be significantly upregulated in the $\mathbf{1}_{\mathbf{Ins}}$-treated cells, which might be connected to this pathway. We identified additional proteins that appeared to be preferentially phosphorylated upon $\mathbf{1}_{\mathbf{Ins}}$ treatment and could be considered terminal targets, such as inositol hexakisphosphate and diphosphoinositol-pentakisphosphate kinase 2 (VIP2) (*101*), which regulates levels of inositol phosphates—important signaling molecules (*101*); death-associated protein kinase 1 (DAPK1); Sin3 histone deacetylase corepressor complex component SDS3 (SDS3) (*102*), a part of the transcriptional repressor complex mediating key epigenetic regulations (*102*); or mediator of DNA damage checkpoint protein 1 (MDC1).

However, we also found proteins preferentially phosphorylated by IGF-1. Among them were PKCD3 (Ref. (*103*), light violet in Fig. S13B), which is an effector of diacylglycerol signaling downstream of protein kinase C (PKC) (*103*), and notably, 3',5'-cyclic-AMP phosphodiesterase 4B (*104*) (PDE4B, marked red in Fig. S13B), which regulates levels of the second messenger cAMP and sits at a crossroads allowing integration of various signaling pathways with that of cAMP (*104*). Both pathways have been reported to interact with IGF-1R signaling (*105*). Nucleolar RNA helicase 2 (DDX21) (*106*) phosphorylation appeared **to be phosphorylated only by IGF-1** (Table S6). Members of the DEAD-box family of RNA helicases are engaged in all facets of RNA metabolism, from biogenesis to decay (*106*). DDX21 was phosphorylated on S168, S171, and S173 solely after IGF-1 treatment. However, we do not yet know how this helicase is regulated by phosphorylation. Coiled-coil domain-containing protein 86 (CCD86) (*107*) is a nuclear protein expressed in the brain and may be involved in immune and apoptosis responses (*107*). This protein was also preferentially phosphorylated by IGF-1.

Another protein involved in mRNA processing, serine/arginine repetitive matrix protein 1 (SRRM1), a part of the spliceosome (*108*), yielded a complicated phosphorylation pattern, where some sites were

upregulated and others downregulated in the treatments, suggestive of massive changes in translation and proliferation in the stimulated SH-SY5Y cells. Interestingly, one phosphosite (Ser775) was substantially more phosphorylated by insulin treatment (Table S6).

We have not thoroughly investigated the phosphosites that were downregulated by the treatments. However, numerous sites appeared to be more markedly downregulated in insulin-treated cells compared to the **1_{Ins}**- and IGF-1-treated cells. For instance, phosphorylation of doublecortin (DCX), a neurogenesis marker essential for neuronal migration (*109*), was significantly reduced in insulin-treated cells, which may affect DCX mobilization. Similarly, phosphorylation of EH domain-binding protein 1, a key player in actin cytoskeleton organization (*110*), was also substantially decreased in the insulin-treated group.

Collectively, these findings show that while **1**$_{Ins}$ recapitulates IGF-1 signaling to a large extent, it also exerts unique regulatory effects on specific adaptor proteins and downstream targets, which may enhance proliferation in neuronal cells.

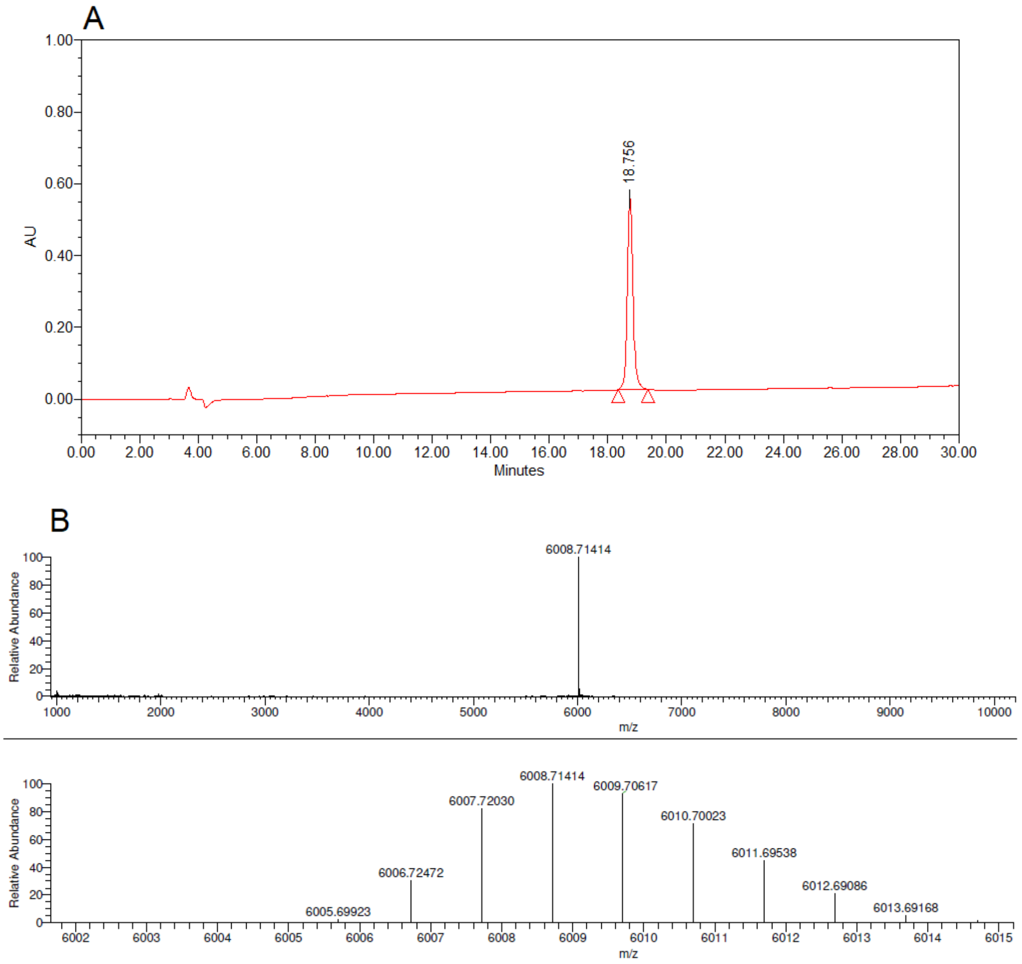

Fig. S1. Analytical data for 1_{Ins}. (A) Analytical HPLC trace of analog **1**$_{Ins}$ and (**B**) high-resolution ESI mass spectrum of analog **1**$_{Ins}$. Calculated: $C_{264}H_{393}N_{67}O_{82}S_6$, Exact Mass: 6005.6966, Molecular Weight: 6009.7950.

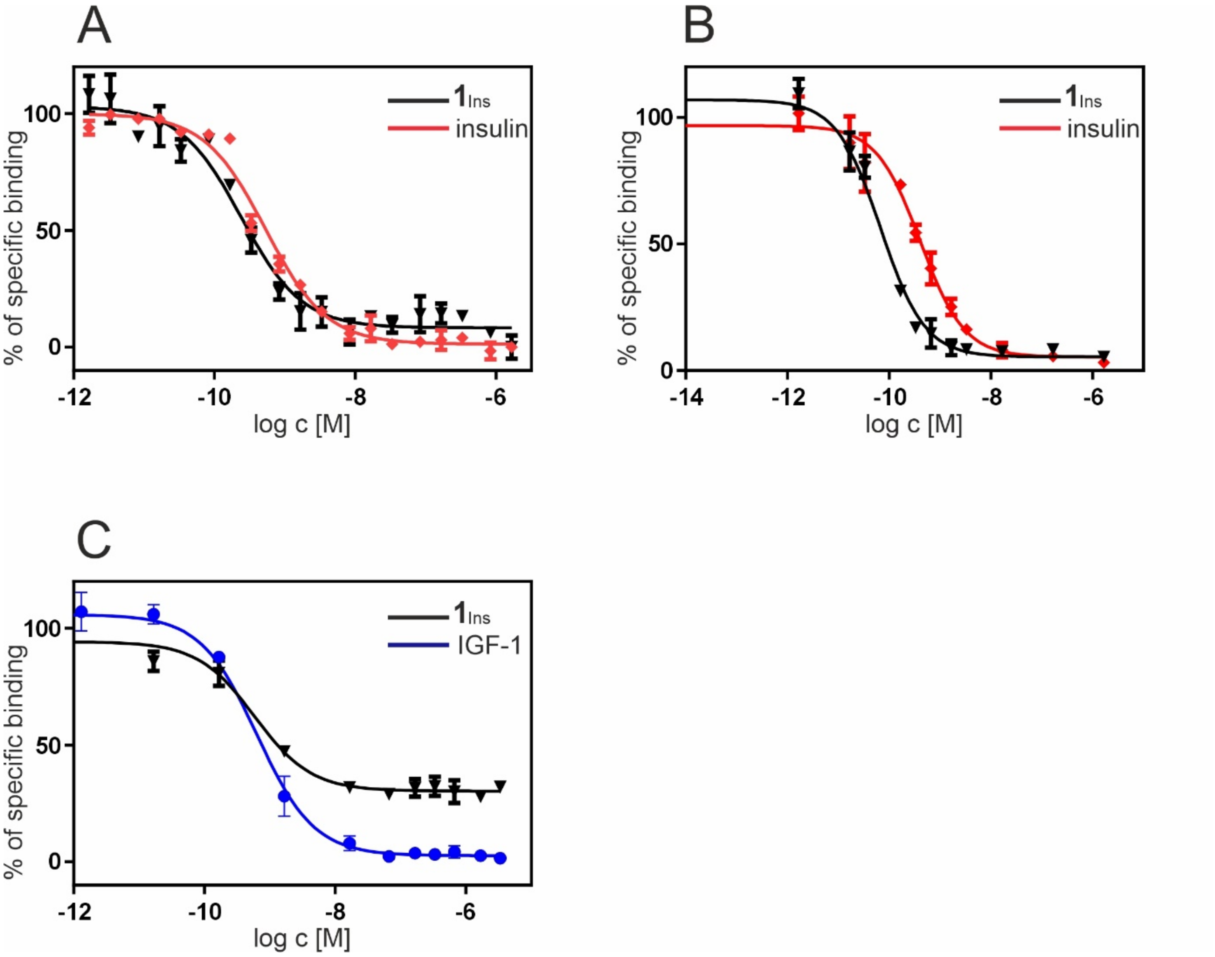

Fig. S2. Representative binding curves of the analog 1_{Ins} ([GluB10, D-HisB24, GlyB31, TyrB32]-insulin). Inhibition of binding of human ^{125}I-Insulin to IR-A in IM-9 cells (**A**). Inhibition of binding of human ^{125}I-Insulin to IR-B (**B**) and human ^{125}I-IGF-1 to IGF-1R (**C**) in mouse fibroblasts transfected with respective human receptors. Representative binding curves show comparison of the inhibition by the analog with human insulin or IGF-1.

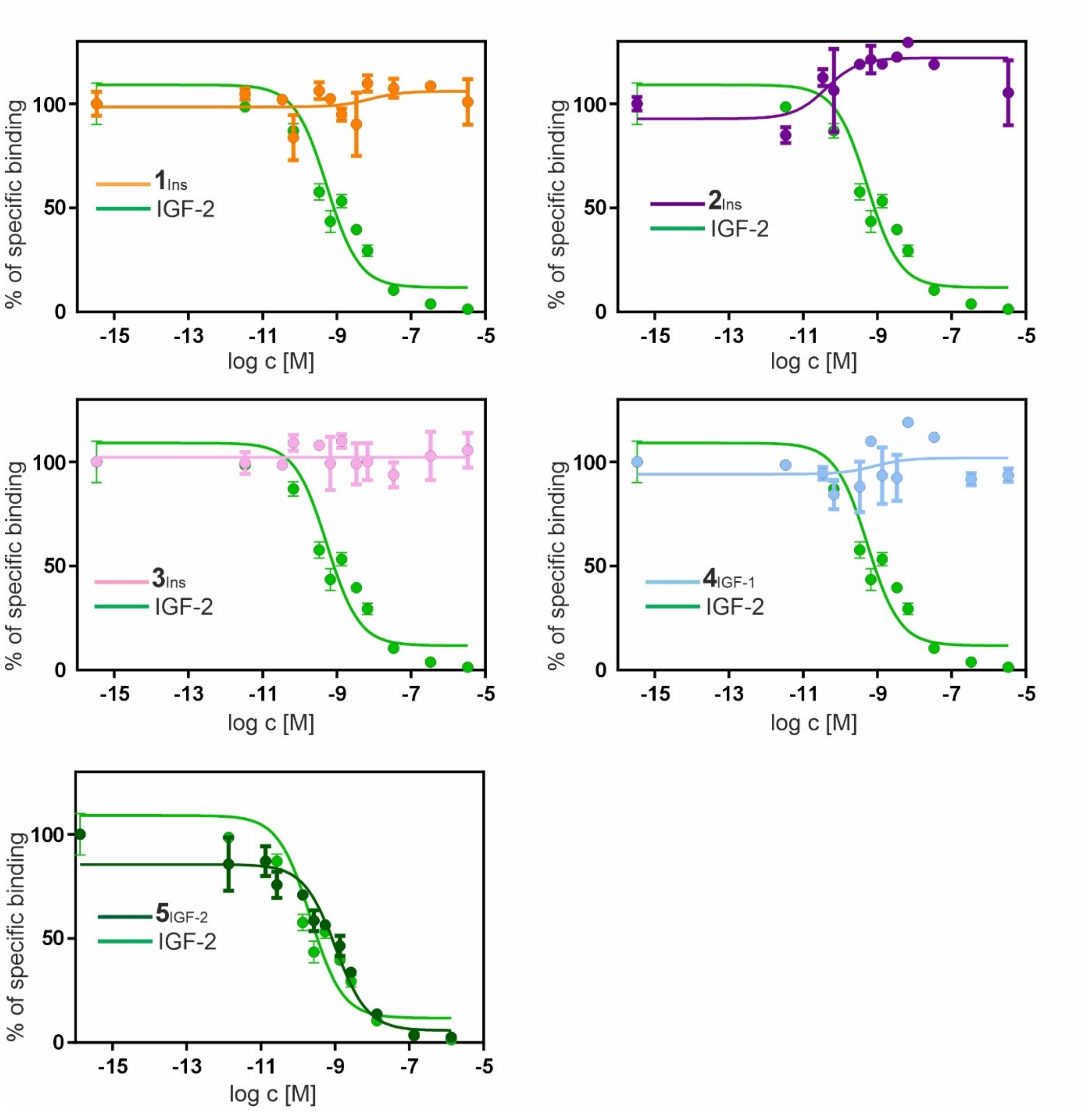

Fig. S3. Binding curves of the analogs on domain 11 of IGF-2R. Inhibition of binding of human ^{125}I-IGF-2 to immobilized IGF-2R domain 11 by the analogs. In each panel, binding curves compare the inhibitory effect of a specific analog with that of human IGF-2.

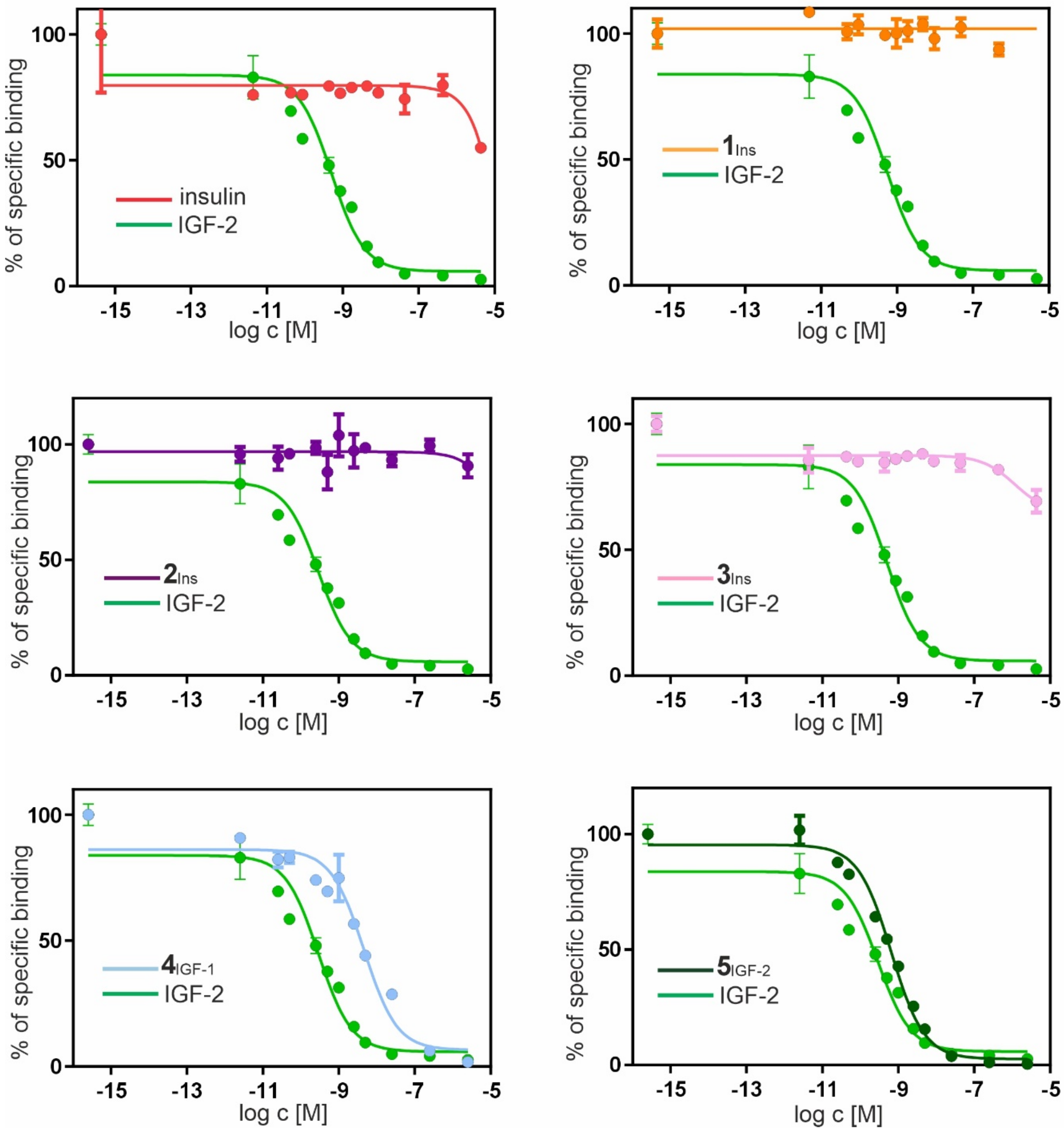

Fig. S4. Binding curves of the analogs on IGFBP-3. Inhibition of binding of human [125]IGF-2 to immobilized IGFBP-3 by the analogs. Binding curves show comparison of the inhibition by the specific analog with human IGF-2.

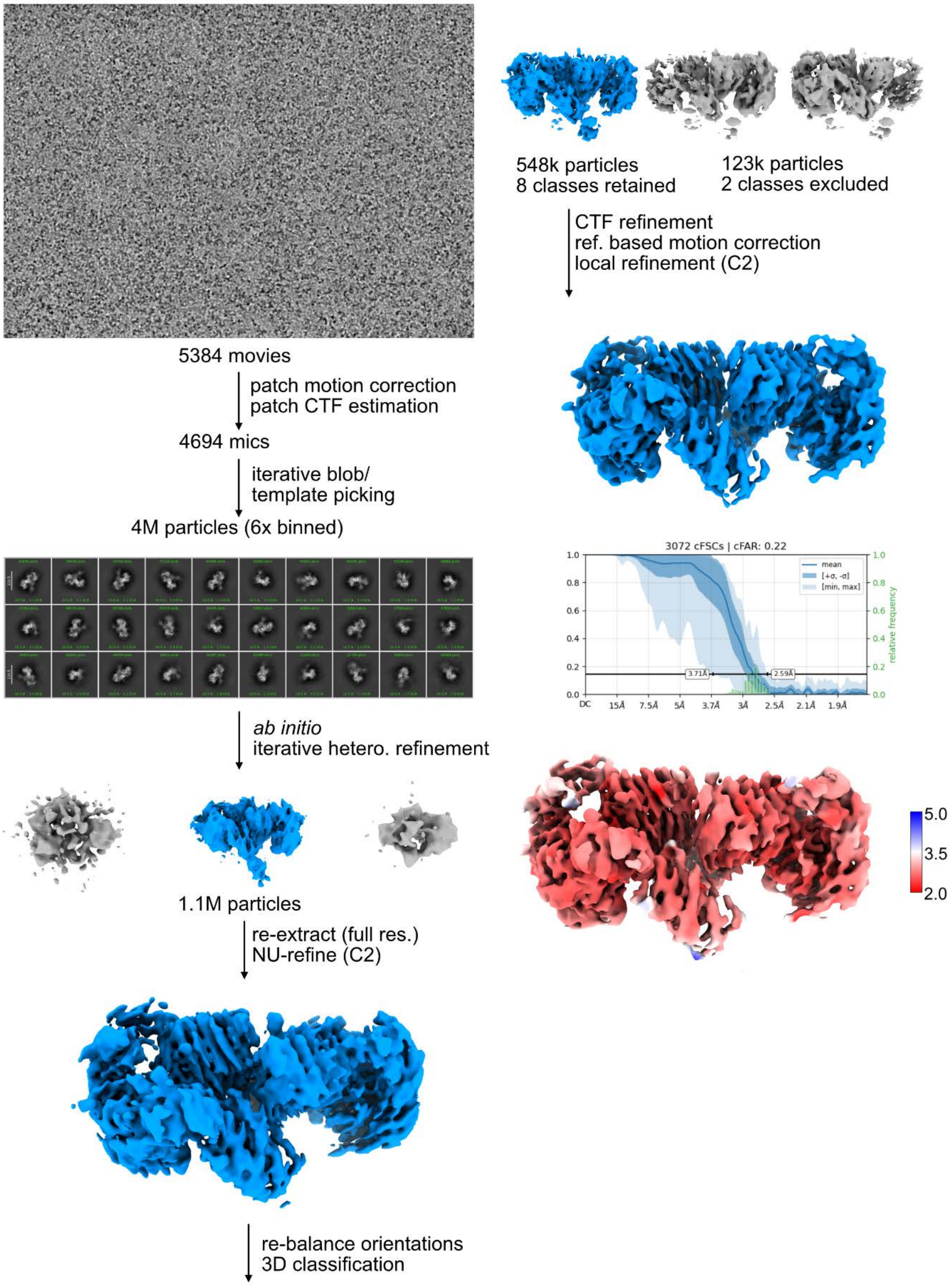

Fig. S5. CryoEM single particle analysis of 1_{Ins}-IR-zip complex dataset.

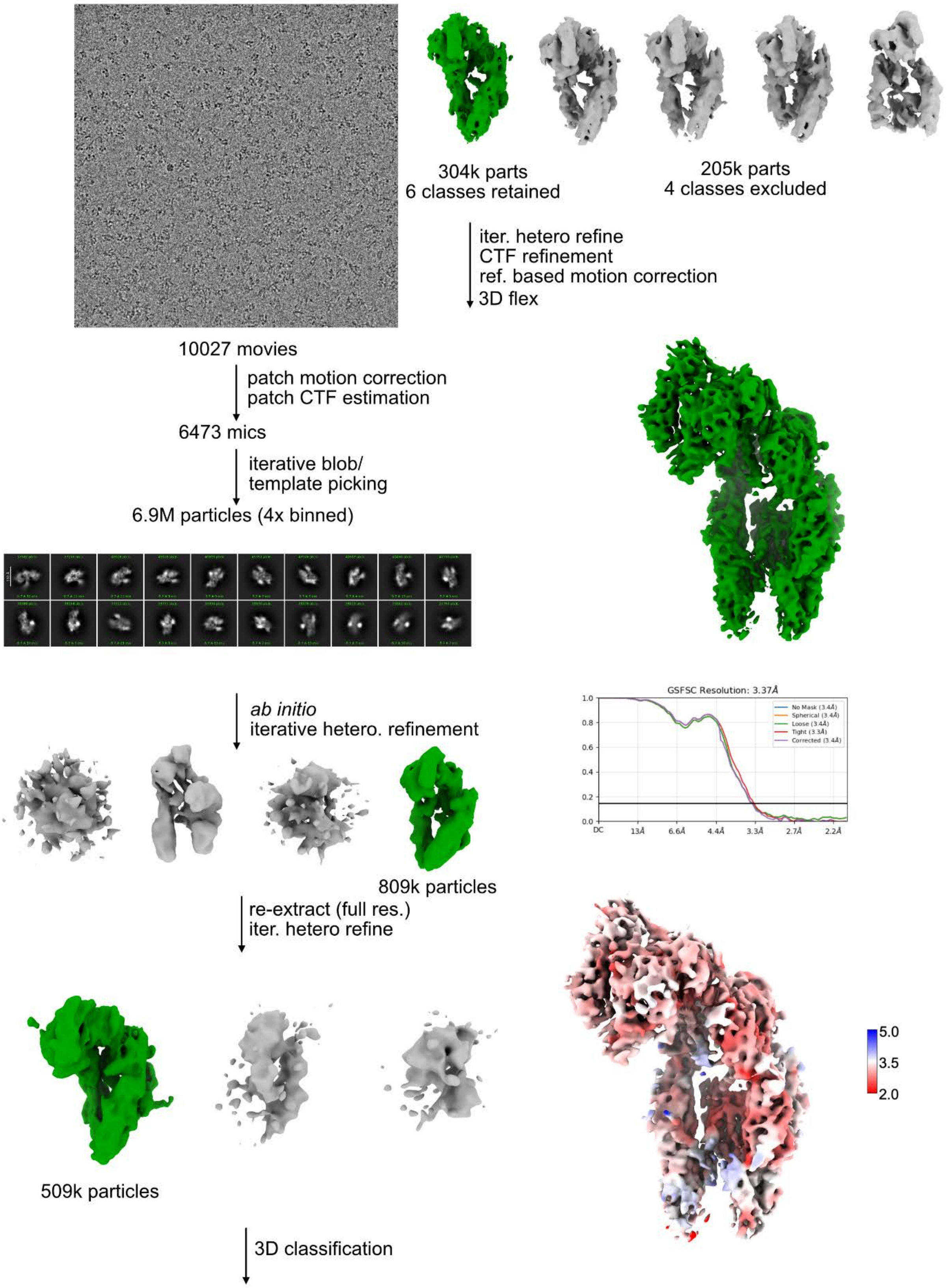

Fig. S6. CryoEM single particle analysis of 1_{Ins}-IGF-1R-zip complex dataset.

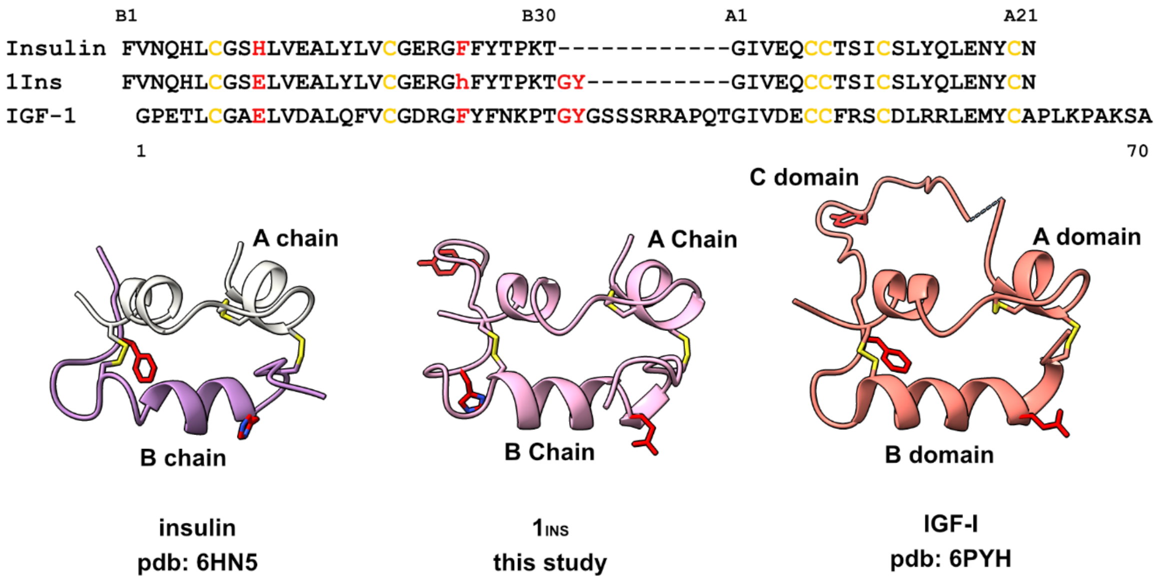

Fig. S7. Comparison of insulin, $\mathbf{1_{Ins}}$ and IGF-1 structures. Upper part: Comparison of the primary sequences of human insulin analog $\mathbf{1}_{\mathrm{Ins}}$ and human IGF-1. The invariant cystine residues are shown in orange. The positions mutated in $\mathbf{1}_{\mathrm{Ins}}$ and the corresponding amino acids in human insulin and IGF-1 are highlighted in red. h denotes *D*-His. **Lower part:** Main-chain representations of the receptor-bound structures of human insulin (PDB: 6HN5), $\mathbf{1}_{\mathrm{Ins}}$ (this study), and human IGF-1 (PDB: 6PXV). The A-chains of insulin and $\mathbf{1}_{\mathrm{Ins}}$ are shown in white, and the B-chains in violet. The IGF-1 chain is shown in coral. Disulfide bonds are depicted in yellow. Side chains of $\mathbf{1}_{\mathrm{Ins}}$ residues GluB10, D-HisB24, GlyB31, and TyrB32, and their corresponding counterparts in insulin and IGF-1, are shown as red sticks.

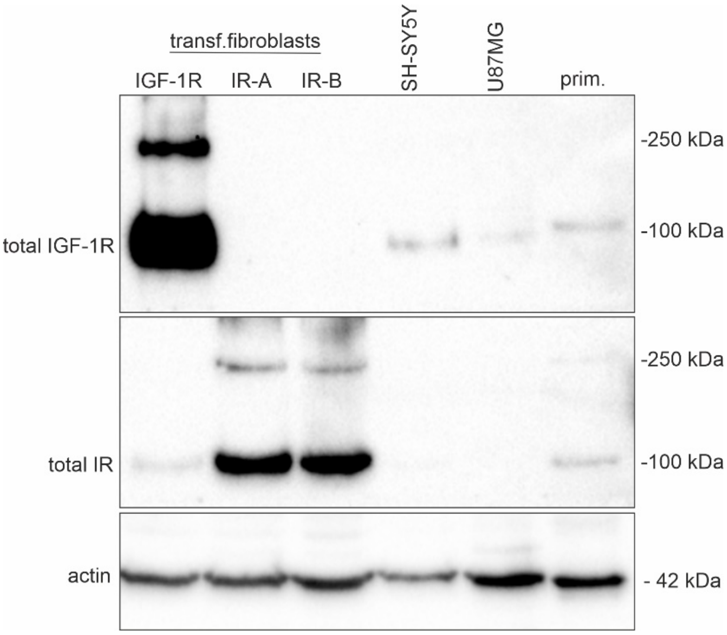

Fig. S8. Western blot showing total amount of IGF-1R and IR in different cellular models. The data were measured from specifically transfected fibroblasts (transf. fibroblasts) which are mouse fibroblasts derived from IGF-1R-knockout mice stably transfected with human receptors (IR-A, IR-B and IGF-1R), from human cell lines of neuronal origin i.e. the neuroblastoma SH-SY5Y cell line and the glioblastoma U87MG cell line, and primary cultures from neonatal rat brains (prim.). Membranes were cut at 75 kDa standards, and respective parts were developed with anti-Insulin Receptorβ (4B8) or with anti-IGF-1Rβ (111A9) antibodies (Mr above 75 kDa). Anti-actin (20-33) antibody (Mr below 50 kDa) was used as a loading control.

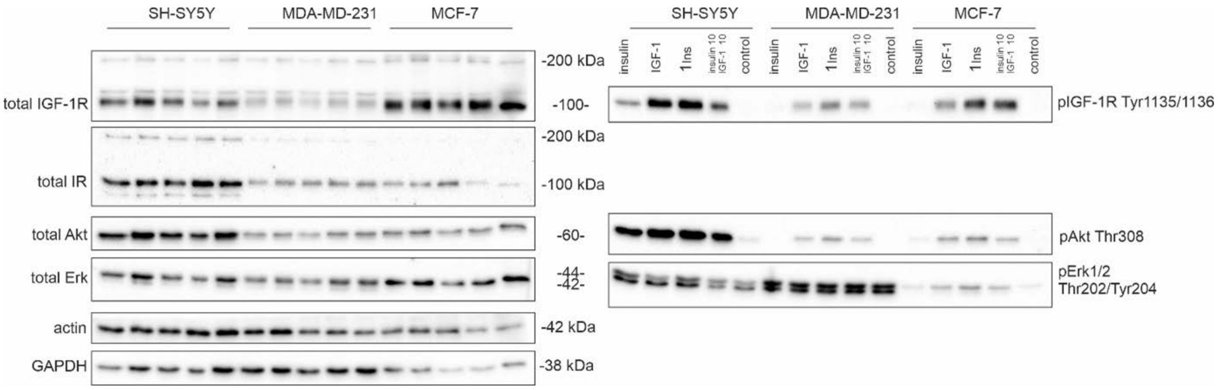

Fig. S9. Comparison of total protein expression in the SH-SY5Y, MDA-MD-231 and MCF-7 cell lines. Total protein expression in cell lines (~20 μg/well of cell lysates) and their stimulation with insulin, IGF-1, $\mathbf{1}_{Ins}$ and co-stimulation with insulin/IGF-1 (10 nM each).

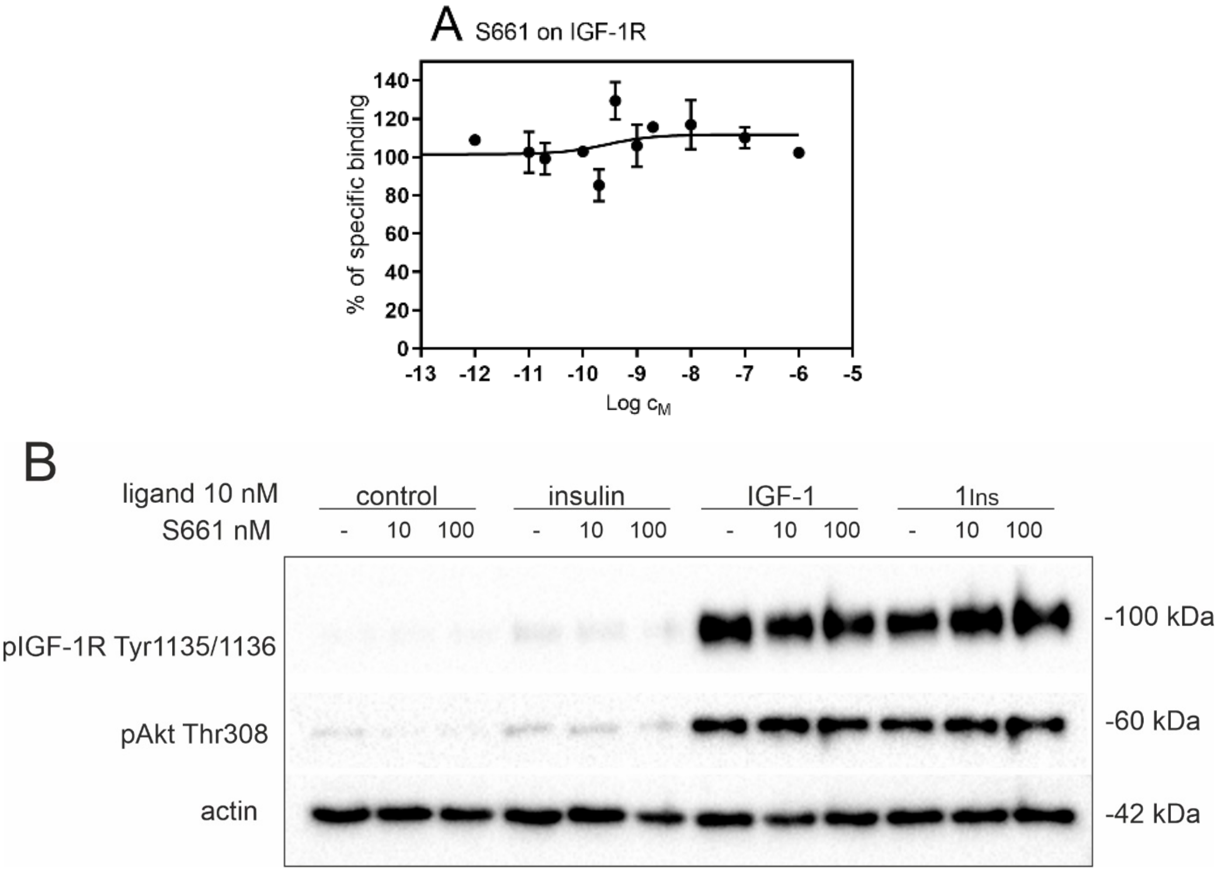

Fig. S10. Binding and signaling of the S661 peptide on IGF-1R. (**A**) Representative binding curve showing the inhibition by S661 of the binding of human ^{125}I–IGF-1 to IGF-1R in mouse fibroblasts transfected with the human receptor. (**B**) Western blot of IGF-1R–transfected mouse fibroblasts stimulated with 10 nM insulin, IGF-1, or $\mathbf{1}_{\mathrm{Ins}}$ in the presence of 10 or 100 nM S661 for 20 min.

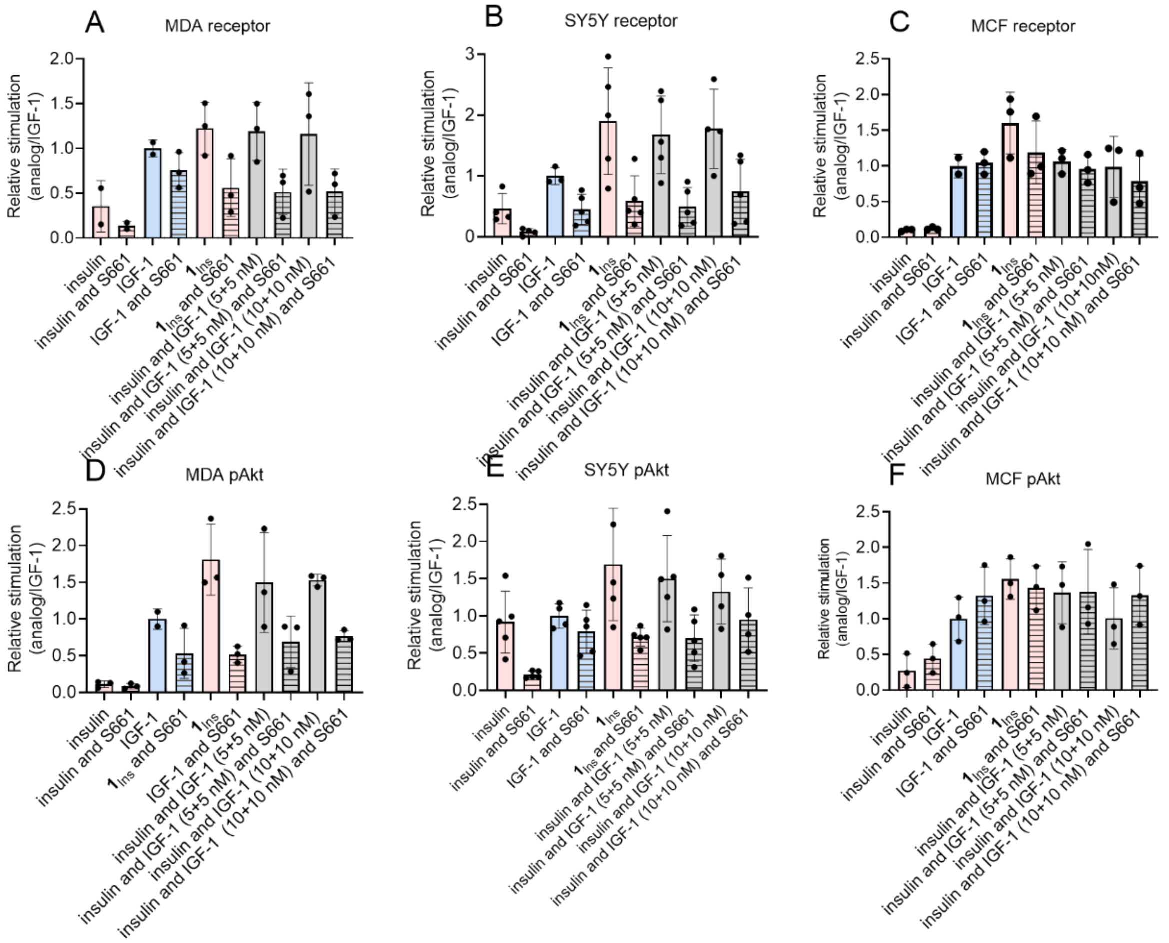

Fig. S11. Effect of S661 on the ability of the ligands to stimulate receptors (A-C) and Akt (D-F) in the MDA-MB-231 (A,D), SH-SY5Y (B,E), and MCF-7 (C,F) cell lines, respectively. The cells were stimulated with 10 nM insulin, IGF-1, $\mathbf{1}_{\mathrm{Ins}}$, or co-stimulated with insulin and IGF-1 (5 nM and 10 nM each) for 20 min. in presence or absence of 100 nM peptide S661. Phosphorylation of IR/IGF-1R and Akt was followed using Western blot (shown in *Source data for Fig. S11*). The data normalized to actin were expressed as the contribution of phosphorylation relative to the signal of IGF-1.

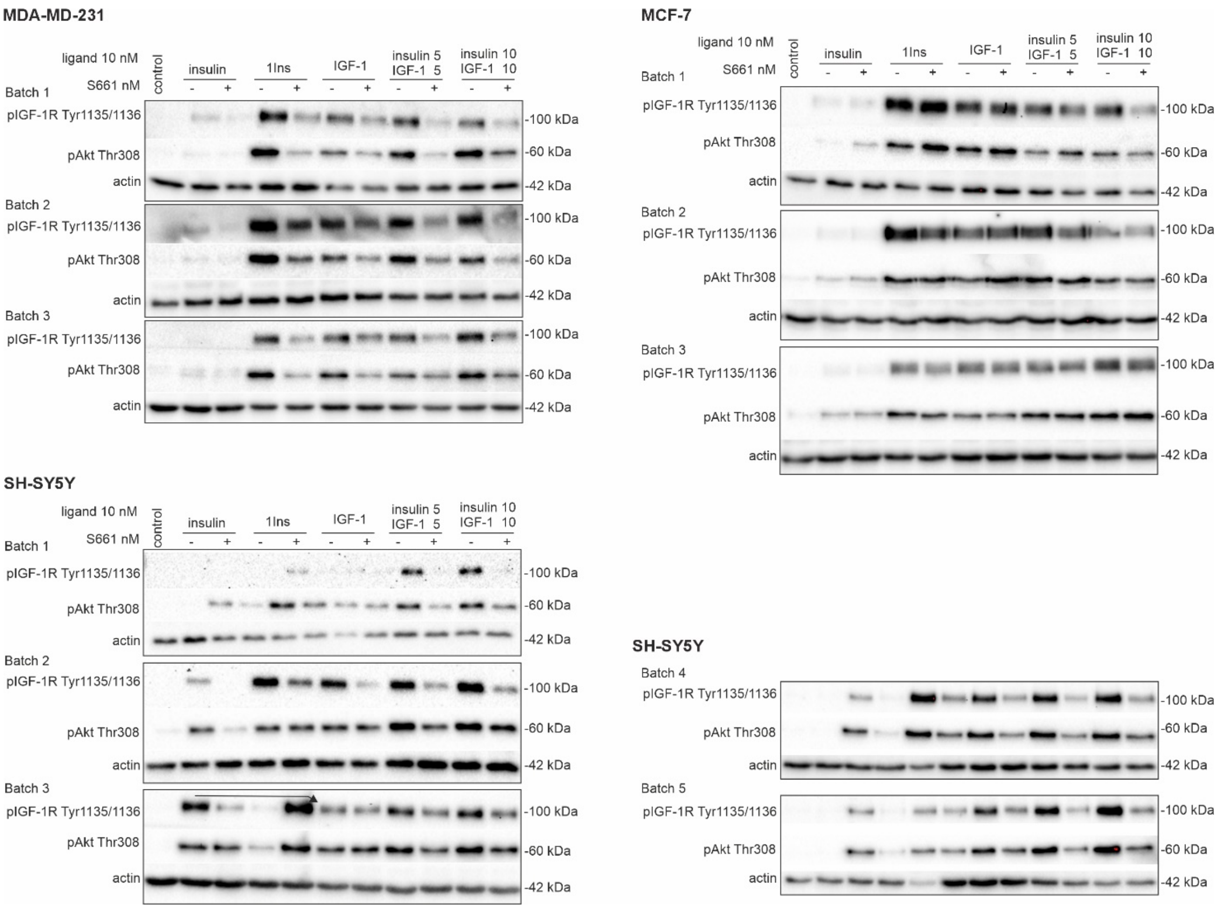

Source data for Fig. S11. Western blots showing stimulation of IGF-1R/IR and pAkt for 20 min by insulin, IGF-1, **1**$_{\mathrm{Ins}}$, or combination of insulin and IGF-1 (5 nM and 10 nM each) in the absence (–) or presence (+) of 100 nM S661 in the MDA-MB-231, SH-SY5Y, and MCF-7 cell lines. Membranes were cut at 75 kDa and 50 kDa standards, and respective parts were developed with anti-phospho-IGF-1Rβ (Tyr1135/1136)/IRβ (Tyr1150/1151) (Mr above 75 kDa), anti phospho-Akt (Thr308) (C31E5E) (Mr between 75 and 50 kDa) and actin (20-33) (Mr bellow 50 kDa) that was used as a loading control. 3-5 independent series of samples from different cell passages were prepared.

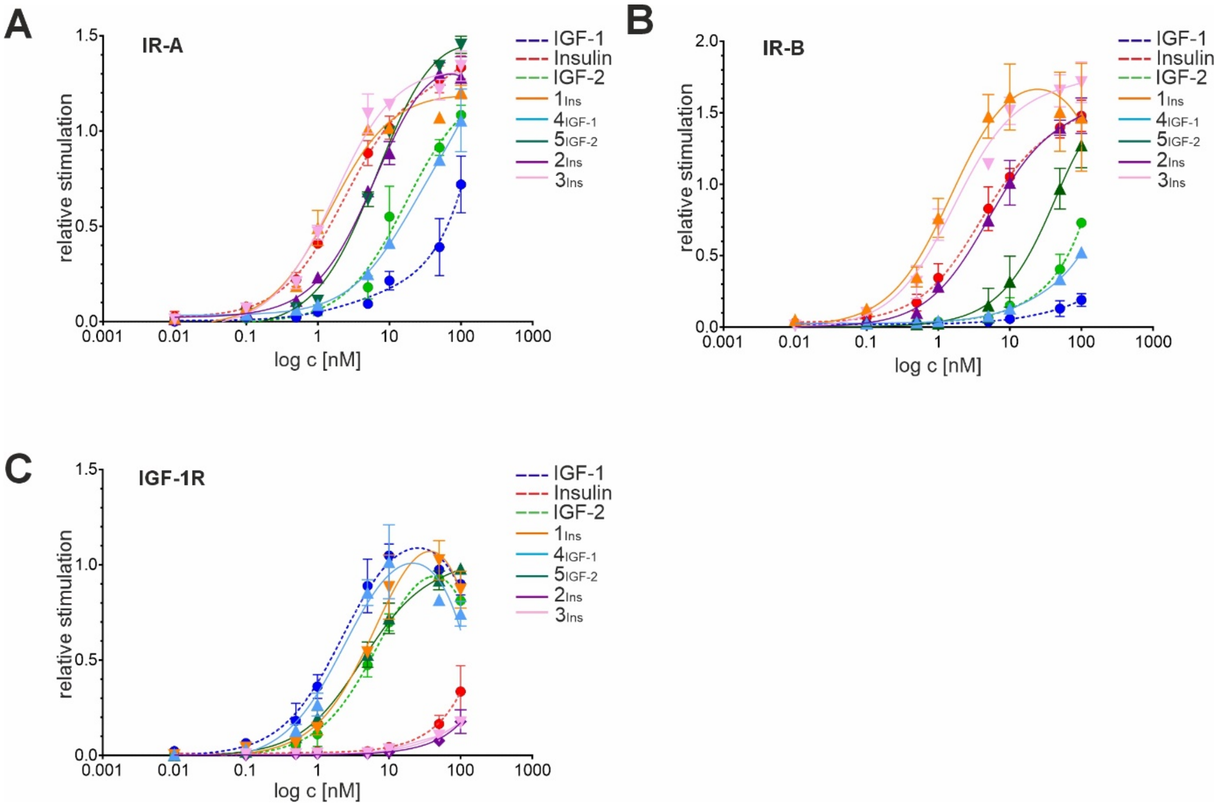

Fig. S12. Relative abilities of human insulin, IGF-1, IGF-2 and analogs to stimulate receptor phosphorylation determined by In-Cell Western assay. (**A**) IR-A-, (**B**) IR-B- and (**C**) IGF-1R-transfected fibroblasts were stimulated with 0.01 – 100 nM concentration range of wild-type ligands (dotted lines) and analogs (full lines). Data were obtained using In-Cell Western Assay (the plates are shown below in *Source data for Fig. S12*). The data (mean ± S.D., n ≥ 2) were expressed as the contribution of phosphorylation relative to the signal of human insulin (IR-A and IR-B) or IGF-1 (IGF-1R) at 10 nM. Nonlinear regression curve fitting of the combined data from all experiments was carried out with GraphPad Prism 8 software.

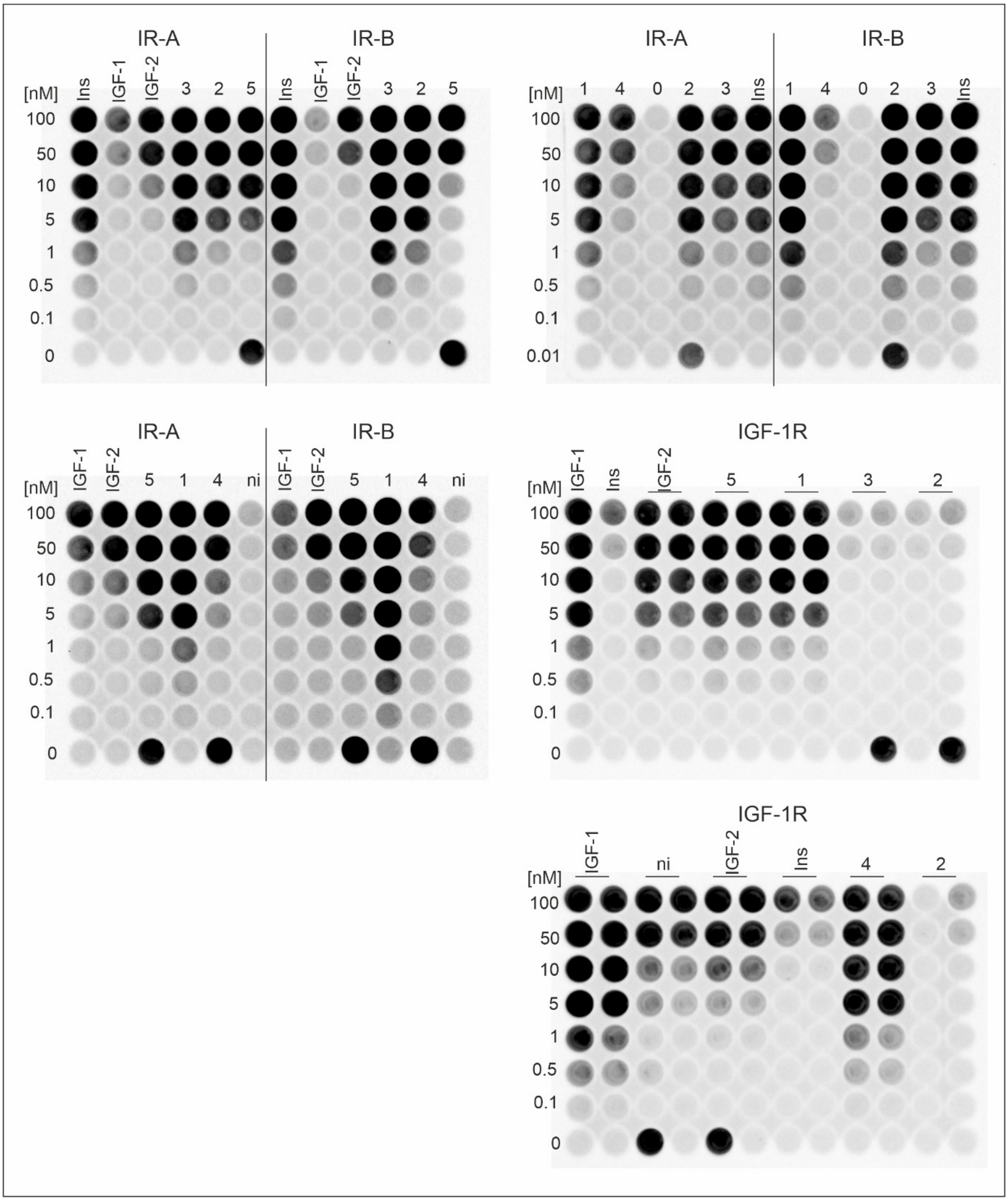

Source data for Fig. S12. Individual plates from In-Cell Western Assay. The mouse fibroblasts transfected with human IR-A, IR-B and IGF-1R were treated with the wt hormones and analogs (1 is $\mathbf{1}_{\text{Ins}}$, 2 is $\mathbf{2}_{\text{Ins}}$, 3 is $\mathbf{3}_{\text{Ins}}$, 4 is $\mathbf{4}_{\text{IGF-1}}$ and 5 is $\mathbf{5}_{\text{IGF-2}}$, ni analog not included in this study) at specified concentrations for 20 min. Formaldehyde fixed permeabilized cells were incubated with anti-phospho-IGF-1Rβ (Tyr1135/1136)/IRβ (Tyr1150/1151) and developed with peroxidase-labeled anti-rabbit secondary antibody (Sigma). SuperSignal West Femto maximum sensitivity substrate was added to each well, and chemiluminescence was detected using the ChemiDoc MP Imaging System. Data were subtracted from the background values (wells with no ligand) and expressed as the contribution of phosphorylation relative to the 10 nM insulin signal (IR-A and IR-B) or 10 nM IGF-1 signal (IGF-1R) (dark wells in the rows with no ligand).

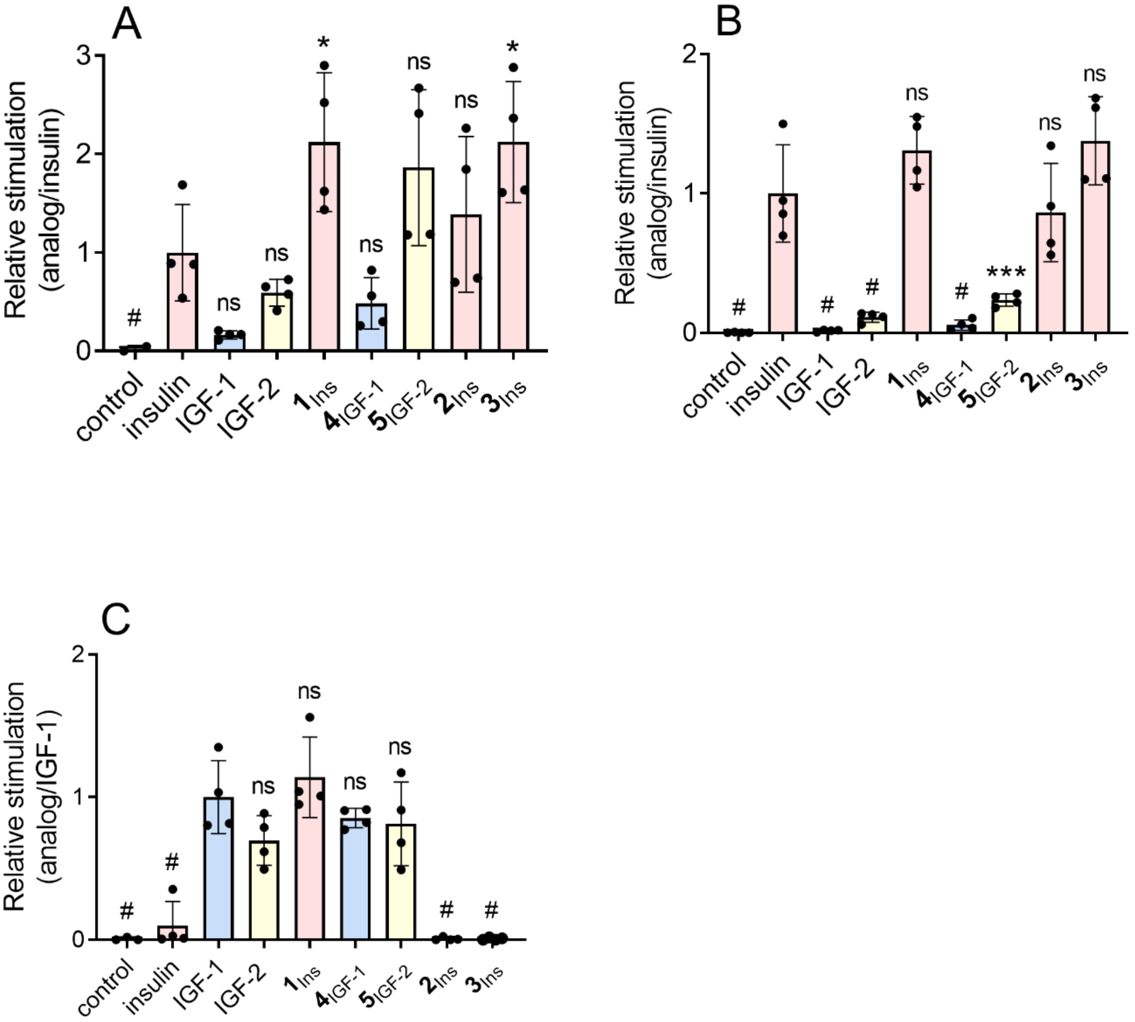

Fig. S13. Relative abilities of hormones and analogs to stimulate receptor autophosphorylation. Transfected fibroblasts with the human IR-A (**A**), IR-B (**B**) or IGF-1R (**C**) receptor were stimulated with 10 nM ligands for 20 min. Phosphorylation of IR and IGF-1R was followed using Western blot (shown in *Source data for Fig. 2, 3 and S13*). The data normalized to GAPDH were expressed as the contribution of phosphorylation relative to the signal of human insulin in IR-A and IR-B cells and relative to signal of IGF-1 in IGF-1R cells. Asterisks indicate that stimulation by the analog differs significantly from the stimulation by insulin or IGF-1, (ns = non-significant, * $p < 0.05$; ** $p < 0.01$; *** $p < 0.001$, # $p < 0.0001$).

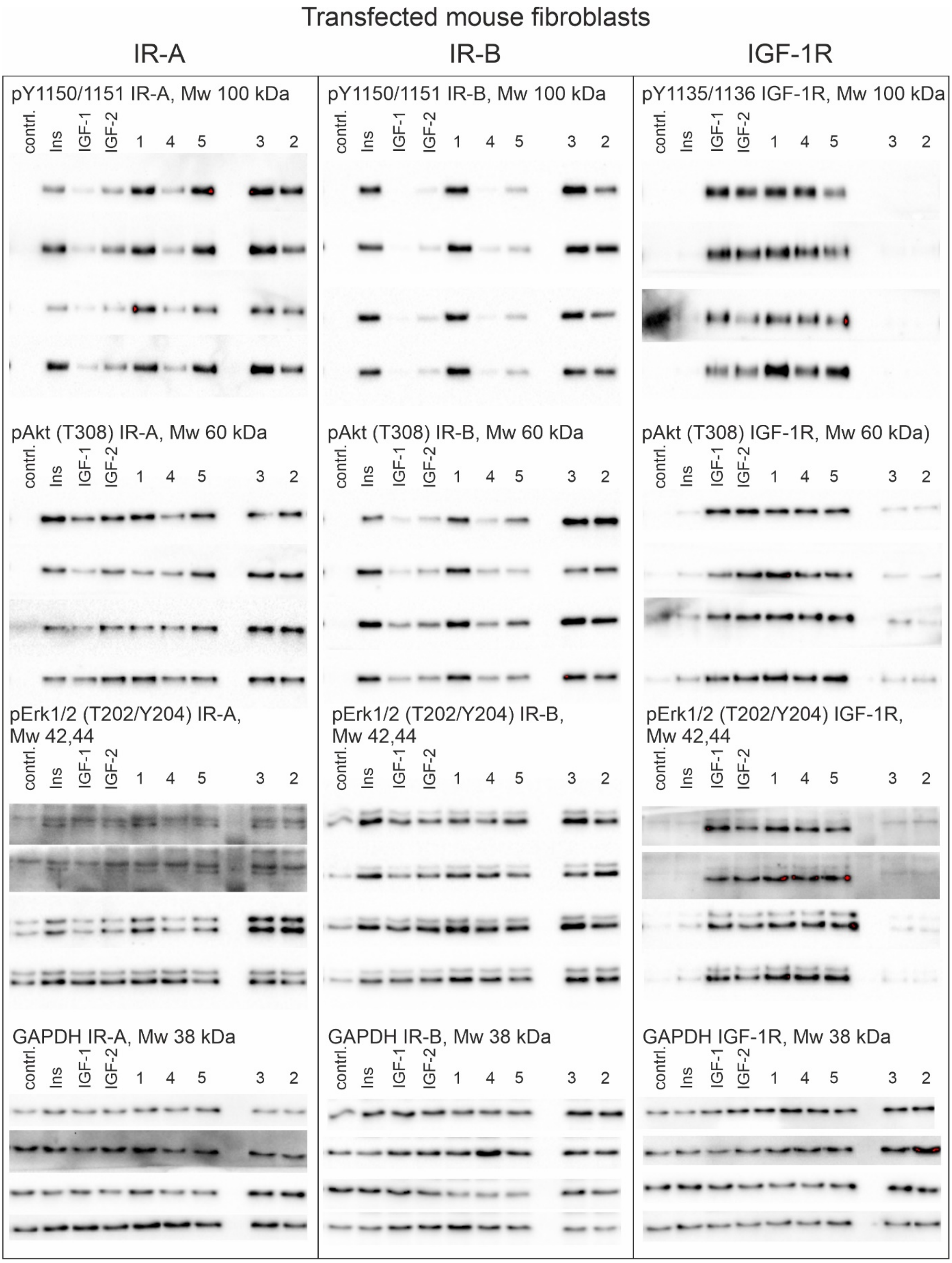

"Continued on the next page"

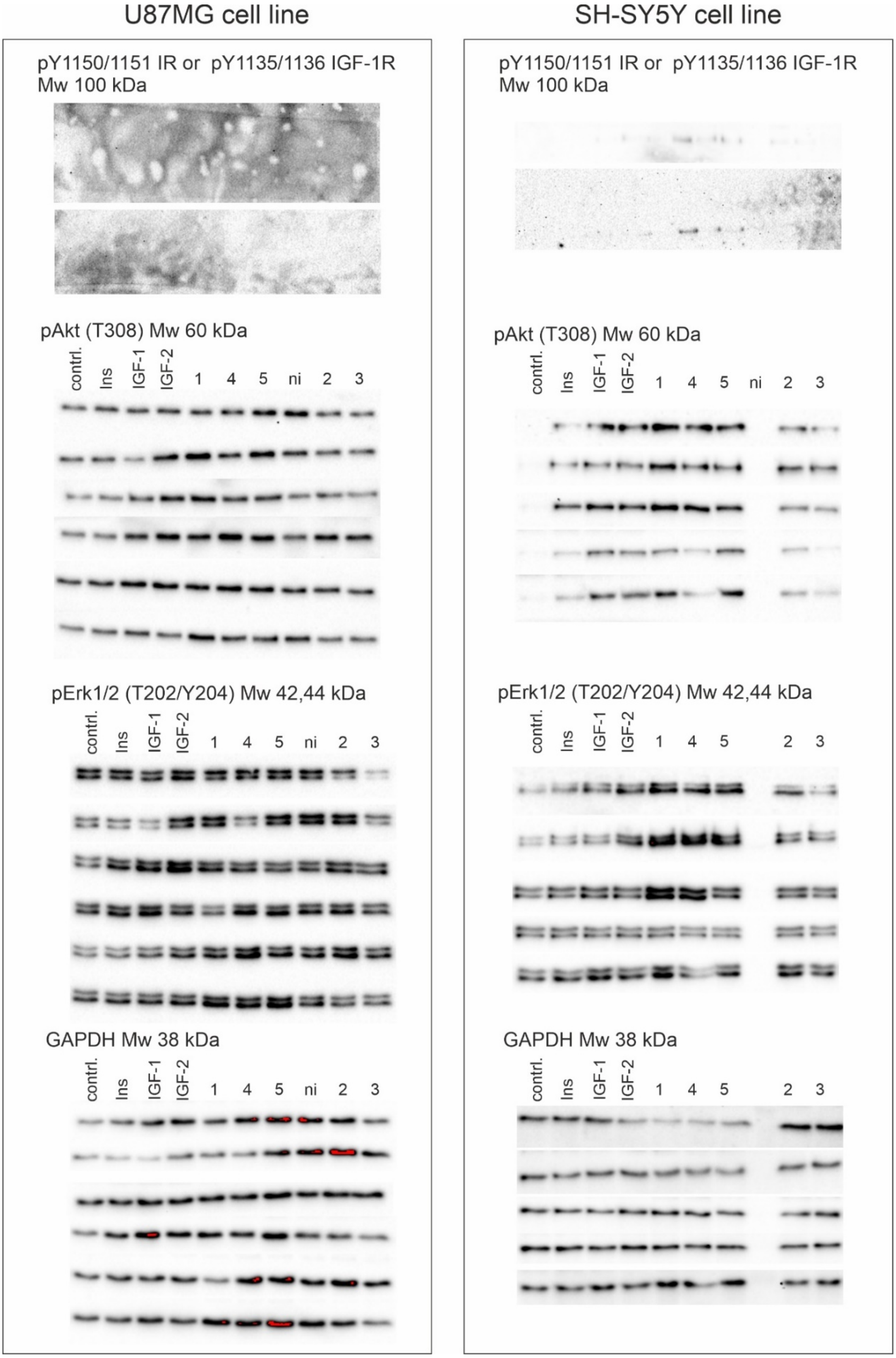

"Continued on the next page"

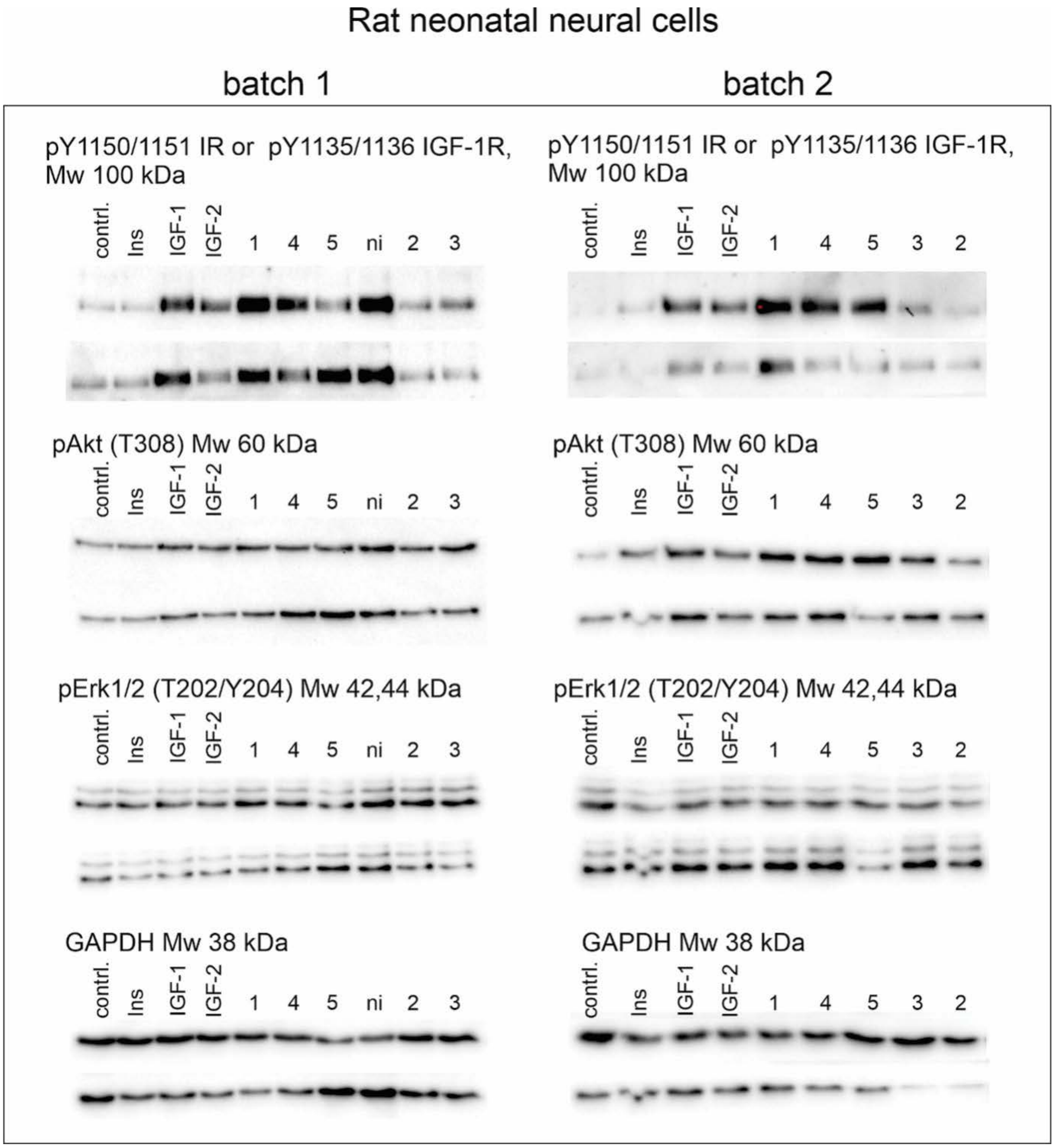

Source data for Fig. 2, 3 and S13. Western blots showing stimulation of IGF-1R/IR pathway for 20 min by wt hormones and analogs (1 is $\mathbf{1}_{Ins}$, 2 is $\mathbf{2}_{Ins}$, 3 is $\mathbf{3}_{Ins}$, 4 is $\mathbf{4}_{IGF\text{-}1}$ and 5 is $\mathbf{5}_{IGF\text{-}2}$, ni is for analog not included in this study) in mouse fibroblasts derived from IGF-1R knockout mice stably transfected with human receptors (IR-A, IR-B and IGF-1R), in human cell lines U87MG and SH-SY5Y and in rat postnatal cultures of neuronal cells. Membranes were cut at 75 kDa and 50 kDa standards, and respective parts were developed with anti-phospho-IGF-1Rβ (Tyr1135/1136)/IRβ (Tyr1150/1151) (Mr above 75 kDa), anti phospho-Akt (Thr308) (C31E5E) (Mr between 75 and 50 kDa) and phospho-p44/42 MAPK (Erk1/2) (Thr202/Tyr204) (Mr bellow 50 kDa). The membrane strip after phosphor-Erk visualization was re-stained with anti-GAPDH (D4C6R) antibody used as a loading control. 4-6 independent series of samples from different cell passages were prepared and are shown for each type of receptor and antibody. Primary cells were stimulated in duplicates and two technical replicates of each blot were prepared.

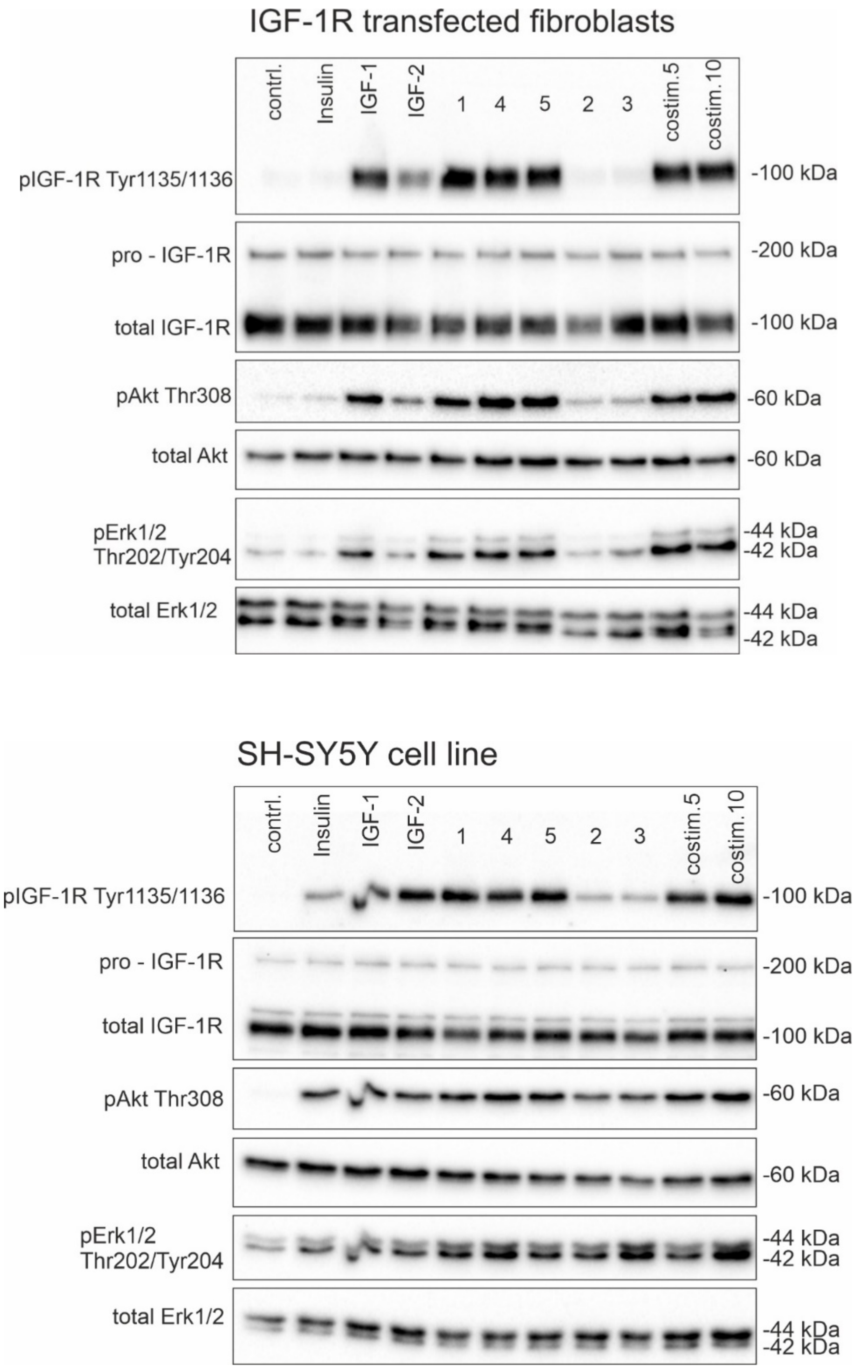

Fig. S14. Western blots showing total proteins in IGF-1R transfected fibroblasts and SH-SY5Y cells. Western blots are shown after stimulation of the IGF-1R/IR signaling pathway for 20 min by native hormones and analogs (1 = $\mathbf{1}_{\text{Ins}}$, 2 = $\mathbf{2}_{\text{Ins}}$, 3 = $\mathbf{3}_{\text{Ins}}$, 4 = $\mathbf{4}_{\text{Ins}}$, 5 = $\mathbf{5}_{\text{IGF-2}}$ and co-stimulated with insulin and IGF-1, costim.5 = 5 nM each and costim.10 = 10 nM each) in mouse fibroblasts derived from IGF-1R-knockout mice stably transfected with human IGF-1R, and in the human cell line SH-SY5Y. Samples were run in parallel on two gels. Membranes were cut at the 75 kDa and 50 kDa molecular weight markers, and the respective parts were probed with the following antibodies: anti-phospho-IGF-1Rβ (Tyr1135/1136)/IRβ (Tyr1150/1151) or IGF-1 Receptor β (111A9) (Mr > 75 kDa); anti-phospho-Akt (Thr308) (C31E5E) or Akt (pan) (C67E7) (Mr between 75 and 50 kDa); and anti-phospho-p44/42 MAPK (Erk1/2) (Thr202/Tyr204) or p44/42 MAPK (Erk1/2) (3A7) (Mr below 50 kDa).

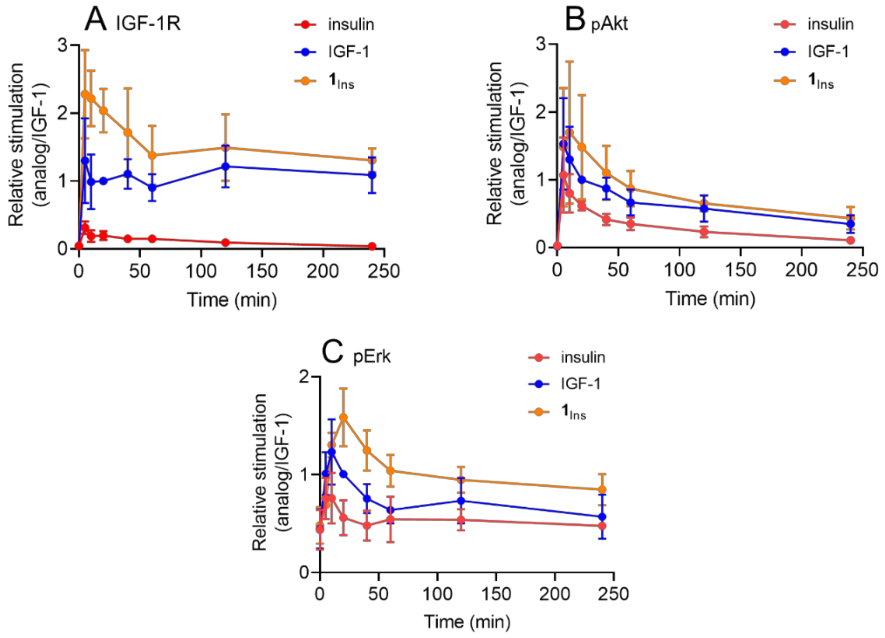

Fig. S15. Time profile of stimulation of IGF-1R receptor autophosphorylation (A) and phosphorylation of Akt (B) and Erk1/2 (C) in SH-SY5Y cell line. Cells were stimulated for indicated time points with 10 nM insulin (red), IGF-1 (blue) or $\mathbf{1}_{Ins}$ (orange). Densities were normalized to GAPDH and related to the signal of IGF-1 at 20 min.

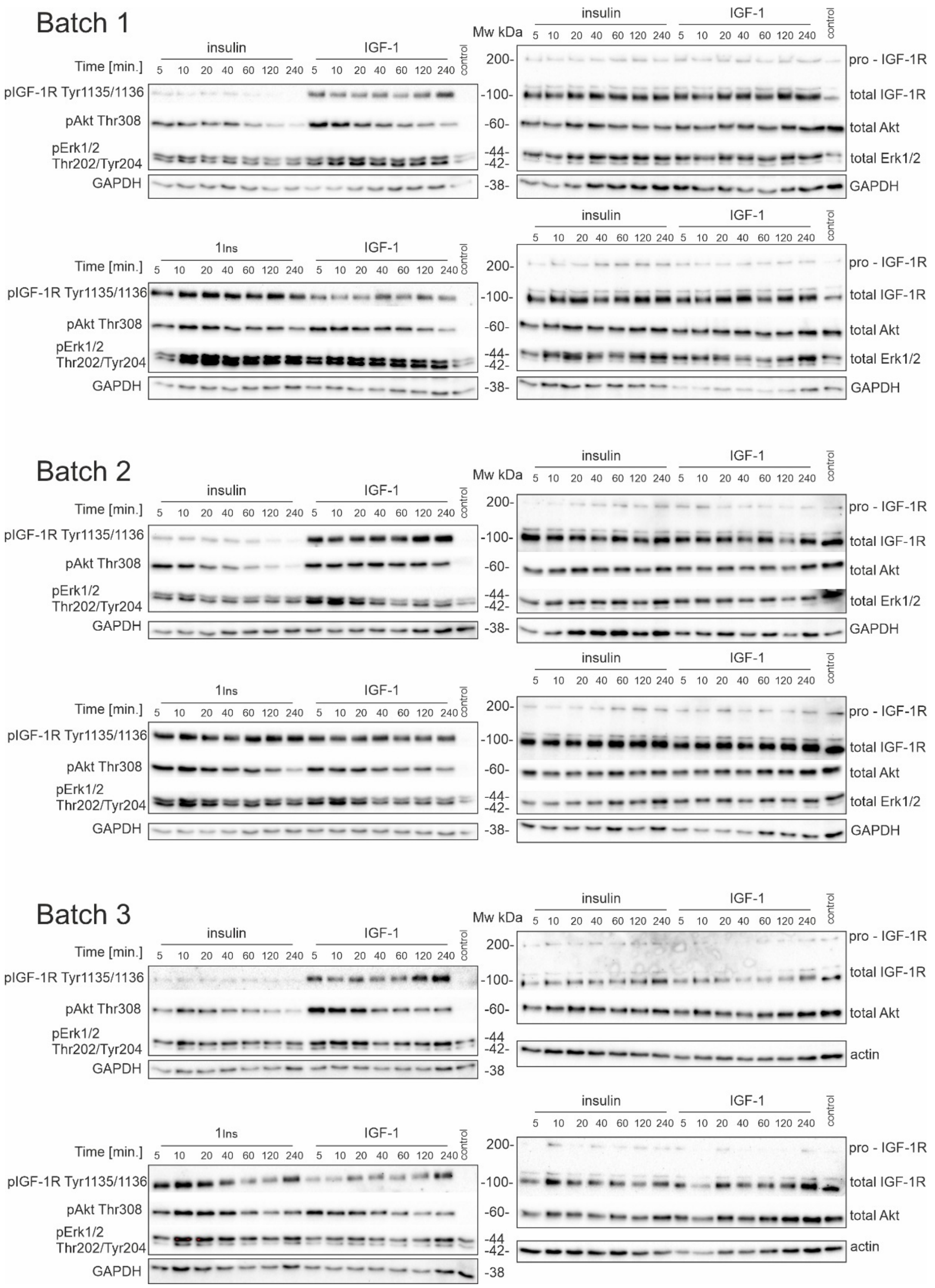

Source data for Fig. S15. Western blots showing time profile of stimulation IGF-1R receptor autophosphorylation, phosphorylation of Akt and Erk1/2 in SH-SY5Y cell line Two gels were run in parallel and electroblotted by standard procedure. Membranes were cut at 75 kDa and 50 kDa standards, and respective parts were developed with anti-phospho-IGF-1Rβ (Tyr1135/1136)/IRβ (Tyr1150/1151) or IGF-1 Receptor β (111A9) (Mr above 75 kDa), anti phospho-Akt (Thr308) (C31E5E) or Akt (pan) (C67E7) (Mr between 75 and 50 kDa) and phospho-p44/42 MAPK (Erk1/2) (Thr202/Tyr204) or p44/42 MAPK (Erk1/2) (3A7) or actin (20-33) (Mr bellow 50 kDa) . The membrane strip after phosphor-Erk visualization was re-stained with anti-GAPDH (D4C6R) antibody used as a loading control . Three independent series of samples from different cell passages were prepared.

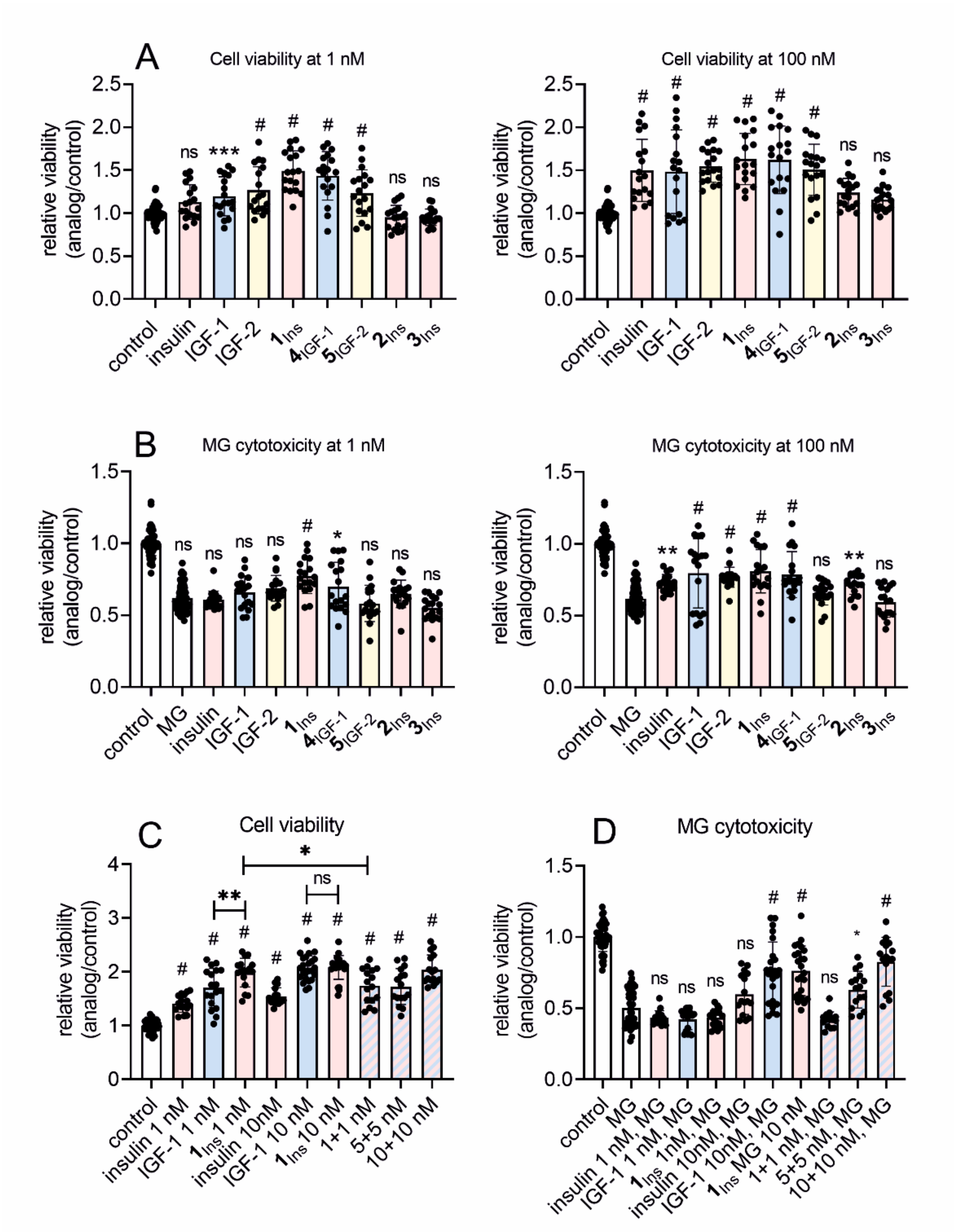

Fig. S16. Viability and survival of SH-SY5Y cells. (**A**) The cells were treated with human insulin, IGF-1, IGF-2 and analogs at 1 nM (left) or 100 nM (right) in medium without serum for 20 hours. (**B**) The cells were treated with the ligands as in (**A**) but in the presence of 1.5 mM methylglyoxal (MG) for 16 hours. (**C**) The cells were co-stimulated with IGF-1 and insulin (1, 5 or 10 nM each) and compared with 10 nM or 1 nM insulin, IGF-1 or **1**$_{Ins}$ and (**D**) treated with MG as in (**B**). The relative number of living cells was determined using MTT test was related to cells without treatment (control) in (**A, C**) or to cells treated with MG alone in (**B, D**). Asterisks indicate that number of living cells after ligand treatment differs significantly from control (in **A** and **C**) or MG (in **B** and **D**) in ANOVA analysis (ns = non-significant, * p < 0.05; ** p < 0.01; *** p < 0.001, # p < 0.0001). Significance in changes between **1**$_{Ins}$ and IGF-1 and combination of insulin and IGF-1 (1+1 nM) in (C) was calculated using t-test.

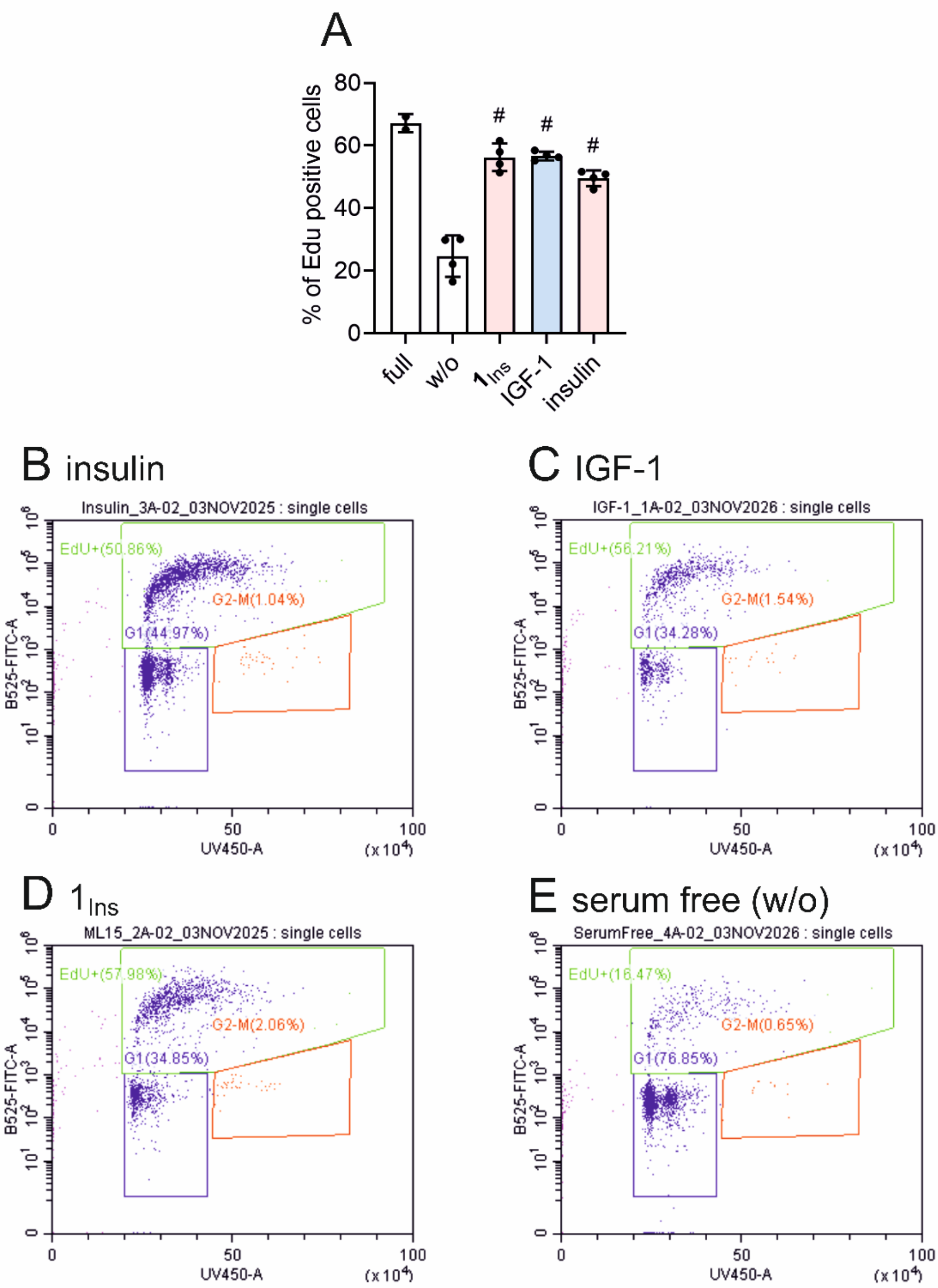

Fig. S17. Proliferation of SH-SY5Y assessed by EdU incorporation into the newly synthesized DNA. (A) EdU positive cells after 24 hours incubation with 10 nM $\mathbf{1}_{\mathrm{Ins}}$, IGF-1 or insulin compared to cells incubated in media without serum (w/o) and in full media (full). Representative cytograms of cells stimulated with insulin (**B**), IGF-1 (**C**) and $\mathbf{1}_{\mathrm{Ins}}$ (**D**) compared to unstimulated cells (w/o, **E**). Alexa Fluor 488 fluorescence (EdU signal) was detected using the B525 (FITC) channel and DAPI fluorescence (DNA content) was detected using the UV450 channel. EdU positive cells and cells in G1 and G2 phase are shown. In (**A**), the effects of ligand treatment differ significantly from the serum-free control (w/o) (# $p < 0.0001$).

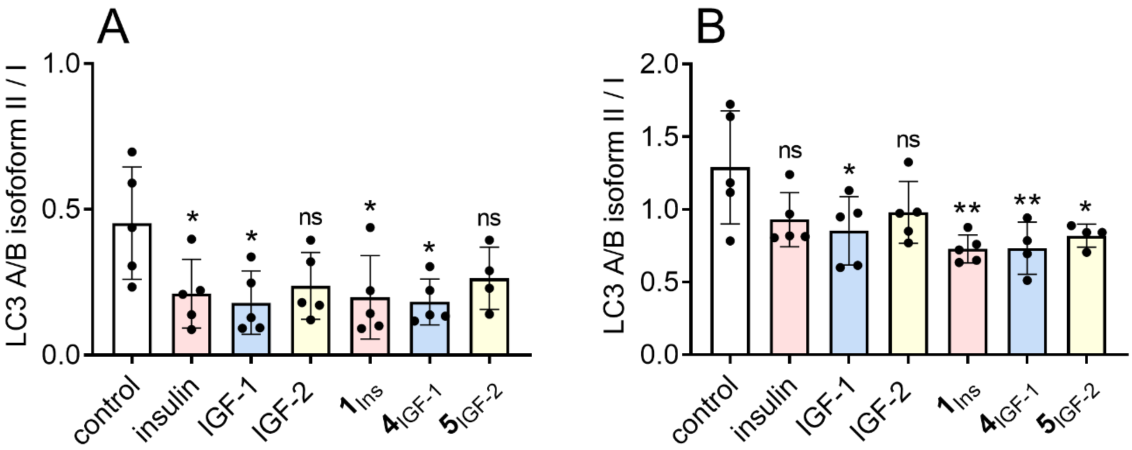

Fig. S18. Changes in the ratio of autophagy markers LC3A/B isoforms I and II in SH-SY5Y cells. The cells were treated with the ligands for 2 hours and then 0.5 mM MG was added for another 2 hours. Ratio of isoforms of LC3 A/B between cells treated with ligands alone (**A**) and with MG (**B**) are shown in arbitrary units. Asterisks indicate that change in the ratio of LC3 A/B isoforms induced by the analog differs significantly from the control cells (ns = non-significant, * p < 0.05; ** p < 0.01). Blots are shown in *Source data for Fig. S18*).

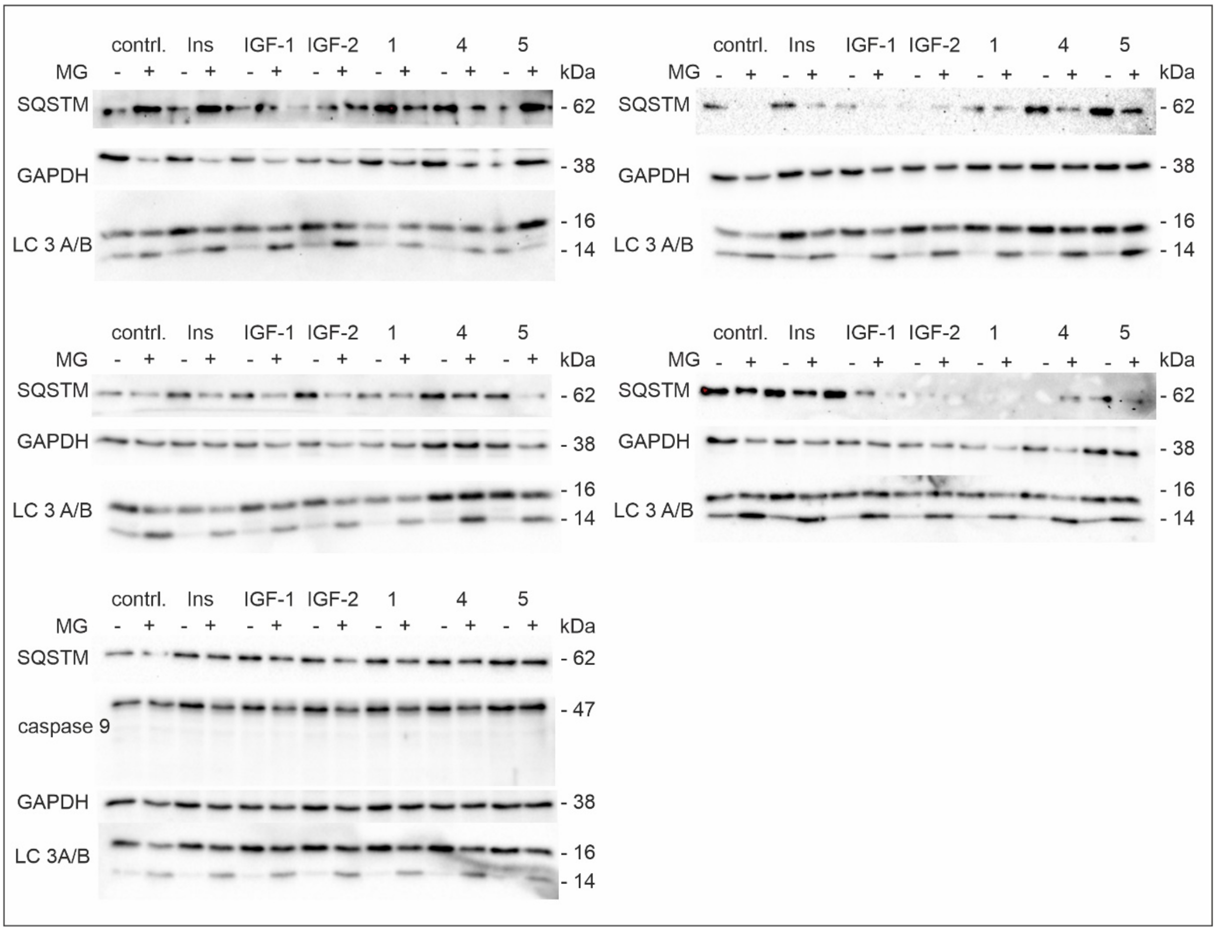

Source data for Fig. S18. Western blots showing changes in autophagy markers in SH-SY5Y cells after treatment with wt hormones and promiscuous analogs for 4h (1 is $\mathbf{1}_{\mathrm{Ins}}$, 4 is $\mathbf{4}_{\mathrm{IGF\text{-}1}}$ and 5 is $\mathbf{5}_{\mathrm{IGF\text{-}2}}$) and stressed by methylglyoxal (MG) for 2h. Membranes were cut at 75 kDa, 50 kDa and 25 kDa standards, and respective parts were developed with SQSTM1/p62 (Mr between 75 and 50 kDa), anti-GAPDH (D4C6R) antibody used as a loading control or/and with Caspase-9 (C9) (Mr between 50 and 25 kDa) and LCA/B (D3U4C) (Mr bellow 25 kDa). Cells were stimulated five times.

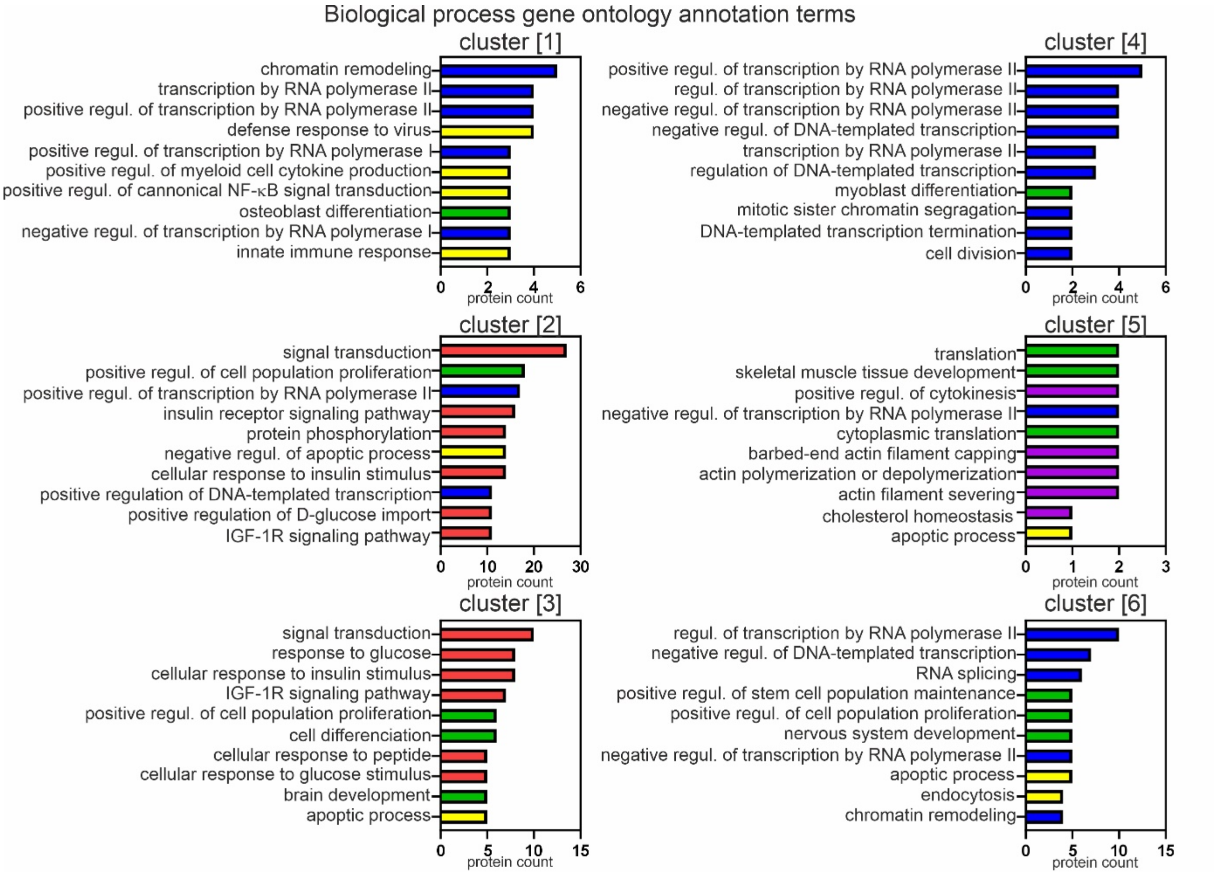

Fig. S19. Biological processes associated with altered phosphorylation in SH-SY5Y cells following ligand stimulation. Gene Ontology (GO) terms associated with proteins with phosphosites differentially regulated by IGF-1, $\mathbf{1}_{\mathrm{Ins}}$, and insulin. Upregulated phosphosites were grouped into three clusters: [1] IGF-1 > $\mathbf{1}_{\mathrm{Ins}}$ ≈ insulin, [2] IGF-1 ≈ $\mathbf{1}_{\mathrm{Ins}}$ > insulin, and [3] $\mathbf{1}_{\mathrm{Ins}}$ > IGF-1 ≈ insulin. Downregulated phosphosites were assigned to clusters: [4] $\mathbf{1}_{\mathrm{Ins}}$ > IGF-1 ≈ insulin, [5] IGF-1 > $\mathbf{1}_{\mathrm{Ins}}$ ≈ insulin, and [6] $\mathbf{1}_{\mathrm{Ins}}$ ≈ insulin > IGF-1. GO terms associated with *transcription and cell division* are shown in blue, *insulin/IGF signaling* in red, *proliferation* in green, *apoptosis and stress response* in yellow, and *actin cytoskeleton organization* in violet. Note the varying number of proteins represented in each cluster. Data correspond to main text Fig. 6B.

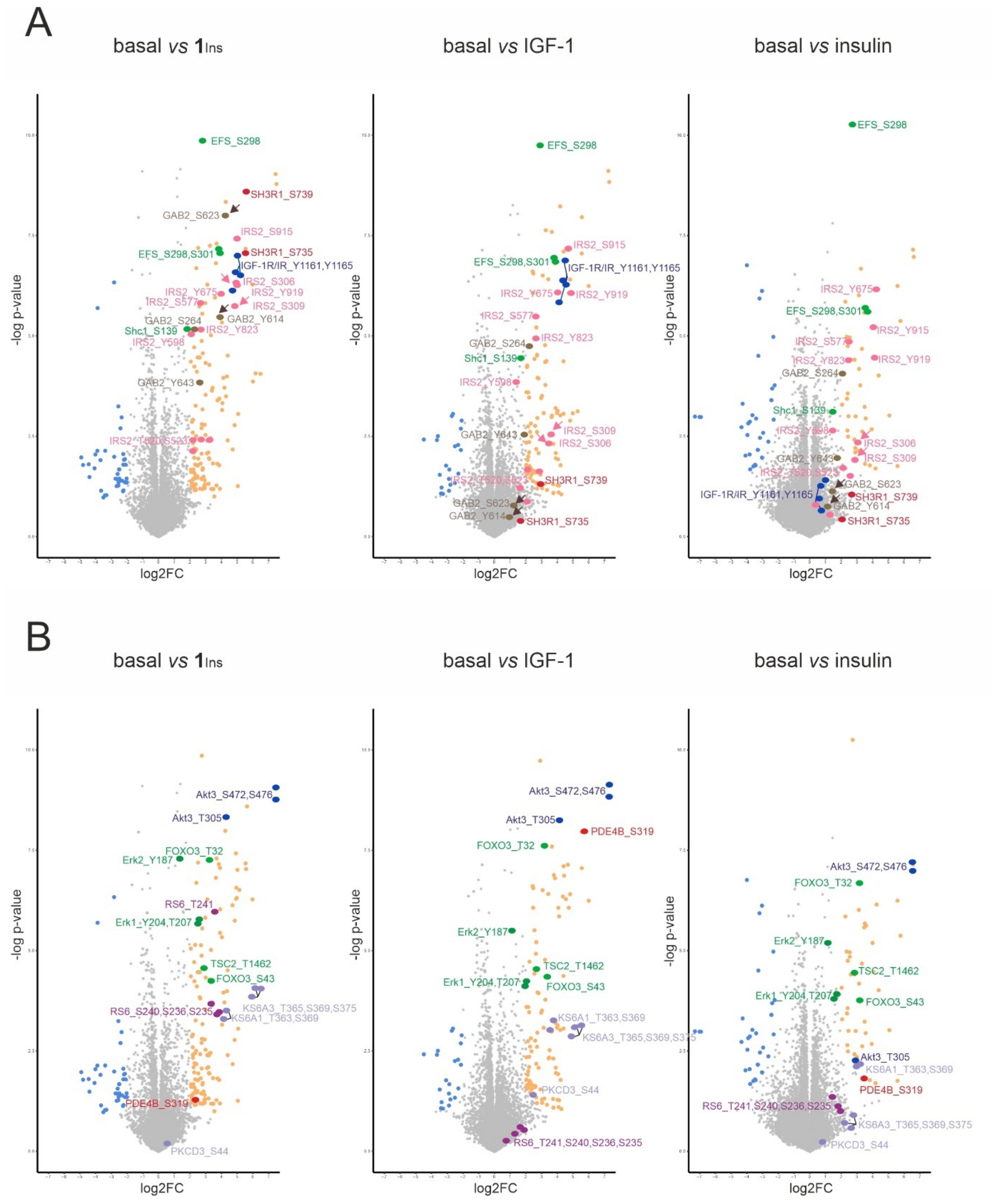

Fig. S20. Volcano plots of phosphosites altered between basal and ligand-stimulated (10 nM for 15 min) SH-SY5Y cells based on fold changes (FC). Small orange dots represent phosphosites significantly upregulated upon treatment, while light blue dots indicate phosphosites significantly downregulated (± 4-fold with FDR < 0.15). (**A**) Phosphosites corresponding to receptor phosphorylation (blue) and adaptor proteins: IRS2 (pink), GAB2 (brown), EFS and Shc1 (green), and SH3R1 (red). Arrows indicate phosphosites with substantial changes between the treatments. (**B**) Phosphosites corresponding to downstream kinases and regulatory proteins: Akt (blue), Erk1/2, FOXO3, and TSC2 (green), RSKs and PKC (light violet), and PDE4B (red).

Table S1. Binding affinities of native hormones and analogs. Binding affinities for IR-A were determined using IM-9 cells and affinities for IR-B and IGF-1R using mouse fibroblasts transfected with the human receptors. Binding affinities for the IGF-2R domain 11 (D11) and IGFBP-3 were determined using isolated proteins attached to plates. The data were adopted from published papers together with original values for wt hormones in respective measurements (marked by superscripts) [a] Ref. (*21*), [b] Ref. (*22*), [c] Ref. (*20*), [d] determined in this study, [e] Ref. (*24*). Each K_d ± S.E. [nM] represents a mean of (n) independent measurements. nb is no binding at 10^{-6} M, n.d. is not determined.

		K_d ± S.E. [nM] (n) for				
	Analog	IR-A	IR-B	IGF-1R	D11 of IGF-2R	IGFBP-3
insulin		0.43 ± 0.01 (5)[a]<br>0.25 ± 0.05 (5)[b1]<br>0.32 ± 0.09 (4)[b2]<br>0.43 ± 0.09 (4)[c]<br>0.46 ± 0.06 (4)[d]	0.67 ± 0.08 (4)[a]<br>0.50 ± 0.31(5)[b]<br>0.49 ± 0.18 (3)[c]<br>0.38 ± 0.12 (4)[d]	292 ± 31(3)[a]	n.d.	nb[d]
IGF-1		23.7 ± 11.5 (3)[b1]	224 ± 16 (4)[b]	0.24 ± 0.05 (5)[a]<br>0.34 ± 0.12 (4)[b1]<br>0.16 ± 0.06 (3)[b2]<br>0.25 ± 0.03 (4)[b3]<br>0.30 ± 0.10 (4)[d]<br>0.15 ± 0.01 (3)[d1]	nb[e]	0.31 ± 0.07 (3)[e]
IGF-2		2.9 ± 0.2 (3)[b1]	35.5 ± 5.6 (4)[b]	2.3 ± 1.2 (3)[b3]	1.29 ± 0.27 (7)[e]	0.2 ± 0.1 (4)[e]
**1**$_{Ins}$	[GluB10, D-HisB24, GlyB31, TyrB32]-insulin	0.22 ± 0.06 (3)[d]	0.04 ± 0.01(4)[d]	0.38 ± 0.12 (5)[d]<br>0.18 ± 0.01 (2)[d1]	nb[d]	nb[d]
**2**$_{Ins}$	[AlaB29, GluB31, amideB31]-insulin	0.97 ± 0.55 (4)[c]	0.35 ± 0.04 (3)[c]	nb	nb[d]	nd[d]
**3**$_{Ins}$	[Cyclo(Nva(δN_3)B26-PrgB29)]-insulin	0.19 ± 0.02 (3)[a]	0.13 ± 0.02 (3)[a]	1327 ± 461 (3)[a]	nb[d]	nb[d]
**4**$_{IGF-1}$	[His49]-IGF-1	6.7 ± 2.4 (3)[b2]	72.3 ± 12.0 (3)[b]	0.50 ± 0.23 (4)[b1]	nb[d]	4.6 ± 1.1 (3)[d]
**5**$_{IGF-2}$	[His48]-IGF-2	0.54 ± 0.13 (3)[b2]	4.3 ± 1.7 (4)[b]	0.88 ± 0.23 (3)[b2]	2.58 ± 0.18 (3)[d]	0.29 ± 0.16 (3)[d]

Table S2. Cryo-EM data collection, refinement and validation statistics.

	**1**$_{Ins}$-IR-zip (EMDB-49260) (PDB 9NCN)	**1**$_{Ins}$-IGF-1R-zip (EMDB-49261) (PDB 9NCO)
**Data collection and processing**		
Magnification	105,000	75,000
Voltage (kV)	300	-
Electron exposure (e–/Å^2)	50	-
Defocus range (μm)	-0.5 – 2.0	-
Pixel size (Å)	0.833	1.036
Symmetry imposed	C2	-
Initial particle images (no.)	6.9 M	4 M
Final particle images (no.)	166k	352k
Map resolution (Å)	2.86	3.4
FSC threshold	0.143	0.143
Map resolution range (Å)	1.8 – 39	2.3-7.3
**Refinement**		
Initial model used (PDB code)	6PXV	8TAN
Model resolution (Å)	2.8/2.9/3.3	2.9/3.3/4.0
FSC threshold	(0/0.143/0.5)	(0/0.143/0.5)
Model resolution range (Å)	2.8 – 3.4	2.9 – 4.1
Map sharpening *B* factor (Å^2)	unsharpened	unsharpened
Model composition		
Non-hydrogen atoms	10733	13605
Protein residues	1306	1693
Ligands	12	1
B factors (Å^2)	min/max/mean	min/max/mean
Protein	20.54/223.31/107.86	44.64/313.61/146.84
Ligand	78.36/154.40/114.51	101.55/101.55/101.55
R.m.s. deviations		
Bond lengths (Å)	0.003 (0)	0.004 (0)
Bond angles (°)	0.742 (1)	0.981 (0)
Validation		
MolProbity score	1.34	1.71
Clashscore	0.9	1.68
Poor rotamers (%)	1.42	2.19
Ramachandran plot		
Favored (%)	92.86	90.76
Allowed (%)	6.99	9.12
Disallowed (%)	0.16	0.12
Model vs. Data		
CC (mask)	0.76	0.71
CC (box)	0.81	0.80
CC (peaks)	0.66	0.65
CC (volume)	0.74	0.71
Mean CC (ligands)	0.76	0.71

Table S3. Predicted ratios of receptor forms in individual cell lines. Predictions are based on the mRNA expression of the receptors and the theory of random assembly. The mRNA amount was related to the actin expression in each cell line and expressed as % of actin mean ± S.D. (n = 4). The homodimers and hybrid receptors are estimated as (%) from sum of all receptors in a particular cell line.

Cell line	Mouse IR-A mRNA	Human IR-A mRNA	Human IR-B mRNA	Human IGF-1R mRNA	IR-A (%)[a] homodimer	IGF-1R (%)[a] homodimer	IR-A/IGF-1R (%)[a] hybrid
IR-A	0.76±0.46	9.60±8.06	-	-	100	-	-
IR-B	0.76±0.46	-	15.99±5.62	-			
IGF-1R	0.66±0.20	-	-	202±30	-	99.6	0.4
SHSY5Y		0.44±0.05	-	2.45±0.44	2.3	71.9	25.8
U87MG		0.03	0.05	0.65±0.07	1.2	79.3	19.5
Mouse brain	Mouse IR-A 16.60±1.35		Mouse IR-B 1.30±0.24	Mouse IGF-1R 41.21±6.43	8.5[b]	51.4	40.1

[a] The theory of random assembly (*29*) says that if total concentrations of IR and IGF-1R subunits are I and G then they randomly combine in the ratios $I^2 : G^2 : 2IG$ representing ratios of IR : IGF-1R : Hybrids. We used the amount of detected IR-A mRNA as (I) and the amount of detected IGF-1R mRNA as (G) for our rough calculations. [b]In the case of mouse forebrain, we used a sum of IR-A and IR-B as (I). The summa of $I^2 + G^2 + 2IG$ was then considered as total amount of the receptors (100 %) and the homodimers and hybrid receptors are shown as % from all receptors. This calculation does not provide information about quantity of the receptors but just addresses their theoretical ratios in the specific cell line.

Table S4. Primers for qRT-PCR.

receptor	species	forward	reverse
IR-A	human	TTTTCGTCCCCAGGCCATC	GTCACATTCCCAACATCGCC
IR-B	human	CCCCAGAAAAACCTCTTCAGG	GTCACATTCCCAACATCGCC
IGF-1R	human	GGCACAATTACTGCTCCAAAGAC	CAAGGCCCTTTCTCCCCAC
Actin	human	AGCCATGTACGTTGCTATCCA	ACCGGAGTCCATCACGATG
IR-A	mouse	TCCTGAAGGAGCTGGAGGAGT	CTTTCGGGATGGCCTGG
IR-B	mouse	TCCTGAAGGAGCTGGAGGAGT	TTCGGGATGGCCTACTGTC
IGF-1R	mouse	GGCACAACTACTGCTCCAAAGAC	CTTTATCACCACCACACACTTCTG
Actin	mouse	AGCCATGTACGTAGCCATCCA	TCTCCGGAGTCCATCACAATG

Table S5. Predicted ratios of receptor forms in breast cancer cell lines. Predictions are based on receptor mRNA expression levels and the theory of random assembly. The mRNA levels were normalized to actin expression in each cell line and expressed as a percentage of actin (mean ± S.D., n = 4). The proportions of homodimers and hybrid receptors are estimated as percentages (%) of the total receptor population in each cell line.

Cell line	Human IR-A mRNA	Human IR-B mRNA	Human IGF-1R mRNA	IR-A (%)[a] homodimer	IGF-1R (%)[a] homodimer	IR-A/ IGF-1R (%)[a] hybrid
SH-SY5Y	0.12±0.03	0.01 % ± 0.01	0.82±0.15	1.7	76.0	22.3
	0.44±0.05[b]	-	2.45±0.44[b]	2.3[b]	71.9[b]	25.8[b]
MCF-7	0.046±0.005	0.02±0.004	4.96±1.29	0.02	97.5	2.5
MDA-MD-231	0.014±0.003	0.005±0.001	0.25±0.1	0.5	85.5	14

[a] The theory of random assembly (*29*) states that if the total concentrations of IR and IGF-1R subunits are denoted as *I* and *G*, respectively, they combine randomly in the ratios $I^2 : G^2 : 2IG$, representing the relative proportions of IR homodimers, IGF-1R homodimers, and hybrid receptors. For our approximate calculations, we used the amount of detected IR-A mRNA as *I* and the amount of detected IGF-1R mRNA as *G*. In some analyses, the sum of IR-A and IR-B mRNA levels was used as *I*. The sum $I^2 + G^2 + 2IG$ was considered as the total receptor pool (100%), and the proportions of homodimers and hybrid receptors were expressed as percentages of all receptors. This approach does not provide absolute receptor quantities but rather estimates their theoretical ratios within each specific cell line.

[b] Same as in Table S3.

Supplementary Table S6. Selected phosphosites identified in SH-SY5Y cells after stimulation with 1$_{Ins}$, IGF-1 and insulin. We show the protein name, its abbreviation, and accession number according to the UniProt database, together with the predicted phosphosite (Y/S/T) and the number of predicted phosphate groups within the identified peptide (M). We provide subtracted binary logarithms of intensities (fold change) between the analog and control untreated cells, as well as the negative log of the Student's t-test p-value (−log p; gray background, with numbers in bold indicating significant changes with FDR < 0.15, numbers in italics were significant only at FDR 0.15, and others were significant at FDR < 0.05). Phosphosites similarly regulated by all ligands are highlighted in italics, those upregulated by both **1**$_{Ins}$ and IGF-1 are shown in bold, and phosphosites upregulated by a single ligand are marked as underlined. An apostrophe (^{ins} or $^{IGF\text{-}1}$) indicates a significant difference between treatments at FDR < 0.15.

Abrev.	Protein name	UniProt accession phosphosite	Sequence surrounding the phosphosite	**1**$_{Ins}$ fold change	-log p	IGF-1 fold change	-log p	Insulin fold change	-log p
IGF-1R /IR	Insulin-like growth factor 1 receptor /Insulin receptor P06213	P08069_Y1165_M2	RDIYETDYYRKGGKG	**30.74** ^{ins}	**6.5658**	**20.40** ^{ins}	**6.3472**	1.05	0.0442
		P06213_Y1189_M2	RDIYETDYYRKGGKG	**32.36** ^{ins}	**7.0119**	**21.48** ^{ins}	**6.8675**	1.04	0.3416
		P08069_Y1161_M2	FGMTRDIYETDYYRK	**35.15** ^{ins}	**6.5111**	**23.33** ^{ins}	**6.2822**	1.61	0.5905
		P06213_Y1185_M2	FGMTRDIYETDYYRK	**26.00** ^{ins}	**6.1309**	**17.26** ^{ins}	**5.8536**	1.92	1.2048
IRS2	Insulin receptor substrate 2	Q9Y4H2_S309_M2	PRSKSQSSGSSATHP	28.72	**5.7440**	11.35	**2.5195**	7.66	1.9227
		Q9Y4H2_S306_M2	EFRPRSKSQSSGSSA	30.46	**6.2907**	11.23	**2.3348**	8.01	***2.3487***
		Q9Y4H2_T520_M2	FCSHRSNTPESIAET	*9.05*	**2.4192**	*6.01*	***1.5969***	*4.60*	1.4060
		Q9Y4H2_S523_M2	HRSNTPESIAETPPA	*6.26*	**2.4214**	*4.16*	***1.6496***	*4.41*	1.6541
		Q9Y4H2_T527_M3	TPESIAETPPARDGG	*3.61*	***2.9858***	*2.29*	0.9430	*1.77*	0.5826
		Q9Y4H2_S577_M1	GLRKRTYSLTTPARQ	*6.34*	**5.7852**	*5.83*	**5.5074**	*5.83*	***4.8444***
		Q9Y4H2_Y823_M1	CGGDSDQYVLMSSPV	*6.46*	**5.1338**	*6.22*	**4.9212**	*6.10*	***4.4060***
		Q9Y4H2_Y919_M2	PLPPEPKSPGEYINI	**31.34**	**6.3113**	**26.45**	**6.0681**	16.50	**4.4736**
		Q9Y4H2_S915_M2	PLPPEPKSPGEYINI	**30.19**	**7.4231**	**25.48**	**7.1404**	15.89	**5.1928**
		Q9Y4H2_Y653_M1	NLGADDGYMPMTPGA	*3.07*	**4.4429**	*3.44*	**5.3581**	*1.94*	1.4642
		Q9Y4H2_Y598_M1	SSASLDEYTLMRATF	*4.14*	**5.0506**	*2.89*	***3.9754***	*2.64*	2.4287
		Q9Y4H2_Y675_M1	GSCRSDDYMPMSPAS	*15.14*	**6.0284**	*16.67*	**6.0661**	*18.98*	**6.1468**
Shc1	SHC-transforming protein 1	P29353_S139_M1	EEWTRHGSFVNKPTR	*3.89*	**5.1558**	*3.44*	***4.4847***	*2.47*	3.0035
GAB2	GRB2-associated-binding protein 2	Q9UQC2_S623_M2	ALDFQPSSPSPHRKP	18.93 ^{ins}	**7.9850**	2.47	0.8921	2.62	1.0858
		Q9UQC2_Y614_M2	KSTGSVDYLALDFQP	15.00 ^{ins}	**5.4403**	2.18	0.7141	2.20	0.6934

		Q9UQC2_S264_M1	HNTEFRDSTYDLPRS	*5.01*	**5.1427**	*4.69*	**4.7240**	*4.06*	***4.0754***
		Q9UQC2_Y643_M1	TSDEKVDYVQVDKEK	*6.13*	**3.8400**	*3.42*	***2.5522***	*3.16*	1.9462
EFS	Embryonal Fyn-associated substrate	O43281_S298_M1	PHRPRLPSAESLSRR	*6.72*	**9.8577**	*7.50*	**9.7311**	*6.63*	**10.2501**
		O43281_S301_M2	PRLPSAESLSRRPLP	*14.17*	**7.1552**	*13.71*	**6.8803**	*11.31*	**5.6758**
		O43281_S298_M2	PHRPRLPSAESLSRR	*14.97*	**7.0613**	*14.49*	**6.8055**	*11.95*	**5.6227**
SH3R1	E3 ubiquitin-protein ligase SH3RF1	Q7Z6J0_S735_M2	TKRKPRVSPPASPTL	47.13 [ins]	**7.0336**	2.80	0.5563	3.09	0.5324
		Q7Z6J0_S739_M2	PRVSPPASPTLEVEL	49.30 [ins]	**8.5901**	6.30	***1.3434***	5.02	1.0653
Akt3	RAC-gamma S/T-protein kinase	Q9Y243_S476_M2	RPHFPQFSYSASGRE	**180.17**	**8.7845**	**157.16**	**8.8341**	97.58	**6.9685**
		Q9Y243_S472_M2	PQFSYSASGRE	**173.18**	**9.0314**	**151.08**	**9.1099**	94.46	**7.1494**
		Q9Y243_T305_M1	TDAATMKTFCGTPEY	**19.30**	**8.3383**	**18.10**	**8.2274**	7.42	***2.2077***
Erk1	Mitogen-activated protein kinase 3	P27361_T207_M2	FLTEYVATRWYRAPE	*6.12*	**5.7640**	*4.25*	**4.2613**	*3.39*	3.8576
		P27361_Y204_M1	HTGFLTEYVATRWYR	*3.47*	**2.6805**	*2.90*	**2.7543**	*2.01*	1.4519
Erk2	Mitogen-activated protein kinase 1	P28482_Y187_M1	HTGFLTEYVATRWYR	*2.68*	**7.2729**	*2.35*	***5.4783***	*2.11*	5.1810
TSC2	Tuberin	P49815_T1462_M1	GLRPRGYTISDSAPS	*6.99*	**4.5671**	*6.53*	**4.5122**	*7.20*	***4.4070***
FOXO3	Forkhead box protein O3	O43524_S43_M2	QRPELQASPAKPSGE	*9.37*	**4.2794**	*9.36*	**4.3494**	*9.23*	***3.7692***
		O43524_T32_M2	QSRPRSCTWPLQRPE	*9.62*	**7.2471**	*9.62*	**7.6323**	*9.48*	**6.6745**
KS6A3	Ribosomal protein S6 kinase alpha-3	P51812_T365_M3	DPEFTAKTPKDSPGI	**89.09** [ins]	**4.0545**	**39.12**	**3.1219**	5.78	0.7754
		P51812_S375_M3	DSPGIPPSANAHQLF	**63.88** [ins]	**3.8634**	**28.05**	**2.8900**	4.73	0.7039
		P51812_S369_M3	TAKTPKDSPGIPPSA	**71.90** [ins]	**4.0681**	**31.57**	**3.0743**	5.89	0.9068
KS6A1	Ribosomal protein S6 kinase alpha-1	Q15418_S369_M2	DSPGIPPSAGAHQLF	**19.52**	**3.4946**	**14.79**	**3.1996**	8.57	***2.1377***
		Q15418_S363_M2	TSRTPKDSPGIPPSA	**16.66**	**3.3239**	**12.59**	**3.0134**	7.35	1.9636
DAPK1	Death-associated protein kinase 1	P53355_S368_M1	QHLLGSLSNYDVNQP	4.85	***1.2165***	1.69	0.2116	2.41	0.4003
RS6	Small ribosomal subunit protein eS6	P62753_T241_M3	LSSLRASTSKSESSQ	11.99 [ins]	**5.9529**	2.17	0.5824	1.00	NaN
		P62753_S240_M3	RLSSLRASTSKSESS	14.53	**3.4377**	3.46	0.7133	4.74	1.2191
		P62753_S236_M3	AKRRRLSSLRASTSK	13.46	**3.3942**	3.45	0.7619	4.62	1.2785
		P62753_S235_M3	IAKRRRLSSLRASTS	10.50	**3.6378**	2.81	0.6918	3.84	1.3550

RL19	Large ribosomal subunit protein eL19	P84098_S13_M2<br>P84098_S12_M2	LQKRLASSVLRCGKK<br>RLQKRLASSVLRCGK	3.57 [IGF-1]<br>2.61 [IGF-1]	0.8087<br>0.7899	-6.97<br>-12.60	***1.2924***<br>**2.4051**	-1.26<br>-1.61	0.0694<br>0.1831
VIP2	Inositol hexakisphosphate and diphosphoinositol-pentakisphosphate kinase 2	O43314_S1016_M2	QITSSPVSPKSLAFT	7.25 [ins]	***1.4928***	1.98	0.2689	-2.02	0.2819
SDS3	Sin3 histone deacetylase corepressor complex component SDS3	Q9H7L9_S237_M2	KRPASPSSPEHLPAT	32.79	**1.2594**	1.09	0.0133	7.50	0.4322
MDC1	Mediator of DNA damage checkpoint protein 1	Q14676_S307_M3	DTDVDDDSRPPGRPA	28.58	**2.7757**	1.64	0.2111	2.83	0.3961
PKCD3	Serine/threonine-protein kinase D3	O94806_S44_M2	SARLSNGSFSAPSLT	1.41	0.1608	4.39	***1.5888***	1.57	0.2163
PDE4B	3',5'-cyclic-AMP phosphodiesterase 4B	Q07343_S319_M1	KKLMHSSSLNNTSIS	5.12	***1.3047***	47.02	**7.9582**	11.39	**1.8121**
DDX21	Nucleolar RNA helicase 2	Q9NR30_S173_M3<br>Q9NR30_S171_M3<br>Q9NR30_S168_M3	AASEESNSEIEQEIP<br>SEAASEESNSEIEQE<br>CNPSEAASEESNSEI	-1.13<br>-1.08<br>-1.40	0.0204<br>0.0140<br>0.0624	16.89<br>13.39<br>13.49	***1.1009***<br>***1.0852***<br>***1.0918***	1.98<br>1.53<br>1.91	0.1240<br>0.0766<br>0.1276
CCD86	Coiled-coil domain-containing protein 86	Q9H6F5_S113_M2<br>Q9H6F5_S69_M3<br>Q9H6F5_S58_M3	QPEYSPESPRCQPKP<br>PPKTSPGSPRLQQGA<br>VQRAGLGSPERPPKT	7.48<br>8.96<br>6.38	***1.2708***<br>**2.0956**<br>**1.8191**	2.05<br>28.08<br>21.12	0.2451<br>**6.4051**<br>**7.1034**	6.23<br>3.18<br>3.44	0.9756<br>0.9555<br>1.4113

SRRM1	Serine/arginine repetitive matrix protein 1	Q8IYB3_S562_M2	TRRRRSPSPAPPPRR	-1.65 [ins]	1.1931	-1.97	2.0868	-8.23	**6.1205**
		Q8IYB3_S393_M3	RRLSPSASPPRRRHR	**4.32**	***1.2553***	**3.68**	1.1555	-1.01	0.0036
		Q8IYB3_S775_M2	PSPVQSQSPSTNWSP	9.20	0.7090	19.05	***1.0541***	47.79	***1.7578***
		Q8IYB3_T574_M2	PRRRRTPTPPPRRRT	-1.65	0.7367	-2.47	1.3109	-9.86	***2.1595***
EHB1	EH domain-binding protein 1	Q8NDI1_S428_M3	AGKDLSTSPKPSPIP	-21.02	**1.4857**	-13.14	0.9850	-157.31	**2.9895**
DCX	Neuronal migration protein doublecortin	O43602_S332_M3	STPKSKQSPISTPTS	-1.92 [ins]	2.9030	-1.95[ins]	3.1240	-19.27	**3.3103**
		O43602_S339_M3	SPISTPTSPGSLRKH	-1.92 [ins]	2.9030	-1.95	3.1240	-12.56	***3.0251***
		O43602_S335_M3	KSKQSPISTPTSPGS	-1.92 [ins]	2.9030	-1.95	3.1240	-10.65	***2.9407***

Auxiliary Supplementary data file: Raw data (Excel file)
This Excel file contains the raw quantitative data used for the following analyses:

Binding to IGF-1R
Binding to IR-A
Binding to IR-B
Binding to IGF-2R D11
Binding to IGFBP-3
In Cell Western
Western Blots - transfected fibroblasts
qRT-PCR
Western Blots - rat postnatal cultures
Western Blots - SH-SY5Y
Western Blots - U87MG
Inhibition with S661 - breast cells
MTT test - rat postnatal culture
MTT test - SH-SY5Y
MTT test – SH-SY5Y co-stimulation
ITT test
Glucose euglycemic clamp

Individual worksheets correspond to individual experiments and are labeled accordingly.

Uncropped Blots

The images on the following pages show uncropped blots for Figures 2, 3, and S9.

Fig. 2

Rat neonatal neural cells

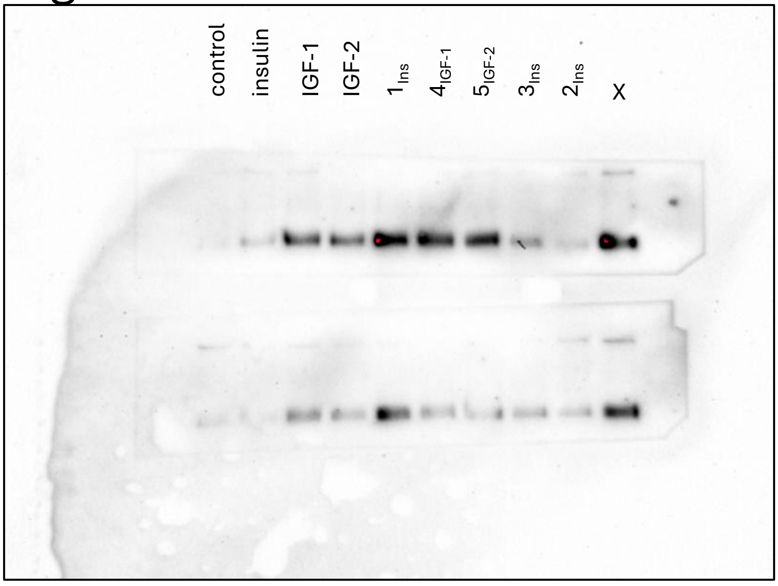

pY1150/1151 IR or pY1135/1136 IGF-1R, MW 100 kDa

gel top

cut 75 kDa

Duplicate - Supplementary material (Source data for Fig. 2, 3 and S9)

Fig. 3 D

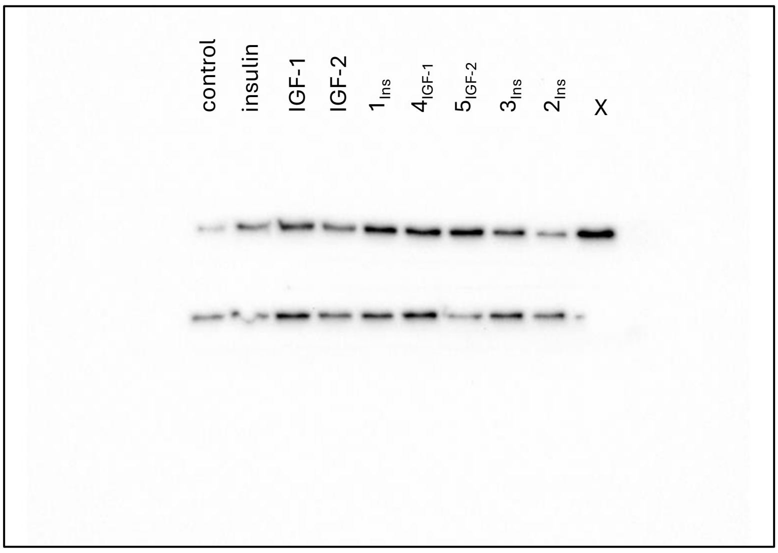

pAkt (T308) Mw 60 kDa

cut 75 kDa

cut 50 kDa

Duplicate - Supplementary material (Source data for Fig. 2, 3 and S9)

Fig. 3 H

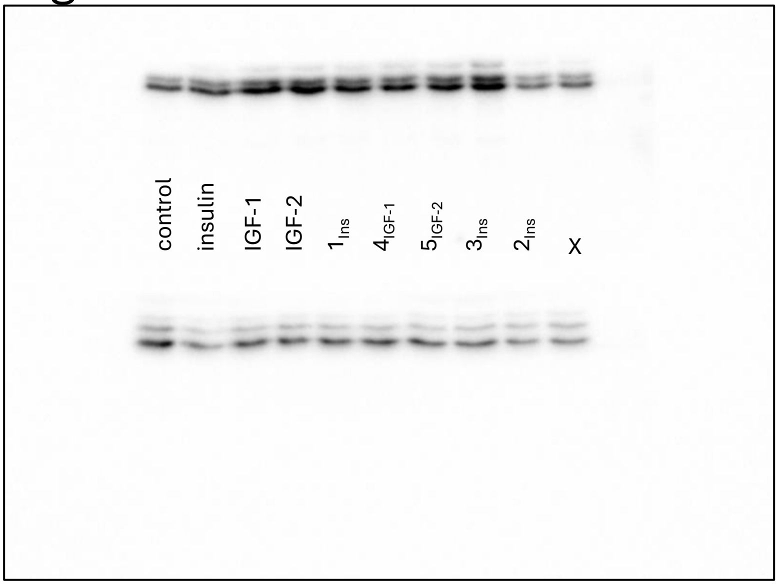

pErk1/2 (T202/Y204) Mw 42, 44 kDa

not included

cut 50 kDa

cut 25 kDa

Fig. 2, Fig. 3 D, H

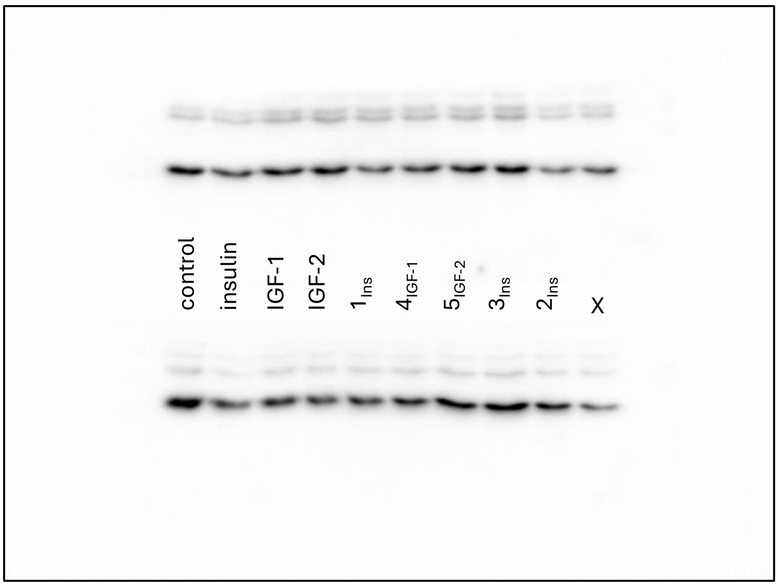

Restained for GAPDH Mw 38 kDa

not included

cut 50 kDa

cut 25 kDa

Transfected mouse fibroblasts – IGF-1R

Fig.: Supplementary material
(Source data for Fig. 2, 3 and S9)

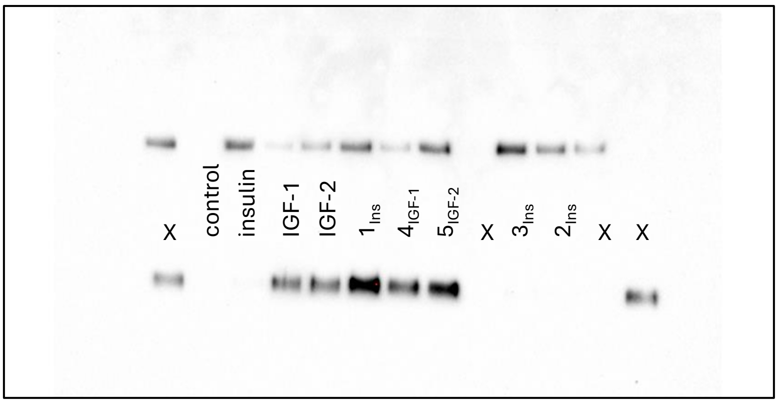

pY1150/1151 IR or pY1135/1136 IGF-1R, MW 100 kDa

← not included

gel top

cut 75 kDa

Fig. 3 A

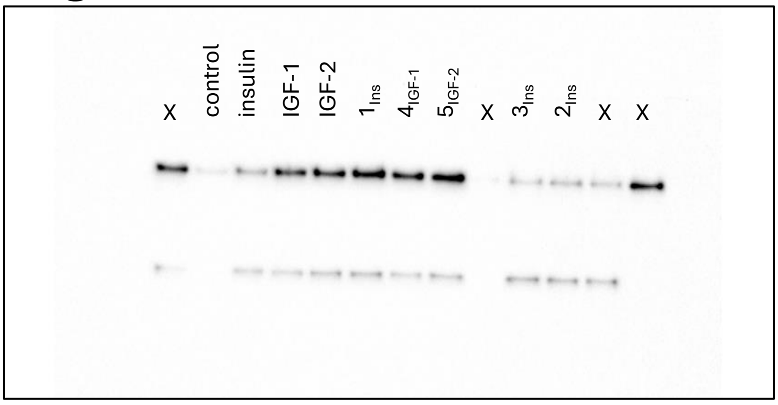

pAkt (T308) Mw 60 kDa

cut 75 kDa

cut 50 kDa

← not included

Fig. 3 E

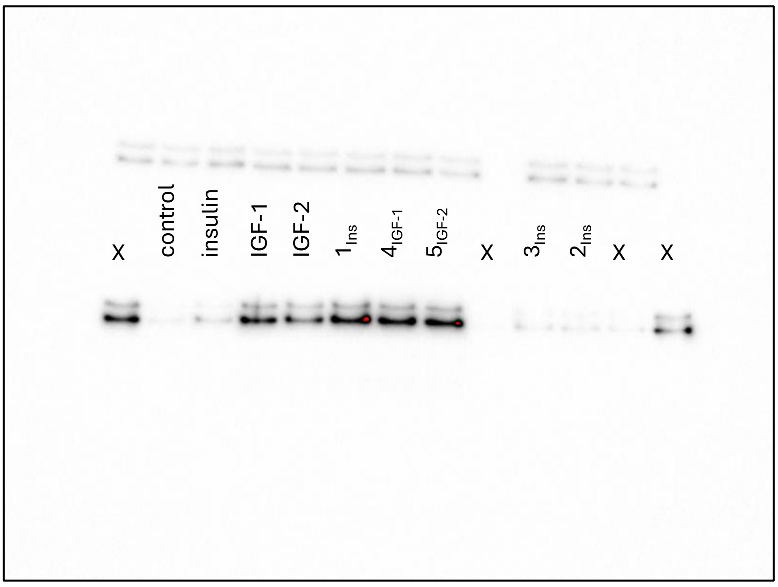

pErk1/2 (T202/Y204) Mw 42, 44 kDa

← not included

cut 50 kDa

cut 25 kDa

Fig. 3 A, E

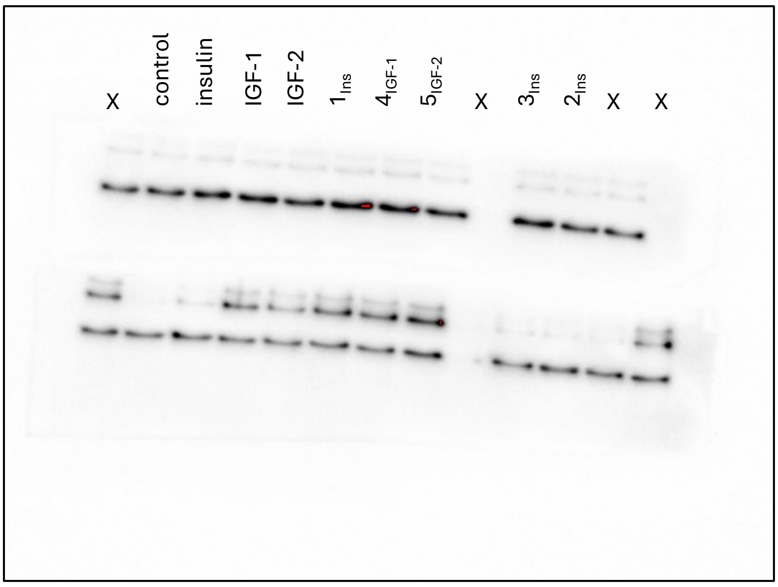

Restained for GAPDH Mw 38 kDa

← not included

cut 50 kDa

cut 25 kDa

SH-SY5Y

Fig. 3 B

pAkt (T308) Mw 60 kDa

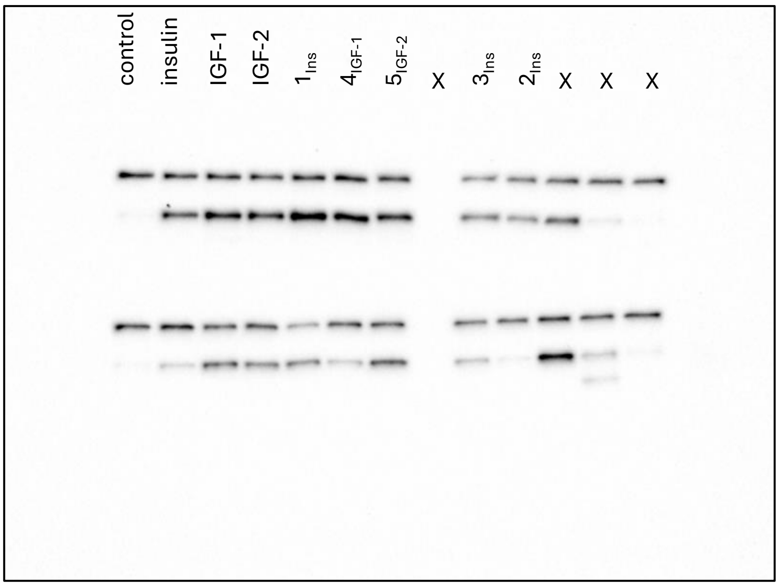

Gel top

Non-specific MW >75 kDa

cut 50 kDa

Duplicate - Supplementary material (Source data for Fig. 2, 3 and S9)

Fig. 3 F

pErk1/2 (T202/Y204) Mw 42, 44 kDa

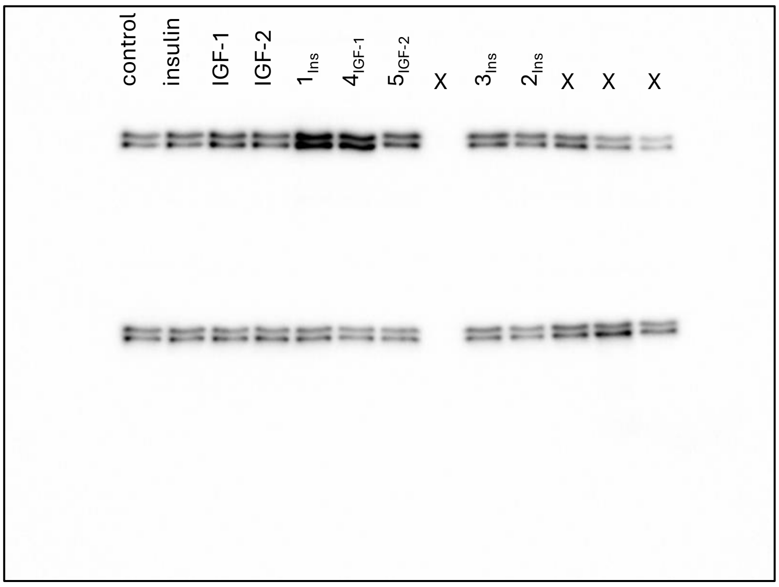

cut 50 kDa

cut 25 kDa

Duplicate - Supplementary material (Source data for Fig. 2, 3 and S9)

Fig. 3 B, F

Restained for GAPDH Mw 38 kDa

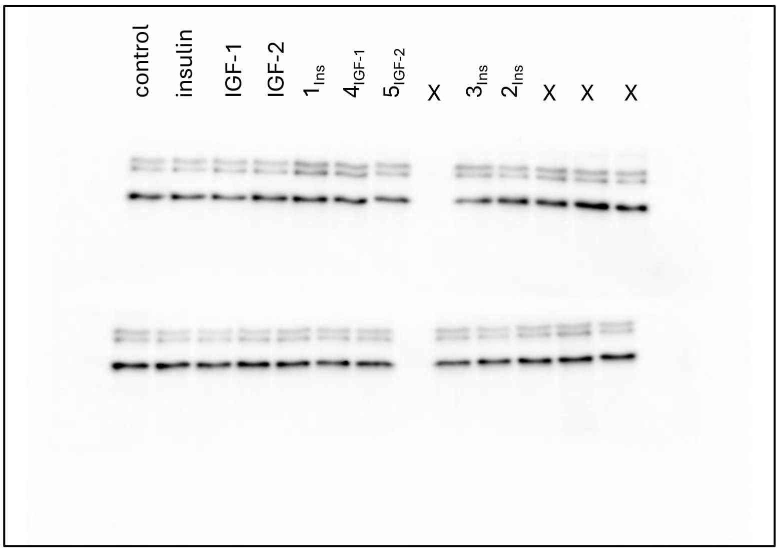

cut 50 kDa

cut 25 kDa

Duplicate - Supplementary material (Source data for Fig. 2, 3 and S9)

U87MG

Fig.: Supplementary material
(Source data for Fig. 2, 3 and S9)

pY1150/1151 IR or pY1135/1136 IGF-1R, MW 100 kDa

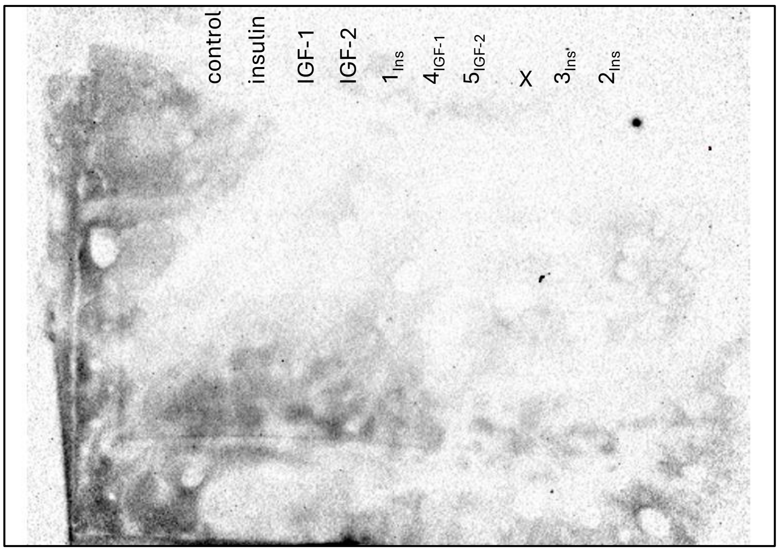

Gel top

cut 75 kDa

Duplicate - Supplementary material (Source data for Fig. 2, 3 and S9)

Fig. 3 C

pAkt (T308) Mw 60 kDa

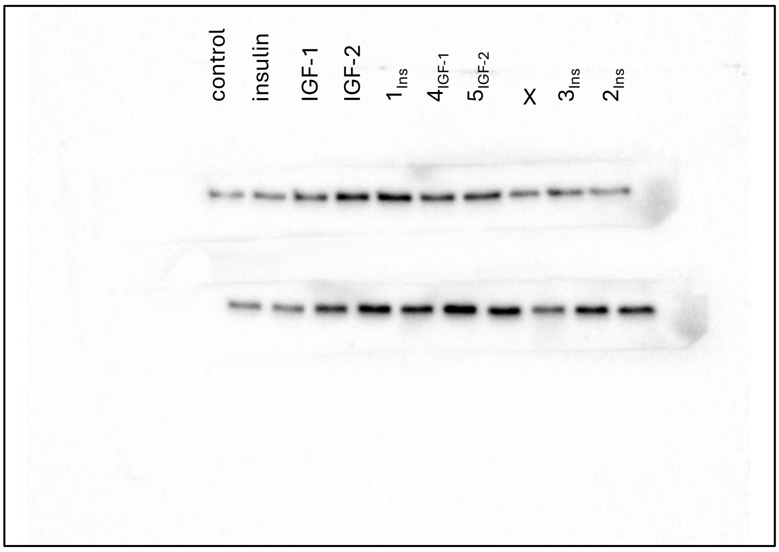

cut 75 kDa

cut 50 kDa

Duplicate - Supplementary material (Source data for Fig. 2, 3 and S9)

Fig. 3 G

pErk1/2 (T202/Y204) Mw 42, 44 kDa

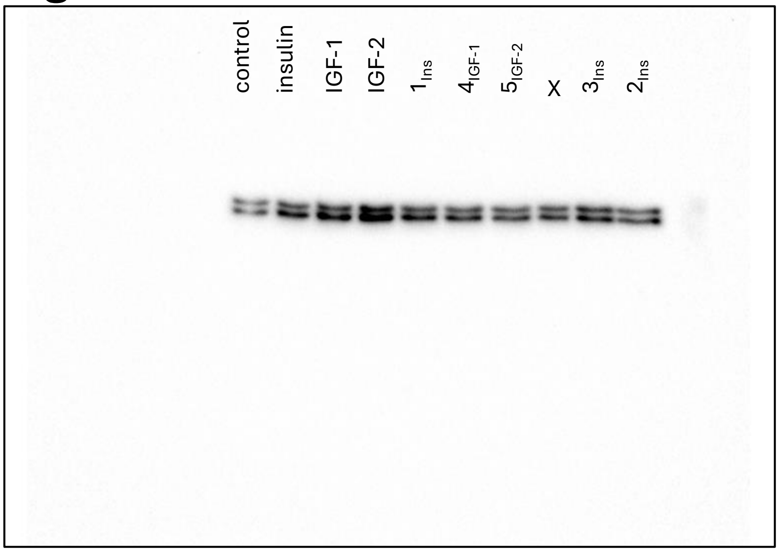

cut 50 kDa

cut 25 kDa

Fig. 3 C, G

Restained for GAPDH Mw 38 kDa

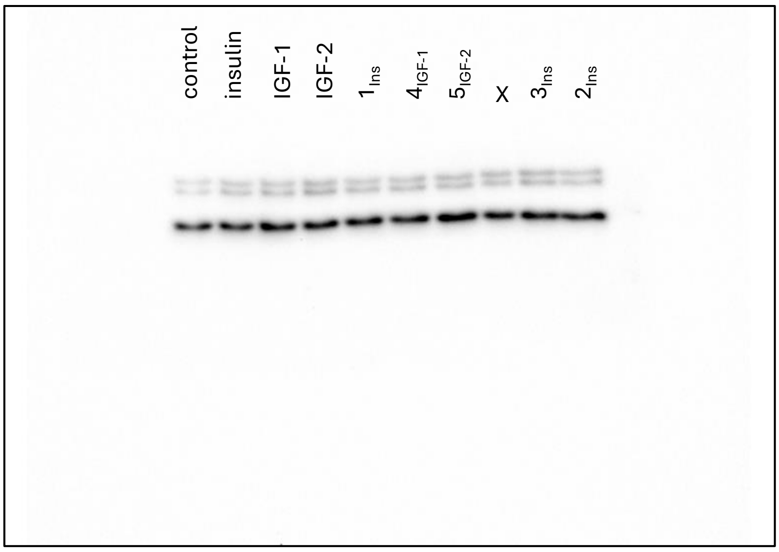

cut 50 kDa

cut 25 kDa

REFERENCES

1. M. F. White, C. R. Kahn, Insulin action at a molecular level - 100 years of progress. *Mol. Metab.* **52**, 101304 (2021).

2. D. LeRoith, J. M. P. Holly, B. E. Forbes, Insulin-like growth factors: Ligands, binding proteins, and receptors. *Mol. Metab.* **52**, 101245 (2021).

3. B. S. Miller, A. D. Rogol, R. G. Rosenfeld, The history of the insulin-like growth factor system. *Horm. Res. Paediatr.* **95**, 619–630 (2022).

4. M. A. Lemmon, J. Schlessinger, Cell signaling by receptor tyrosine kinases. *Cell* **141**, 1117–1134 (2010).

5. E. Choi, C. Duan, X. C. Bai, Regulation and function of insulin and insulin-like growth factor receptor signalling. *Nat. Rev. Mol. Cell Biol.* **26**, 558–580 (2025).

6. A. Belfiore, F. Frasca, G. Pandini, L. Sciacca, R. Vigneri, Insulin receptor isoforms and insulin receptor/insulin-like growth factor receptor hybrids in physiology and disease. *Endocr. Rev.* **30**, 586–623 (2009).

7. E. M. Bailyes, B. T. Nave, M. A. Soos, S. R. Orr, A. C. Hayward, K. Siddle, Insulin receptor/IGF-I receptor hybrids are widely distributed in mammalian tissues: Quantification of individual receptor species by selective immunoprecipitation and immunoblotting. *Biochem. J.* **327**, 209–215 (1997).

8. R. Slaaby, Specific insulin/IGF1 hybrid receptor activation assay reveals IGF1 as a more potent ligand than insulin. *Sci. Rep.* **5**, 7911 (2015).

9. N. Dahms, T. Braulke, A. Varki, "P-type lectins" in *Essentials of Glycobiology*, (Cold Spring Harbor, 2022), pp. 443–454.

10. H. Nagao, W. Cai, N. J. Wewer Albrechtsen, M. Steger, T. M. Batista, H. Pan, J. M. Dreyfuss, M. Mann, C. R. Kahn, Distinct signaling by insulin and IGF-1 receptors and their extra- and intracellular domains. *Proc. Natl. Acad. Sci. U.S.A.* **118**, e2019474118 (2021).

11. K. Siddle, Molecular basis of signaling specificity of insulin and IGF receptors: Neglected corners and recent advances. *Front. Endocrinol.* **3**, 34 (2012).

12. A. L. Fowden, The role of insulin in fetal growth. *Early Hum. Dev.* **29**, 177–181 (1992).

13. A. M. Fernandez, I. Torres-Aleman, The many faces of insulin-like peptide signalling in the brain. *Nat. Rev. Neurosci.* **13**, 225–239 (2012).

14. M. Heni, S. Kullmann, H. Preissl, A. Fritsche, H. U. Haring, Impaired insulin action in the human brain: Causes and metabolic consequences. *Nat. Rev. Endocrinol.* **11**, 701–711 (2015).

15. C. M. Alberini, IGF2 in memory, neurodevelopmental disorders, and neurodegenerative diseases. *Trends Neurosci.* **46**, 488–502 (2023).

16. S. K. Kullmann, A. Kleinridders, D. M. Small, A. Fritsche, H. U. Häring, H. Preissl, M. Heni, Central nervous pathways of insulin action in the control of metabolism and food intake. *Lancet Diabetes Endocrinol.* **8**, 524–534 (2020).

17. C. G. Ardanaz, A. de la Cruz, P. S. Minhas, N. Hernandez-Martin, M. A. Pozo, M. P. Valdecantos, A. M. Valverde, P. Villa-Valverde, M. Elizalde-Horcada, E. Puerta, M. J. Ramirez, J. E. Ortega, A. Urbiola, C. Ederra, M. Ariz, C. Ortiz-de-Solorzano, J. Fernandez-Irigoyen, E. Santamaria, G. Karsenty, J. C. Bruning, M. Solas, Astrocytic GLUT1 reduction paradoxically improves central and peripheral glucose homeostasis. *Sci. Adv.* **10**, eadp1115 (2024).

18. X. Tu, A. Jain, P. Parra Bueno, H. Decker, X. Liu, R. Yasuda, Local autocrine plasticity signaling in single dendritic spines by insulin-like growth factors. *Sci. Adv.* **9**, eadg0666 (2023).

19. M. Chrudinova, L. Zakova, A. Marek, O. Socha, M. Budesinsky, M. Hubalek, J. Picha, K. Machackova, J. Jiracek, I. Selicharova, A versatile insulin analog with high potency for both insulin and insulin-like growth factor 1 receptors: Structural implications for receptor binding. *J. Biol. Chem.* **293**, 16818–16829 (2018).

20. T. Panikova, K. Mitrova, T. Halamova, K. Mrzilkova, J. Picha, M. Chrudinova, A. Kurochka, I. Selicharova, L. Zakova, J. Jiracek, Insulin analogues with altered insulin receptor isoform binding specificities and enhanced aggregation stabilities. *J. Med. Chem.* **64**, 14848–14859 (2021).

21. J. Vikova, M. Collinsova, E. Kletvikova, M. Budesinsky, V. Kaplan, L. Zakova, V. Veverka, R. Hexnerova, R. J. T. Avino, J. Strakova, I. Selicharova, V. Vanek, D. W. Wright, C. J. Watson, J. P. Turkenburg, A. M. Brzozowski, J. Jiracek, Rational steering of insulin binding specificity by intra-chain chemical crosslinking. *Sci. Rep.* **6**, 19431 (2016).

22. K. Machackova, M. Chrudinova, J. Radosavljevic, P. Potalitsyn, K. Krizkova, M. Fabry, I. Selicharova, M. Collinsova, A. M. Brzozowski, L. Zakova, J. Jiracek, Converting insulin-like growth factors 1 and 2 into high-affinity ligands for insulin receptor isoform A by the introduction of an evolutionarily divergent mutation. *Biochemistry* **57**, 2373–2382 (2018).

23. P. Potalitsyn, I. Selicharova, K. Srsen, J. Radosavljevic, A. Marek, K. Novakova, J. Jiracek, L. Zakova, A radioligand binding assay for the insulin-like growth factor 2 receptor. *PLOS ONE* **15**, e0238393 (2020).

24. P. Potalitsyn, L. Mrazkova, I. Selicharova, M. Tencerova, M. Ferencakova, M. Chrudinova, T. Turnovska, A. M. Brzozowski, A. Marek, J. Kaminsky, J. Jiracek, L. Zakova, Non-glycosylated IGF2 prohormones are more mitogenic than native IGF2. *Commun. Biol.* **6**, 863 (2023).

25. E. Uchikawa, E. Choi, G. J. Shang, H. T. Yu, X. C. Bai, Activation mechanism of the insulin receptor revealed by cryo-EM structure of the fully liganded receptor-ligand complex. *eLife* **8**, 48630 (2019).

26. J. Li, E. Choi, H. Yu, X. C. Bai, Structural basis of the activation of type 1 insulin-like growth factor receptor. *Nat. Commun.* **10**, 4567 (2019).

27. Y. Xu, N. S. Kirk, H. Venugopal, M. B. Margetts, T. I. Croll, J. J. Sandow, A. I. Webb, C. A. Delaine, B. E. Forbes, M. C. Lawrence, How IGF-II binds to the human type 1 insulin-like growth factor receptor. *Structure* **28**, 786–798.e6 (2020).

28. L. Zakova, E. Kletvikova, V. Veverka, M. Lepsik, C. J. Watson, J. P. Turkenburg, J. Jiracek, A. M. Brzozowski, Structural integrity of the B24 Site in human insulin is important for hormone functionality. *J. Biol. Chem.* **288**, 10230–10240 (2013).

29. G. Pandini, R. Vigneri, A. Costantino, F. Frasca, A. Ippolito, Y. Fujita-Yamaguchi, K. Siddle, I. D. Goldfine, A. Belfiore, Insulin and insulin-like growth factor-I (IGF-I) receptor overexpression in breast cancers leads to insulin/IGF-I hybrid receptor overexpression: Evidence for a second mechanism of IGF-I signaling. *Clin. Cancer Res.* **5**, 1935–1944 (1999).

30. J. Voldrich, M. Matousova, M. Smidkova, B. Slavikova, H. Chodounska, E. Kudova, H. Mertlikova-Kaiserova, Identification of N-methyl-D-aspartate receptor antagonists using the rat postnatal mixed cortical and hippocampal neurons. *Eur. J. Pharmacol.* **927**, 175056 (2022).

31. L. Schaffer, C. L. Brand, B. F. Hansen, U. Ribel, A. C. Shaw, R. Slaaby, J. Sturis, A novel high-affinity peptide antagonist to the insulin receptor. *Biochem. Biophys. Res. Commun.* **376**, 380–383 (2008).

32. M. Polak, I. Selicharova, M. Pinkas, M. S. Garre Hernández, B. Fabre, M. Lubos, L. Zakova, M. Grzybek, I. B. Schäfer, Ü. Coskun, J. Jiracek, J. Novacek, Antagonistic insulin mimetics lock the insulin receptor in an alternative apo-state. bioRXiv 667158 [Preprint] (2025); https://doi.org/10.1101/2025.08.05.667158.

33. A. Konig, T. F. Outeiro, Diabetes and Parkinson's disease: Understanding shared molecular mechanisms. *J. Parkinsons Dis.* **14**, 917–924 (2024).

34. S. H. Park, H. I. Choi, J. Ahn, Y. J. Jang, T. Y. Ha, H. D. Seo, Y. S. Kim, D. H. Lee, C. H. Jung, Autophagy functions to prevent methylglyoxal-induced apoptosis in HK-2 cells. *Oxid. Med. Cell. Longev.* **2020**, 8340695 (2020).

35. S. J. Geffken, S. Moon, C. O. Smith, S. Tang, H. H. Lee, K. Lewis, C. W. Wong, Y. Huang, Q. Huang, Y. T. Zhao, W. Cai, Insulin and IGF-1 elicit robust transcriptional regulation to modulate autophagy in astrocytes. *Mol. Metab.* **66**, 101647 (2022).

36. L. Lopez-Suarez, S. A. Awabdh, X. Coumoul, C. Chauvet, The SH-SY5Y human neuroblastoma cell line, a relevant in vitro cell model for investigating neurotoxicology in human: Focus on organic pollutants. *Neurotoxicology* **92**, 131–155 (2022).

37. P. Kurtzhals, L. Schaffer, A. Sorensen, C. Kristensen, I. Jonassen, C. Schmid, T. Trub, Correlations of receptor binding and metabolic and mitogenic potencies of insulin analogs designed for clinical use. *Diabetes* **49**, 999–1005 (2000).

38. H. Hvid, M. J. Blouin, E. Birman, J. Damgaard, F. Poulsen, J. J. Fels, C. Fledelius, B. F. Hansen, M. Pollak, Treatment with insulin analog X10 and IGF-1 increases growth of colon cancer allografts. *PLOS ONE* **8**, e79710 (2013).

39. N. Tennagels, S. Welte, M. Hofmann, P. Brenk, R. Schmidt, U. Werner, Differences in metabolic and mitogenic signalling of insulin glargine and AspB10 human insulin in rats [corrected]. *Diabetologia* **56**, 1826–1834 (2013).

40. N. C. Kaarsholm, K. Norris, R. J. Jorgensen, J. Mikkelsen, S. Ludvigsen, O. H. Olsen, A. R. Sorensen, S. Havelund, Engineering stability of the insulin monomer fold with application to structure-activity relationships. *Biochemistry* **32**, 10773–10778 (1993).

41. J. Jiracek, I. Selicharova, L. Zakova, "Mutations at hypothetical binding site 2 in insulin and insulin-like growth factors 1 and 2" in *Vitamins and Hormones* (Elsevier, 2023), pp. 187–230.

42. J. Jiracek, L. Zakova, Structural perspectives of insulin receptor isoform-selective insulin analogs. *Front. Endocrinol.* **8**, 167 (2017).

43. W. An, C. Hall, J. Li, A. Hung, J. Wu, J. Park, L. Wang, X. C. Bai, E. Choi, Activation of the insulin receptor by insulin-like growth factor 2. *Nat. Commun.* **15**, 2609 (2024).

44. A. Belfiore, R. Malaguarnera, V. Vella, M. C. Lawrence, L. Sciacca, F. Frasca, A. Morrione, R. Vigneri, Insulin receptor isoforms in physiology and disease: An updated view. *Endocr. Rev.* **38**, 379–431 (2017).

45. Y. Xu, M. B. Margetts, H. Venugopal, J. G. Menting, N. S. Kirk, T. I. Croll, C. Delaine, B. E. Forbes, M. C. Lawrence, How insulin-like growth factor I binds to a hybrid insulin receptor type 1 insulin-like growth factor receptor. *Structure* **30**, 1098–1108.e6 (2022).

46. C. Benedict, M. Hallschmid, A. Hatke, B. Schultes, H. L. Fehm, J. Born, W. Kern, Intranasal insulin improves memory in humans. *Psychoneuroendocrinology* **29**, 1326–1334 (2004).

47. Y. Y. Grinberg, L. A. Zitzow, R. P. Kraig, Intranasally administered IGF-1 inhibits spreading depression in vivo. *Brain Res.* **1677**, 47–57 (2017).

48. D. Kellar, S. Craft, Brain insulin resistance in Alzheimer's disease and related disorders: Mechanisms and therapeutic approaches. *Lancet Neurol.* **19**, 758–766 (2020).

49. M. Chrudinova, F. Moreau, H. L. Noh, T. Panikova, L. Zakova, R. H. Friedline, F. A. Valenzuela, J. K. Kim, J. Jiracek, C. R. Kahn, E. Altindis, Characterization of viral insulins reveals white adipose tissue-specific effects in mice. *Mol. Metab.* **44**, 101121 (2020).

50. T. Kenakin, Biased receptor signaling in drug discovery. *Pharmacol. Rev.* **71**, 267–315 (2019).

51. J. S. Smith, R. J. Lefkowitz, S. Rajagopal, Biased signalling: From simple switches to allosteric microprocessors. *Nat. Rev. Drug Discov.* **17**, 243–260 (2018).

52. S. Versteyhe, B. Klaproth, R. Borup, J. Palsgaard, M. Jensen, S. G. Gray, P. De Meyts, IGF-I, IGF-II, and insulin stimulate different gene expression responses through binding to the IGF-I receptor. *Front. Endocrinol.* **4**, 98 (2013).

53. B. F. Hansen, P. Kurtzhals, A. B. Jensen, A. Dejgaard, D. Russell-Jones, Insulin X10 revisited: A super-mitogenic insulin analogue. *Diabetologia* **54**, 2226–2231 (2011).

54. A. M. Jastreboff, R. F. Kushner, New frontiers in obesity treatment: GLP-1 and nascent nutrient-stimulated hormone-based therapeutics. *Annu. Rev. Med.* **74**, 125–139 (2023).

55. C. A. Gilroy, M. E. Capozzi, A. K. Varanko, J. Tong, D. A. D'Alessio, J. E. Campbell, A. Chilkoti, Sustained release of a GLP-1 and FGF21 dual agonist from an injectable depot protects mice from obesity and hyperglycemia. *Sci. Adv.* **6**, eaaz9890 (2020).

56. J. Petersen, M. Q. Ludwig, V. Juozaityte, P. Ranea-Robles, C. Svendsen, E. Hwang, A. W. Kristensen, N. Fadahunsi, J. Lund, A. W. Breum, C. V. Mathiesen, L. Sachs, R. Moreno-Justicia, R. Rohlfs, J. C. Ford, J. D. Douros, B. Finan, B. Portillo, K. Grose, J. E. Petersen, M. Trauelsen, A. Feuchtinger, R. D. DiMarchi, T. W. Schwartz, A. S. Deshmukh, M. B. Thomsen, K. A. Kohlmeier, K. W. Williams, T. H. Pers, B. Frolund, K. Stromgaard, A. B. Klein, C. Clemmensen, GLP-1-directed NMDA receptor antagonism for obesity treatment. *Nature* **629**, 1133–1141 (2024).

57. S. Craft, R. Raman, T. W. Chow, M. S. Rafii, C. K. Sun, R. A. Rissman, M. C. Donohue, J. B. Brewer, C. Jenkins, K. Harless, D. Gessert, P. S. Aisen, Safety, efficacy, and feasibility of intranasal insulin for the treatment of mild cognitive impairment and Alzheimer disease dementia: A randomized clinical trial. *JAMA Neurol.* **77**, 1099–1109 (2020).

58. X. F. Liu, J. R. Fawcett, R. G. Thorne, T. A. DeFor, W. H. Frey II, Intranasal administration of insulin-like growth factor-I bypasses the blood-brain barrier and protects against focal cerebral ischemic damage. *J. Neurol. Sci.* **187**, 91–97 (2001).

59. G. Farias Quipildor, K. Mao, P. J. Beltran, N. Barzilai, D. M. Huffman, Modulation of glucose production by central insulin requires IGF-1 receptors in AgRP neurons. *Diabetes* **70**, 2237–2249 (2021).

60. M. G. Engel, J. Smith, K. Mao, G. F. Quipildor, M. H. Cui, M. Gulinello, C. A. Branch, S. E. Gandy, D. M. Huffman, Evidence for preserved insulin responsiveness in the aging rat brain. *Geroscience* **44**, 2491–2508 (2022).

61. S. S. Ahmad, H. J. Chun, K. Ahmad, S. Shaikh, J. H. Lim, S. Ali, S. S. Han, S. J. Hur, J. H. Sohn, E. J. Lee, I. Choi, The roles of growth factors and hormones in the regulation of muscle satellite cells for cultured meat production. *J. Anim. Sci. Technol.* **65**, 16–31 (2023).

62. C. Sell, G. Dumenil, C. Deveaud, M. Miura, D. Coppola, T. Deangelis, R. Rubin, A. Efstratiadis, R. Baserga, Effect of a null mutation of the insulin-like growth-factor-I receptor gene on growth and transformation of mouse embryo fibroblasts. *Mol. Cell. Biol.* **14**, 3604–3612 (1994).

63. F. Frasca, G. Pandini, P. Scalia, L. Sciacca, R. Mineo, A. Costantino, I. D. Goldfine, A. Belfiore, R. Vigneri, Insulin receptor isoform A, a newly recognized, high-affinity insulin-like growth factor II receptor in fetal and cancer cells. *Mol. Cell. Biol.* **19**, 3278–3288 (1999).

64. A. Morcavallo, M. Genua, A. Palummo, E. Kletvikova, J. Jiracek, A. M. Brzozowski, R. V. Iozzo, A. Belfiore, A. Morrione, Insulin and insulin-like growth factor II differentially regulate endocytic sorting and stability of insulin receptor isoform A. *J. Biol. Chem.* **287**, 11422–11436 (2012).

65. K. Krizkova, M. Chrudinova, A. Povalova, I. Selicharova, M. Collinsova, V. Vanek, A. M. Brzozowski, J. Jiracek, L. Zakova, Insulin-insulin-like growth factors hybrids as molecular probes of hormone:receptor binding specificity. *Biochemistry* **55**, 2903–2913 (2016).

66. P. Dzianova, S. Asai, M. Chrudinova, L. Kosinova, P. Potalitsyn, P. Sacha, R. Hadravova, I. Selicharova, J. Kriz, J. P. Turkenburg, A. M. Brzozowski, J. Jiracek, L. Zakova, The efficiency of insulin production and its content in insulin-expressing model β-cells correlate with their Zn^{2+} levels. *Open Biol.* **10**, 200137 (2020).

67. A. Zmeskalova, A. Popelova, A. Exnerova, B. Zelezna, J. Kunes, L. Maletinska, Cellular signaling and anti-apoptotic effects of prolactin-releasing peptide and its analog on SH-SY5Y cells. *Int. J. Mol. Sci.* **21**, 6343 (2020).

68. S. Asai, L. Zakova, I. Selicharova, A. Marek, J. Jiracek, A radioligand receptor binding assay for measuring of insulin secreted by MIN6 cells after stimulation with glucose, arginine, ornithine, dopamine, and serotonin. *Anal. Bioanal. Chem.* **413**, 4531–4543 (2021).

69. L. Kosinova, V. Veverka, P. Novotna, M. Collinsova, M. Urbanova, N. R. Moody, J. P. Turkenburg, J. Jiracek, A. M. Brzozowski, L. Zakova, Insight into the structural and

biological relevance of the T/R transition of the N-terminus of the B-chain in human insulin. *Biochemistry* **53**, 3392–3402 (2014).

70. A. Kertisova, L. Zakova, K. Machackova, A. Marek, P. Sacha, P. Pompach, J. Jiracek, I. Selicharova, Insulin receptor Arg717 and IGF-1 receptor Arg704 play a key role in ligand binding and in receptor activation. *Open Biol.* **13**, 230142 (2023).

71. K. Machackova, M. Collinsova, M. Chrudinova, I. Selicharova, J. Picha, M. Budesinsky, V. Vanek, L. Zakova, A. M. Brzozowski, J. Jiracek, Insulin-like growth factor 1 analogs clicked in the C domain: Chemical synthesis and biological activities. *J. Med. Chem.* **60**, 10105–10117 (2017).

72. M. Lubos, J. Pícha, I. Selicharová, J. Zák, M. Budesínsky, K. Mitrová, L. Záková, J. Jirácek, Modulation of the antagonistic properties of an insulin mimetic peptide by disulfide bridge modifications. *J. Pept. Sci.* **29**, e3478 (2023).

73. F. Weis, J. G. Menting, M. B. Margetts, S. J. Chan, Y. Xu, N. Tennagels, P. Wohlfart, T. Langer, C. W. Muller, M. K. Dreyer, M. C. Lawrence, The signalling conformation of the insulin receptor ectodomain. *Nat. Commun.* **9**, 4420 (2018).

74. D. M. Huffman, G. Farias Quipildor, K. Mao, X. Zhang, J. Wan, P. Apontes, P. Cohen, N. Barzilai, Central insulin-like growth factor-1 (IGF-1) restores whole-body insulin action in a model of age-related insulin resistance and IGF-1 decline. *Aging Cell* **15**, 181–186 (2016).

75. R. H. Muzumdar, D. M. Huffman, G. Atzmon, C. Buettner, L. J. Cobb, S. Fishman, T. Budagov, L. Cui, F. H. Einstein, A. Poduval, D. Hwang, N. Barzilai, P. Cohen, Humanin: A novel central regulator of peripheral insulin action. *PLOS ONE* **4**, e6334 (2009).

76. S. J. Humphrey, O. Karayel, D. E. James, M. Mann, High-throughput and high-sensitivity phosphoproteomics with the EasyPhos platform. *Nat. Protoc.* **13**, 1897–1916 (2018).

77. R. Bruderer, O. M. Bernhardt, T. Gandhi, S. M. Miladinovic, L. Y. Cheng, S. Messner, T. Ehrenberger, V. Zanotelli, Y. Butscheid, C. Escher, O. Vitek, O. Rinner, L. Reiter, Extending the limits of quantitative proteome profiling with data-independent acquisition and

application to acetaminophen-treated three-dimensional liver microtissues. *Mol. Cell. Proteomics* **14**, 1400–1410 (2015).

78. S. Tyanova, T. Temu, P. Sinitcyn, A. Carlson, M. Y. Hein, T. Geiger, M. Mann, J. Cox, The Perseus computational platform for comprehensive analysis of (prote)omics data. *Nat. Methods* **13**, 731–740 (2016).

79. P. V. Hornbeck, B. Zhang, B. Murray, J. M. Kornhauser, V. Latham, E. Skrzypek, PhosphoSitePlus, 2014: Mutations, PTMs and recalibrations. *Nucleic Acids Res.* **43**, D512–D520 (2015).

80. C. Gene Ontology, The Gene Ontology resource: Enriching a GOld mine. *Nucleic Acids Res.* **49**, D325–D334 (2021).

81. Z. Pang, Y. Lu, G. Zhou, F. Hui, L. Xu, C. Viau, A. F. Spigelman, P. E. MacDonald, D. S. Wishart, S. Li, J. Xia, MetaboAnalyst 6.0: Towards a unified platform for metabolomics data processing, analysis and interpretation. *Nucleic Acids Res.* **52**, W398–W406 (2024).

82. Y. Perez-Riverol, C. Bandla, D. J. Kundu, S. Kamatchinathan, J. Bai, S. Hewapathirana, N. S. John, A. Prakash, M. Walzer, S. Wang, J. A. Vizcaino, The PRIDE database at 20 years: 2025 update. *Nucleic Acids Res.* **53**, D543–D553 (2025).

83. F. Liu, E. Y. Luo, D. B. Flora, A. R. Mezo, A synthetic route to human insulin using isoacyl peptides. *Angew. Chem. Int. Ed. Engl.* **53**, 3983–3987 (2014).

84. L. Schaffer, R. E. Brissette, J. C. Spetzler, R. C. Pillutla, S. Ostergaard, M. Lennick, J. Brandt, P. W. Fletcher, G. M. Danielsen, K. C. Hsiao, A. S. Andersen, O. Dedova, U. Ribel, T. Hoeg-Jensen, P. H. Hansen, A. J. Blume, J. Markussen, N. I. Goldstein, Assembly of high-affinity insulin receptor agonists and antagonists from peptide building blocks. *Proc. Natl. Acad. Sci. U.S.A.* **100**, 4435–4439 (2003).

85. J. Nielsen, J. Brandt, T. Boesen, T. Hummelshoj, R. Slaaby, G. Schluckebier, P. Nissen, Structural investigations of full-length insulin receptor dynamics and signalling. *J. Mol. Biol.* **434**, 167458 (2022).

86. N. S. Kirk, Q. Chen, Y. G. Wu, A. L. Asante, H. Hu, J. F. Espinosa, F. Martinez-Olid, M. B. Margetts, F. A. Mohammed, V. V. Kiselyov, D. G. Barrett, M. C. Lawrence, Activation of the human insulin receptor by non-insulin-related peptides. *Nat. Commun.* **13**, 5695 (2022).

87. K. D. Copps, M. F. White, Regulation of insulin sensitivity by serine/threonine phosphorylation of insulin receptor substrate proteins IRS1 and IRS2. *Diabetologia* **55**, 2565–2582 (2012).

88. B. Kim, P. S. Leventhal, M. F. White, E. L. Feldman, Differential regulation of insulin receptor substrate-2 and mitogen-activated protein kinase tyrosine phosphorylation by phosphatidylinositol 3-kinase inhibitors in SH-SY5Y human neuroblastoma cells. *Endocrinology* **139**, 4881–4889 (1998).

89. M. F. White, IRS proteins and the common path to diabetes. *Am. J. Physiol. Endocrinol. Metab.* **283**, E413–E422 (2002).

90. K. Nishida, T. Hirano, The role of Gab family scaffolding adapter proteins in the signal transduction of cytokine and growth factor receptors. *Cancer Sci.* **94**, 1029–1033 (2003).

91. A. Deneka, V. Korobeynikov, E. A. Golemis, Embryonal Fyn-associated substrate (EFS) and CASS4: The lesser-known CAS protein family members. *Gene* **570**, 25–35 (2015).

92. G. H. Kim, E. Park, Y. Y. Kong, J. K. Han, Novel function of POSH, a JNK scaffold, as an E3 ubiquitin ligase for the Hrs stability on early endosomes. *Cell. Signal.* **18**, 553–563 (2006).

93. G. L. Wright, I. G. Maroulakou, J. Eldridge, T. L. Liby, V. Sridharan, P. N. Tsichlis, R. C. Muise-Helmericks, VEGF stimulation of mitochondrial biogenesis: Requirement of AKT3 kinase. *FASEB J.* **22**, 3264–3275 (2008).

94. D. D. Sarbassov, D. A. Guertin, S. M. Ali, D. M. Sabatini, Phosphorylation and regulation of Akt/PKB by the rictor-mTOR complex. *Science* **307**, 1098–1101 (2005).

95. K. Inoki, Y. Li, T. Q. Zhu, J. Wu, K. L. Guan, TSC2 is phosphorylated and inhibited by Akt and suppresses mTOR signalling. *Nat. Cell Biol.* **4**, 648–657 (2002).

96. B. J. Morris, D. C. Willcox, T. A. Donlon, B. J. Willcox, FOXO3: A major gene for human longevity-a mini-review. *Gerontology* **61**, 515–525 (2015).

97. M. C. Vogt, O. Hobert, Starvation-induced changes in somatic insulin/IGF-1R signaling drive metabolic programming across generations. *Sci. Adv.* **9**, eade1817 (2023).

98. C. J. Garwood, L. E. Ratcliffe, S. V. Morgan, J. E. Simpson, H. Owens, I. Vazquez-Villasenor, P. R. Heath, I. A. Romero, P. G. Ince, S. B. Wharton, Insulin and IGF1 signalling pathways in human astrocytes in vitro and in vivo; characterisation, subcellular localisation and modulation of the receptors. *Mol. Brain* **8**, 51 (2015).

99. P. J. Nielsen, G. Thomas, J. L. Maller, Increased phosphorylation of ribosomal protein S6 during meiotic maturation of Xenopus oocytes. *Proc. Natl. Acad. Sci. U.S.A.* **79**, 2937–2941 (1982).

100. A. Biever, E. Valjent, E. Puighermanal, Ribosomal protein S6 phosphorylation in the nervous system: From regulation to function. *Front. Mol. Neurosci.* **8**, 75 (2015).

101. M. Chakkour, M. L. Greenberg, Insights into the roles of inositol hexakisphosphate kinase 1 (IP6K1) in mammalian cellular processes. *J. Biol. Chem.* **300**, 107116 (2024).

102. M. D. Asmamaw, A. He, L. R. Zhang, H. M. Liu, Y. Gao, Histone deacetylase complexes: Structure, regulation and function. *Biochim. Biophys. Acta. Rev. Cancer* **1879**, 189150 (2024).

103. Y. Liu, H. Song, Y. Zhou, X. Ma, J. Xu, Z. Yu, L. Chen, The oncogenic role of protein kinase D3 in cancer. *J. Cancer* **12**, 735–739 (2021).

104. M. D. Houslay, D. R. Adams, PDE4 cAMP phosphodiesterases: Modular enzymes that orchestrate signalling cross-talk, desensitization and compartmentalization. *Biochem. J.* **370**, 1–18 (2003).

105. M. Z. Khan, J. L. Zugaza, I. Torres Aleman, The signaling landscape of insulin-like growth factor 1. *J. Biol. Chem.* **301**, 108047 (2025).

106. M. A. M. Ali, DEAD-box RNA helicases: The driving forces behind RNA metabolism at the crossroad of viral replication and antiviral innate immunity. *Virus Res.* **296**, 198352 (2021).

107. R. Shishkov, T. Chervenkov, T. Yamashima, A. B. Tonchev, Expression of Cyclon/CCDC86, a novel nuclear protein, in the hippocampus of adult non-human primates. *J. Neuroimmunol.* **258**, 96–99 (2013).

108. R. Bai, R. Wan, C. Yan, Q. Jia, J. Lei, Y. Shi, Mechanism of spliceosome remodeling by the ATPase/helicase Prp2 and its coactivator Spp2. *Science* **371**, eabe8863 (2021).

109. A. A. Ayanlaja, Y. Xiong, Y. Gao, G. Q. Ji, C. X. Tang, Z. A. Abdullah, D. S. Gao, Distinct features of doublecortin as a marker of neuronal migration and its implications in cancer cell mobility. *Front. Mol. Neurosci.* **10**, 199 (2017).

110. A. Guilherme, N. A. Soriano, S. Bose, J. Holik, A. Bose, D. P. Pomerleau, P. Furcinitti, J. Leszyk, S. Corvera, M. P. Czech, EHD2 and the novel EH domain binding protein EHBP1 couple endocytosis to the actin cytoskeleton. *J. Biol. Chem.* **279**, 10593–10605 (2004).
